# Backbone Engineering of Monodisperse Conjugated Polymers via Integrated Iterative Binomial Synthesis

**DOI:** 10.1021/jacs.3c08143

**Published:** 2023-08-21

**Authors:** Jiangliang Yin, Shinyoung Choi, Daniel Pyle, Jeffrey R. Guest, Guangbin Dong

**Affiliations:** † Department of Chemistry, University of Chicago, Chicago, Illinois 60637, United States; ‡ Center for Nanoscale Materials, Argonne National Laboratory, Lemont, Illinois 60439, United States

## Abstract

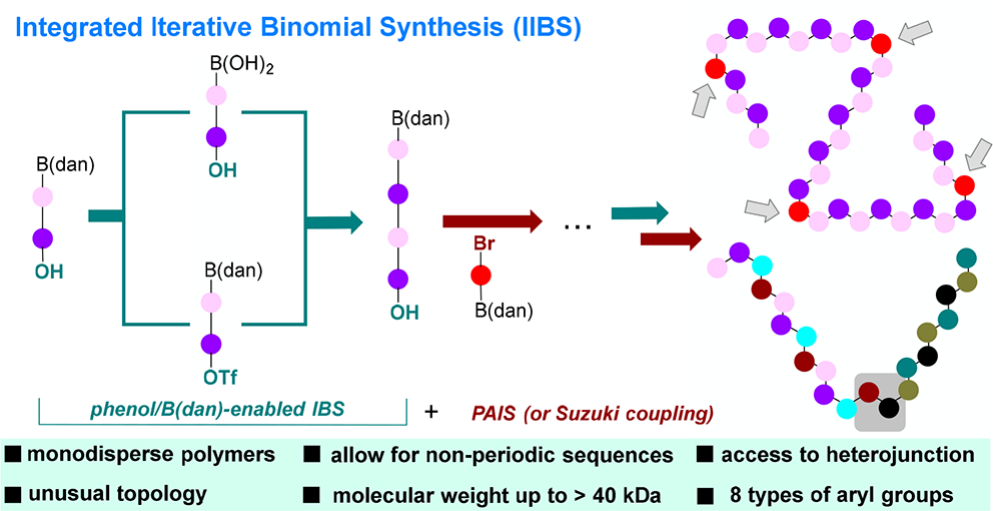

Synthesis of sequence-defined monodisperse π-conjugated polymers with versatile backbones remains a substantial challenge. Here we report the development of an integrated iterative binomial synthesis (IIBS) strategy to enable backbone engineering of conjugated polymers with precisely controlled lengths and sequences as well as high molecular weights. The IIBS strategy capitalizes on the use of phenol as a surrogate for aryl bromide and represents the merge between protecting-group-aided iterative synthesis (PAIS) and iterative binomial synthesis (IBS). Long and monodisperse conjugated polymers with diverse irregular backbones, which are inaccessible by conventional polymerizations, can be efficiently prepared by IIBS. In addition, topology-dependent and chain-length-dependent properties have been investigated.

## Introduction

π-Conjugated polymers have been an important class of organic materials that exhibit appealing electronic and optical properties.^1−4^ Owing to their excellent solution processability, easy accessibility, and high structural tunability, they often serve as promising candidates for use in organic field-effect transistors,^1^ light-emitting diodes,^2^ bulk-heterojunction organic photovoltaics,^1a,3^ optical data storage and nonlinear optics,^1,4^ etc. Intrinsic properties of conjugated polymers are largely dictated by their backbone structures,^5^ molecular weights,^6^ edge substituents,^7^ and polydispersity,^8^ which can directly influence their band structures, solubility, aggregation, formulation rheology, and morphology for both pristine and blended materials. Thus, to meet the growing demand for materials of atomic precision, it has been an attractive goal to realize scalable and robust methods to access discrete, monodisperse, and sequence-defined conjugated polymers.

**Figure 1:**
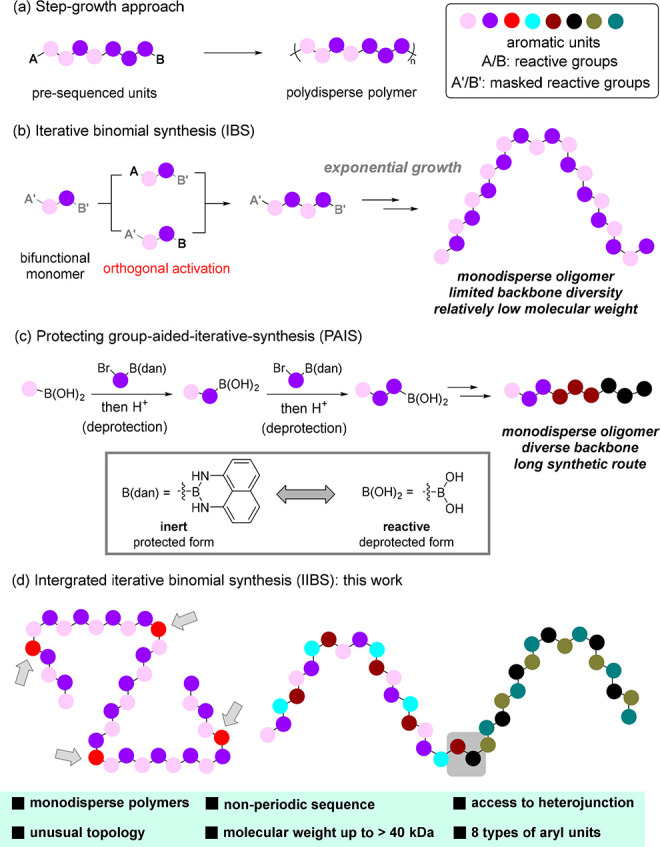
Synthesis of Sequence-defined Conjugated Polymers. dan: 1,8-naphthalenediamine.

Conventionally, conjugated polymers, especially polyphenylene types, are synthesized via transition metal-catalyzed step-growth polymerization.^9,10^ While highly efficient, these approaches generate a mixture of polydisperse products with various chain lengths. The batch-to-batch variations in chain length distributions could lead to inconsistent process-dependent properties of the materials.^11^ Also, sequence control with the step-growth methods can only be achieved by using complex monomers that contain presequenced units (Figure 1a).^10b^ On the other hand, chain-growth polymerization has been elegantly realized with certain monomers to give conjugated polymers with narrow polydispersity, but they generally lack control of absolute mass, chain length, and sequence.^9,12^ Alternatively, the iterative binomial synthesis (IBS) provides a powerful way to access monodisperse oligomers with exponential growth of molecular weights, in which a bifunctional monomer undergoes repetitive orthogonal activation and then pseudohomocoupling to double the chain length in each iteration (Figure 1b).^9,13−16^ However, it remains unclear if IBS can be used to prepare discrete conjugated polymers with a sequence of length other than 2n or irregular sequences in backbones. In addition, the activation methods used in existing IBS strategies to form conjugated systems are usually harsh, such as using solvent amounts of CH_3_I,^14b,14c,15^ strong electrophiles (e.g., ICl, NBS),^16^ and/or strong bases (e.g., *n*-BuLi),^16a^ which unfortunately limits the types of functional units that can be introduced. For example, basic and electron-rich (hetero)arenes cannot tolerate highly electrophilic reagents, and arenes with relatively acidic C–H bonds likely react under strongly basic conditions. Moreover, purification of conjugated polymers from IBS has not been a trivial issue due to similar polarity between starting materials and products; as such, late-stage separations often rely on preparative gel permeation chromatography (GPC).^16^ Furthermore, conjugated oligomers with the same repetitive units in their backbones sometimes suffer from low solubility due to π-aggregation, leading to relatively low molecular weights (i.e., <30 kDa).^13,14b^

Recently, we developed a protecting group-aided-iterative-synthesis (PAIS) strategy to prepare monodisperse oligomers for graphene nanoribbons (GNRs),^17^ in which bifunctional monomers containing a bromide and a 1,8-diaminonaphthalene (dan)-masked boronic acid were used to realize controlled iterative synthesis through the Suzuki–Miyaura coupling (SMC) (Figure 1c).^18,19^ While the PAIS method adds only one monomer in each iteration, high reaction efficiency and chemoselectivity can be achieved by the dan-boronate activation approach. In addition, dan-boronates typically exhibit good solubility. Inspired by these features, we asked that, if an effective *bromide surrogate* can be found and activated under mild conditions, the resulting new bifunctional monomers would enable IBS of monodisperse conjugated polymers with diverse aromatic units. Moreover, the merge of IBS with additional coupling processes (such as PAIS or SMC), namely, the integrated iterative binomial synthesis (IIBS), could offer opportunities to construct versatile and conventionally inaccessible backbones that could provide structural programmability and diversity, as well as tunable properties. Here, we describe the development of the IIBS strategy that allows for backbone engineering of discrete monodisperse conjugated polymers in high efficiency (Figure 1d).

**Figure 2:**
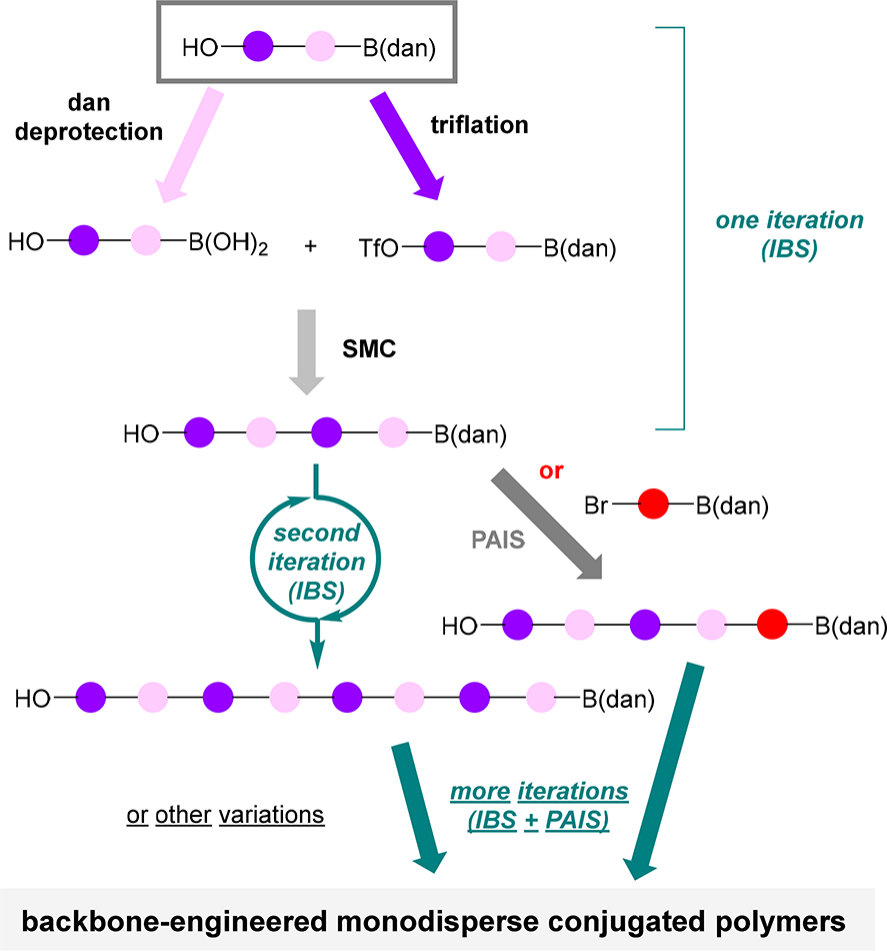
Phenol and B(dan)-enabled IIBS of Diverse Monodisperse Conjugated Polymers.

To realize the IIBS of structurally diverse conjugated polymers, we conceived the idea of using phenol moieties as a surrogate for aryl bromides,^20,21^ which can be activated under orthogonal conditions to B(dan) moieties for cross-couplings. A number of benefits of using phenols can be envisioned. First, phenol hydroxy groups can be efficiently converted to the corresponding triflates (OTf) that possess reactivity similar to that of aryl bromides in the Pd-catalyzed cross-couplings. In addition, phenol moieties typically do not react under both the SMC and B(dan) deprotection conditions. Moreover, the activation process, i.e., triflation of phenols, is rapid, mild, and chemoselective, thus tolerating a wide range of functional groups, including electron-rich and basic (hetero)arenes, as well as B(dan) groups.^21^ Furthermore, the retardation factor (*R*_f_) of the two coupling partners and the resulting bifunctional oligomer segments (BOS) in each iteration are quite distinguishable, rendering a convenient silica gel chromatography purification process. Thus, one can imagine that monomers containing both phenol and B(dan) groups could be effectively employed in the IBS of monodisperse conjugated polymers (Figure 2). Considering that PAIS also uses the same B(dan) deprotection process, this new IBS should smoothly merge with PAIS to achieve IIBS of conjugated polymers with irregular and well-defined sequences. Additionally, different conjugated polymers generated from this approach could couple to each other to access monodisperse heterojunctions (block copolymers).

## Results and Discussion

### Diverse GNR Precursors

**Figure 3:**
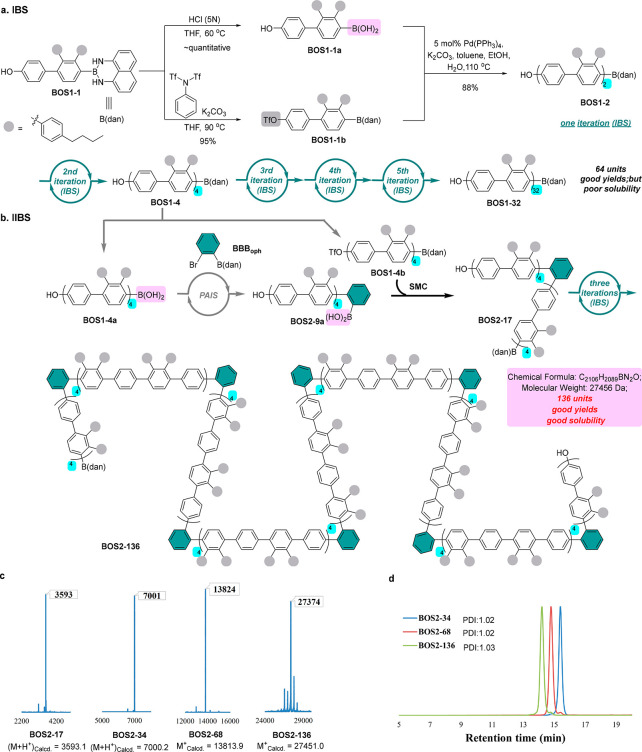
Comparative Syntheses of Polyphenylenes by IBS and IIBS. (a) IBS of monodisperse conjugated polymers with phenylene and *ortho*-terphenylene units. (b) IIBS of long kinked monodisperse polyphenylenes with a controlled sequence and unusual topology. (c) MALDI-TOF-MS spectra of **BOS2-17**, **BOS2-34**, **BOS2-68**, and **BOS2-136**. (d) GPC traces of **BOS2-34**, **BOS2-68**, and **BOS2-136**.

To examine the proposed strategy, we started with the synthesis of monodisperse conjugated polymers with phenylene and *ortho*-terphenylene units (Figure 3a), which are known precursors of *N* = 6 armchair GNRs.^22^ First, the initial substrate (**BOS1-1**) was prepared via SMC of 4-hydroxyphenylboronic acid with the *ortho*-terphenyl bifunctional building block (**BBB**_**o3p**_). Subsequently, **BOS1-1** was divided into two portions with a ratio of 1:1.1. The minor part was treated with HCl under N_2_ atmosphere at 60 °C to give the boronic acid fragment via dan deprotection; the other part was treated with K_2_CO_3_ and *N*-phenyltrifluoromethanesulfonimide to convert the unreactive OH terminus into reactive OTf. Note that the *high tolerance of B(dan) moieties under the triflation conditions is the key to the success of this strategy*. SMC of these two coupling partners was realized to give **BOS1-2** in high yield by using Pd(PPh_3_)_4_ (5 mol %) as the catalyst, K_2_CO_3_ (4 equiv) as the base, and toluene/EtOH/H_2_O (4:1:1) as the solvent at 110 °C. In each SMC step, the length of the oligomer is doubled; given that B(dan) is unreactive under the SMC conditions, no further coupling occurred afterward, which completes one iteration of the IBS. Chain doubling of **BOS1-2** was achieved in the second iteration, involving the parallel dan-deprotection and triflation, followed by the SMC, and the yields remain excellent. The same IBS iteration was repeated a total of five times, affording monodisperse conjugated polymer **BOS1-32** with 64 phenylene units in the backbone. At this stage, further iteration became difficult, as **BOS1-32** exhibits poor solubility in common organic solvents, likely owing to chain aggregation, which is an anticipated limitation with the IBS approach (vide supra).

To address the solubility issue and to introduce structural varieties and previously inaccessible topology, the IIBS approach via merging PAIS and IBS was explored next (Figure 3b). From the **BOS1-4** intermediate described above, the corresponding boronic acid (**BOS1-4a**) and triflate (**BOS1-4b**) can be easily prepared. Instead of coupling these two fragments to form **BOS1-8**, a PAIS process, i.e., the chain homologation with an *ortho*-phenylene monomer (**BBB**_**oph**_) and then SMC with triflate **BOS1-4b**, was employed to interrupt the IBS process. As a result, a 120° kink was introduced to the backbone to give V-shaped oligomer **BOS2-17**, which then served as a starting point to undergo three iterations of IBS to ultimately provide monodisperse **BOS2-136** with a distinct backbone. The molecular weights of the corresponding BOSs in all iterations were confirmed by matrix-assisted laser desorption/ionization-time-of-flight mass spectrometry (MALDI-TOF-MS) and end group analysis using ^1^H NMRs (Figure 3c and Supporting Information). GPC traces of **BOS2-34**, **BOS2-68**, and **BOS2-136** indicate extremely narrow polydispersity indexes (PDIs), which are 1.02, 1.02, and 1.03 respectively (Figure 3d). These results support the unimolecular nature of these conjugated polymers. It is noteworthy that good solubility remains even with the final polymer **BOS2-136**, likely because of the multikinked backbone, which represents a unique advantage of the IIBS over the IBS.^21^ Owing to the distinct *R*_f_ values of the BOS products from their starting materials, all of the polymers obtained here can be conveniently purified by regular silica gel chromatography.

**Figure 4:**
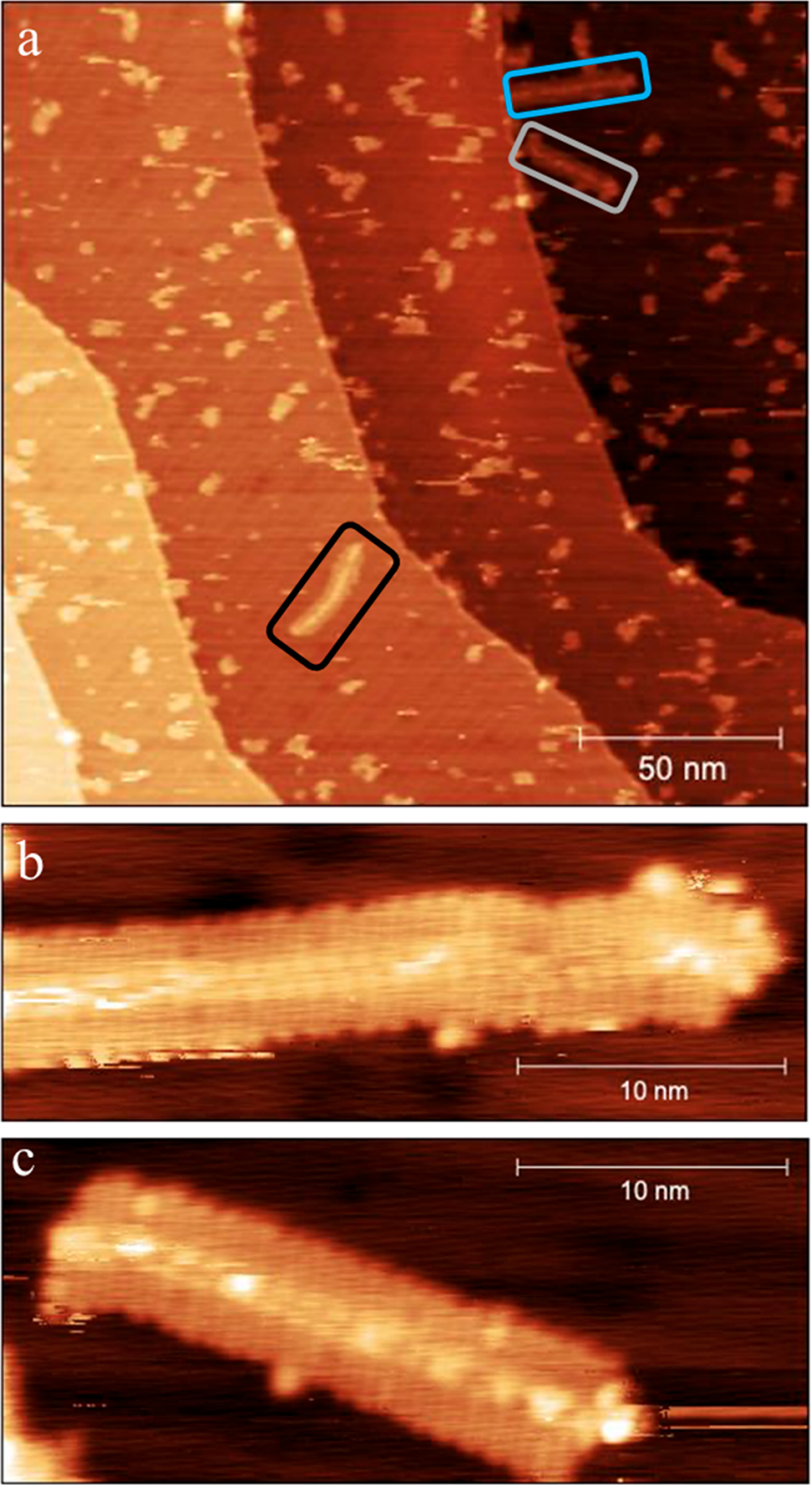
STM Images of Kinked GNR Precursor **BOS2-136**. (a) Large-scale STM topograph (*V* = 2.0 V, *I* = 30 pA) shows monodisperse **BOS2-136** on Au(111). (b) Close-up STM image (*V* = 2 V, *I* = 300 pA) of **BOS2-136** (blue boxed area in a). (c) Close-up STM image (*V* = 2 V, *I* = 300 pA) of **BOS2-136** (gray boxed area in a).

Scanning tunneling microscopy (STM) of the kinked GNR precursor **BOS2-136** was next measured to investigate its behavior on a Au(111) surface under ultrahigh vacuum (2 × 10^–11^ mbar) at 50 K (Figure 4). The resulting monodisperse **BOS2-136** sample was first transferred to a clean Au(111) surface (which was prepared through several sputter/anneal cycles) via the matrix-assisted direct (MAD) transfer technique using pyrene as the matrix (for details, see Supporting Information).^17,23^ The Au(111) sample was then heated to *T* = 270 °C for 20 min to sublime the pyrene matrix and to induce diffusion of the polymers over the surface. Large-scale STM scans show that **BOS2-136** can be diffused very well on the Au(111) surface, while the shape of each polymer varied because of the rotation of the backbone at the kinked positions (Figure 4a). Intriguingly, several well-shaped strips with similar widths (5 nm) were formed (Figure 4a). The middle of these strips appears higher than their edges, which was further confirmed by the STM images of higher resolution (Figure 4b and 4c). Two arrays of bright spots with the same distance, which could be the corresponding ortho-kinks, lie in the middle of these strips (Figure 4b and 4c). While it is difficult to explain how these regular packing structures were formed at this stage, this observation is consistent with the unimolecular nature of this polymer.

**Figure 5:**
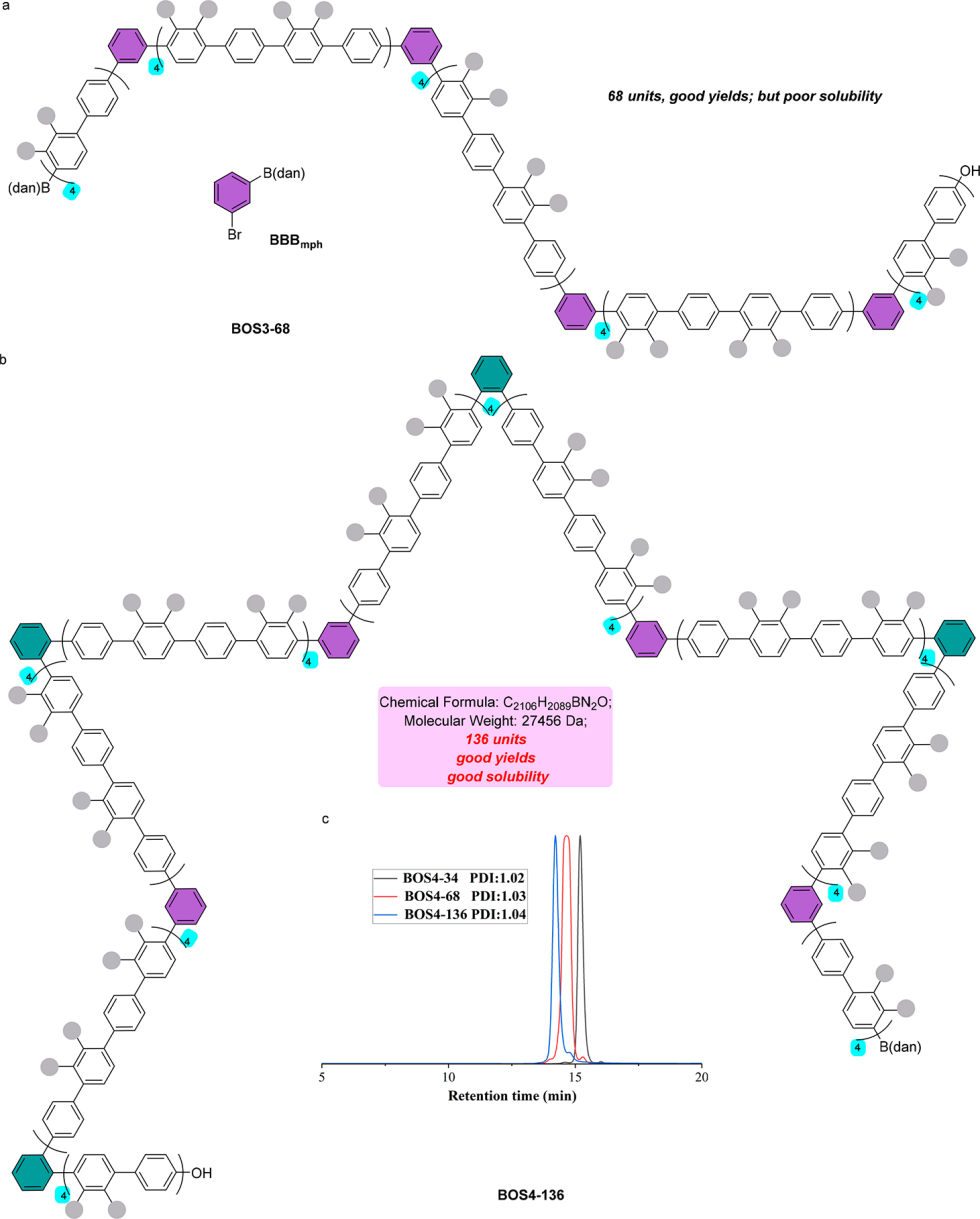
Syntheses of Polyphenylenes with Different Kinds of Kinks. (a) Meta-kinked monodisperse polyphenylenes synthesized by IIBS. (b) Polyphenylenes with alternating ortho- and meta-kinks synthesized by IIBS. For their syntheses, see Supporting Information. (c) GPC traces of **BOS4-34**, **BOS4-68**, and **BOS4-136**.

Since the ortho-kinked monodisperse polyphenylenes were prepared successfully, syntheses of the meta-kinked polyphenylenes and the hybrid ones with alternating ortho- and meta-kinks were then conducted to investigate their differences in solubility and other properties. The meta-kinked polyphenylenes were obtained simply by replacing the ortho-substituted bifunctional building block **BBB**_**oph**_ used in **BOS2-136** with the meta-substituted building block **BBB**_**mph**_ (Figure 5a). While the IIBS approach was still very effective in synthesizing this series of polymers, the final polymer obtained only contained 68 phenylene units due to its limited solubility (Figure 5a). The synthesis of monodisperse polymers with alternating ortho- and meta-kinks by IIBS turned out to be remarkably successful, delivering the final polymer with 136 building blocks (Figure 5b and 5c). These results indicate that the ortho-kinks can significantly improve the solubility of the corresponding polymers, whereas the meta-kinks cannot. To gain insight into the electronic properties of these monodisperse polymers that contain different types of kinks, solution cyclic voltammetry (CV) measurements were then performed. Samples **BOS2-34**, **BOS3-34**, and **BOS4-34**, which have the same number of phenylene units but with different backbone topologies, were chosen as the model substrates. From the CV curves of **BOS2-34**, **BOS3-34**, and **BOS4-34** (see Supporting Information for details), the HOMO energy levels have been calculated as −5.36 eV, −5.43 eV, and −5.40 eV, respectively, indicating that the introduction of ortho-kinks gives a lower HOMO energy level than the one with meta-kinks. Moreover, the observation of an additional reduction state for **BOS2-34** suggests a decrease in electrical stability due to the introduction of ortho-kinks into the polymer backbones.

### Conjugated Polymers Containing Heteroarenes

**Figure 6:**
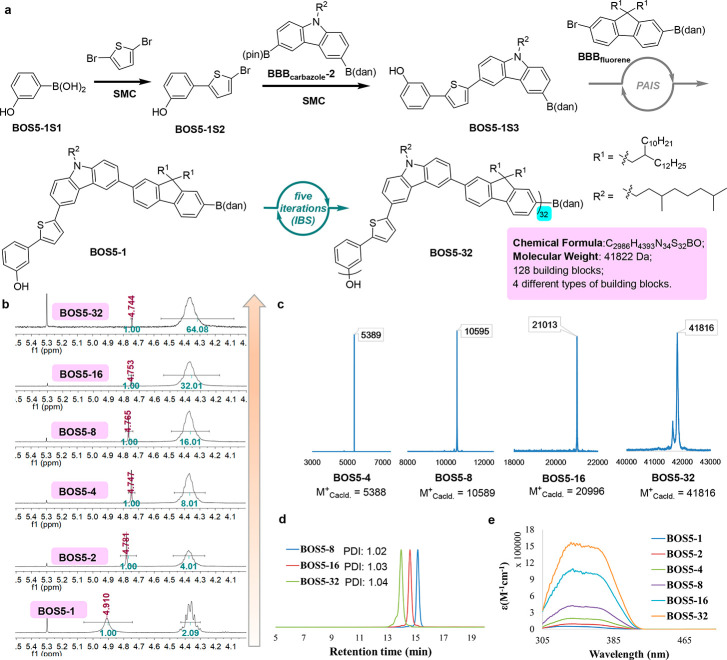
Synthesis and Characterization of Monodisperse Conjugated Polymers with Multiple Types of Heteroaromatics. (a) Preparation of **BOS5** by merging PAIS/SMC with IBS. (b) End group analysis of **BOS5-1**, **BOS5-2**, **BOS5-4**, **BOS5-8**, **BOS5-16**, and **BOS5-32** by ^1^H NMR. (c) MALDI-TOF-MS spectra of **BOS5-4**, **BOS5-8**, **BOS5-16**, and **BOS5-32**. (d) GPC traces of **BOS5-8**, **BOS5-16**, and **BOS5-32**. (e) UV–vis absorption spectra of **BOS5-1**, **BOS5-2**, **BOS5-4**, **BOS5-8**, **BOS5-16**, and **BOS5-32** in CH_2_Cl_2_ at room temperature.

Heteroarenes are commonly found in functional conjugated polymers for various applications, and it is clear that the pattern and sequence of heteroarenes in the polymer backbones can greatly affect their redox, electronic, and optical properties.^1,5^ However, synthesis of monodisperse conjugated polymers containing multiple different heteroarenes in a well-defined sequence remains an unsolved problem, which, we envision, could also be addressed by the merge of PAIS and the phenol/B(dan)-based IBS. The approach is to use PAIS (or SMC) to prepare the initial complex BOS that possesses terminal phenol and B(dan) moieties and then to use IBS to generate the long, conjugated polymer. To test this idea, **BOS5-1** that contains four aromatic units, including benzene, thiophene, carbazole, and fluorene, was synthesized by a sequence of two regular SMC reactions and the PAIS from the commercially available 3-hydroxybenzeneboronic acid (Figure 6a, and Supporting Information for details). Gratifyingly, IBS with **BOS5-1** occurred smoothly, and after five iterations, the desired monodisperse polymer **BOS5-32** with 128 building blocks in total and a molecular weight of 41822 Da was prepared in an efficient manner, which, to the best of our knowledge, is the record for monodisperse conjugated polymers. After each iteration, the resulting polymers were characterized by ^1^H NMR (Figure 6b). The hydroxyl group of the products shows a sharp and distinguishable peak (chemical shifts for **BOS5-1**, **BOS5-2**, **BOS5-4**, **BOS5-8**, **BOS5-16**, and **BOS5-32** are 4.91, 4.78, 4.75, 4.77, 4.75, and 4.74 ppm, respectively), and the integration ratios between the OH and the [NCH_2_]_n_ are 1:2, 1:4, 1:8, 1:16, 1:32 and 1:64 respectively, revealing the expected monodispersity. MALDI-TOF-MS of these compounds was also performed to verify the corresponding molecular weight (Figure 6c). From the GPC traces of **BOS5-8**, **BOS5-16**, and **BO53-32**, the PDIs were calculated as 1.02, 1.03, and 1.04, respectively, which are consistent with the unimolecular nature of these polymers (Figure 6d). With these monodisperse conjugated polymers in hand, their UV–vis absorptions were measured in CH_2_Cl_2_, which indicates that the elongation of the polymer backbone leads to a slight bathochromic shift in light absorption and a largely increased molar extinction coefficient (Figure 6e and Supporting Information).

### Donor–Acceptor Conjugated Polymers

**Figure 7:**
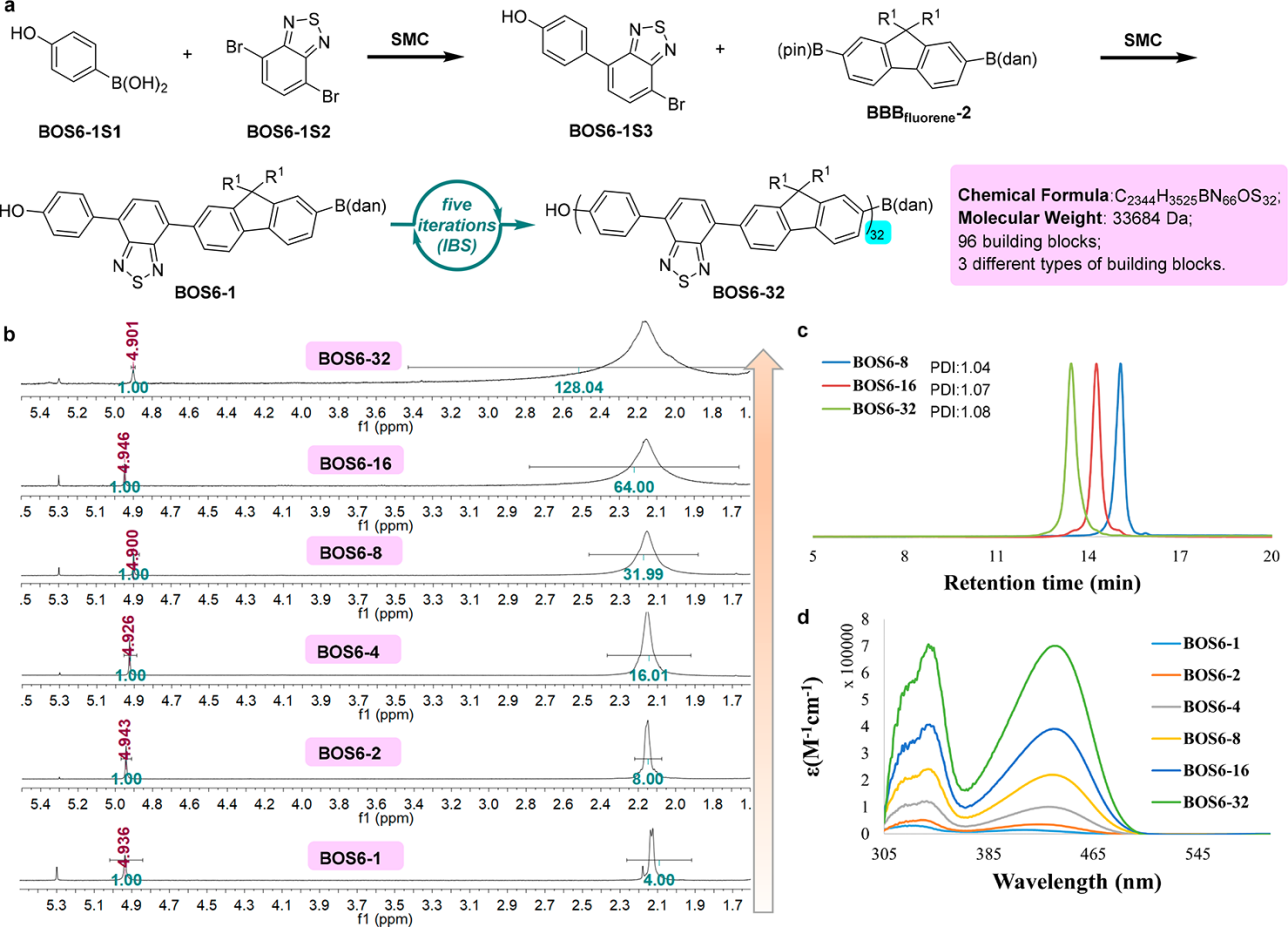
Synthesis and Characterization of Monodisperse Conjugated Polymers with a D–A Structure. (a) Preparation of **BOS6** by merging PAIS with IBS. (b) End group analysis of **BOS6-1**, **BOS6-2**, **BOS6-4**, **BOS6-8**, **BOS6-16**, and **BOS6-32** by ^1^H NMR. (c) GPC traces of **BOS6-8**, **BOS6-16**, and **BOS6-32**. (d) UV–vis absorption spectra of **BOS6-1**, **BOS6-2**, **BOS6-4**, **BOS6-8**, **BOS6-16**, and **BOS6-32** in CH_2_Cl_2_ at room temperature.

The same IIBS strategy can also be used to prepare monodisperse donor–acceptor conjugated polymers^24^ with a defined alternating array of aromatic building blocks (Figure 7). First, the commonly used acceptor benzothiazole and donor fluorene were incorporated as the building blocks into the initial BOS (**BOS6-1**), which was prepared via SMC from the commercially available 4-hydroxybenzeneboronic acid (Figure 7a). **BOS6-1** was then subjected to IBS, providing **BOS6-32** successfully after 5 iterations. As shown in Figure 7b, end group analysis of the BOSs after each iteration shows that the integration ratios between the phenol OH and the [CH_2_CCH_2_]_*n*_ perfectly match the expected molecular structures, suggesting high purity of these products. The single and sharp peaks from the GPC traces of **BOS6-8**, **BOS6-16**, and **BOS6-32** indicate small PDIs (1.04, 1.07, and 1.08 respectively), which support the expected monodispersity (Figure 7c). The UV–vis absorption of these products exhibits a trend similar to that of the **BOS5** family (Figure 7d).

**Table 1:** Comparison of the Optical and Electronic Properties of the Donor–Acceptor Polymers

compounds	λ_max_ (nm)^a^	ε_max_ (M^–1^ cm^–1^)^a^	*E*_g_^opt^ (eV)^b^	HOMO (eV)^c^	LUMO (eV)^c^	*E*_g_ (eV)^d^
**BOS6**-**1**	415	1.46 × 10^4^	2.62	–	–2.91	–
**BOS6**-**2**	424	3.61 × 10^4^	2.59	–5.47	–2.90	2.57
**BOS6**-**4**	433	10.1 × 10^4^	2.57	–5.44	–2.97	2.47
**BOS6**-**8**	436	22.0 × 10^4^	2.55	–5.41	–2.94	2.47
**BOS6**-**16**	439	39.2 × 10^4^	2.54	–5.40	–2.95	2.45
**BOS6**-**32**	439	70.2 × 10^4^	2.54	–5.40	–3.01	2.39
**BOS(5,6)**-**16**	439	54.8 × 10^4^	2.54	–5.23	–3.01	2.22

a λ_max_ and ε_max_ in DCM.

b Optical band gap calculated according to the onset absorption.

c LUMO and HOMO energy levels are calculated from the solution CV measurements in DCM with ferrocene as the external standard.

d HOMO–LUMO energy gap calculated according to the equation *E*_g_ = (*E*_LUMO_ – *E*_HOMO_) eV.

### Heterostructure Control

**Figure 8:**
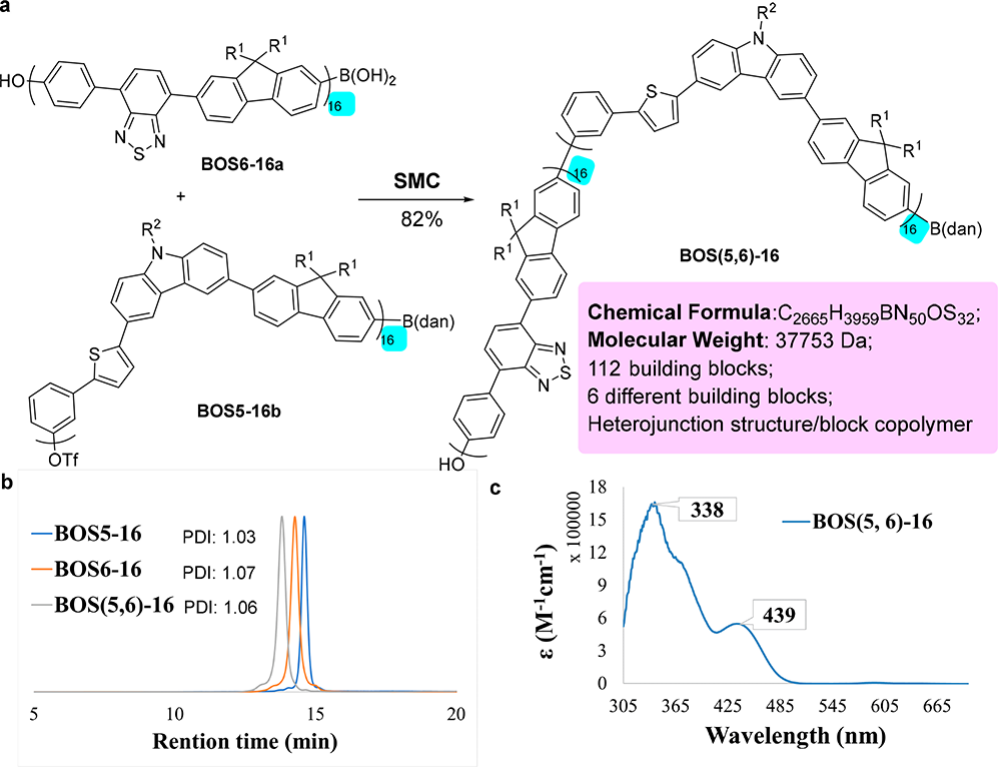
Synthesis and Characterization of a Monodisperse Heterojunction. (a) Synthesis of heterojunction **BOS(5,6)-16** by SMC of two macro segments. (b) GPC trace of **BOS(5,6)-16**. (c) UV–vis absorption spectra of **BOS(5,6)-16** in CH_2_Cl_2_ at room temperature.

Lastly, given that BOS products after each iteration still contain activatable terminus, two macro segments could then be integrated to give monodisperse and sequence-defined block copolymers.^14m^ For example, the SMC coupling of the electron-rich donor fragment (**BOS5-16b**) and the donor–acceptor fragment (**BOS6-16a**) resulted in the unique heterojunction **BOS(5,6)-16** in 82% yield (Figure 8a), which contains six different types of building blocks and 112 units in total. The monodispersity of this polymer was again supported by ^1^H NMR integration analysis and the small PDI (1.06) (Figure 8b). The UV–vis spectrum of **BOS(5,6)-16** has a strong absorption at 338 nm and a moderate peak at around 439 nm, which exhibits the combined optical feature between **BOS5-16** and **BOS6-16** (Figure 8c). In principle, beyond the mono heterojunction, tri-, tetra-, and other multiple-block copolymers could also be synthesized by the same strategy.

### Chain-Length-Dependent Properties

The chain length of conjugated polymers can play a role in modulating their thermotropic, optical and electronic properties.^6,8,14f,14h,16^ With these monodisperse donor–acceptor conjugated polymers in hand, optical and electronic properties were examined (Table 1). The UV–vis absorption measurements indicate that the absorption maximums of **BOS6-1**, **BOS6-2**, **BOS6-4**, **BOS6-8**, **BOS6-16**, **BOS6-32**, and **BOS(5,6)-16** are 415, 424, 433, 436, 439, 439, and 439 nm, respectively, indicating a red shift with the increase of the chain length. The energy gaps obtained from the solution CV measurements showed a similar trend as the optical energy gaps. The heterojunction polymer **BOS(5,6)-16** gave a noticeably lower HOMO energy level compared with that of **BOS6-16** and **BOS6-32**, which could be attributed to the introduction of segment **BOS5-16**.

## Conclusion

In conclusion, we have developed a distinct IIBS approach to prepare monodisperse conjugated polymers with a well-defined sequence and diverse backbones in good yield and high molecular weights. Kinked structures and various classes of aromatic building blocks could be incorporated into the polymer main chains, which provides discrete conjugated polymers that are nearly inaccessible by conventional polymerization methods. The use of phenol as an activatable terminus shows multifold advantages, compared to other IBS approaches, which could have implications beyond this IIBS method. Exploration of the utilities of these monodisperse polymers prepared here and use of IIBS to prepare more complex architectures are ongoing.

## Supporting Information Available

Additional experimental details, materials, methods and spectral data (PDF)
