# Supplementary material for: Backbone Engineering of Monodisperse Conjugated Polymers via Integrated Iterative Binomial Synthesis
Source: J Am Chem Soc. 2023 Aug 21;145(34):19120–8. doi: 10.1021/jacs.3c08143 (PMC10472507; doi:10.1021/jacs.3c08143)

Supporting Information

Backbone Engineering of Monodisperse Conjugated Polymers via Integrated Iterative Binomial Synthesis

Jiangliang Yin[1], Shinyoung Choi[1], Daniel Pyle[1], Jeffrey R. Guest[2], and Guangbin Dong[1*]

[1]Department of Chemistry, University of Chicago, Chicago, IL 60637, USA

[2]Center for Nanoscale Materials, Argonne National Laboratory, Lemont, IL 60439, USA.

Contents

I. General remarks 2

II. Synthesis of bifunctional building blocks and their derivatives 3

- **General procedure for the borylation of dibromoaromatics**[2] 3
- **General procedure for the dan protection** 3
- **General procedure for the borylation of bifunctional building blocks** 3

III. General procedure for the integrated iterative binomial synthesis 4

- **General procedure A for the SMC** 4
- **General procedure B for the SMC** 4
- **General procedure for the deprotection** 4
- **General procedure A for the triflation** 4
- **General procedure B for the triflation** 5

IV. General procedure for the STM measurements 14

- **General procedure for preparation of MAD transfer samples** 14
- **Sample preparation** 14
- **STM measurements** 14

V. Characterization of the synthesized compounds 15

VI. UV-Vis absorption measurements of the representative products 46

VII. GPC measurements of the representative products 49

VIII. Cyclic voltammetry (CV) measurements 55

IX. References 61

X. NMR spectra 62

I. General remarks

NMR spectra were recorded on a Bruker Model DMX 400 spectrometer. The ^{1}H NMR (400 MHz) chemical shifts were recorded relative to $CDCl_3$ as the internal reference ($CDCl_3$: δ_H = 7.26 ppm). The ^{13}C NMR (100 MHz) chemical shifts were given using $CDCl_3$ as the internal standard ($CDCl_3$: δ_C = 77.16 ppm). High-resolution mass spectra (HRMS) were obtained on an Agilent 6530 LCQ-TOF mass spectrometer using electrospray ionization with a fragmentation voltage set at 115 V and processed with an Agilent MassHunter Operating System. IR spectra experiments were conducted on a on a Nicolet 380 FTIR using the neat thin film technique. Matrix-assisted laser desorption/ionization–time-of-flight (MALDI-TOF) mass spectra were obtained with a Bruker Ultraflextreme MALDI-Tof-Tof instrument in reflection mode or linear mode, with trans-2-[3-(4-tert-Butylphenyl)-2-methyl-2-propenylidene]malononitrile (DCTB) or terthiophene as the matrix, or without using a matrix. Size exclusion chromatography (SEC) for polymer molecular weight analysis (based on polystyrene standard) was carried out with an Agilent 1260 Infinity system (VWD UV detector) and two 300 x 7.5 mm ResiPore GPC columns eluted with THF (HPLC grade, Sigma-Aldrich). Flow rate was 1.0 mL/min and the column temperature was maintained at 35 ºC. UV-Vis spectra were measured on a Agilent Cary 5000 spectrophotometer. The baseline was corrected by subtracting a measurement of the cuvette filled with pure solvent used for the measurement. The samples were measured in CH_2Cl_2 solution at room temperature.

Unless otherwise noted, all reagents were obtained from commercial suppliers and used without further purification. Pd(dppf)Cl_2 and $Pd(PPh_3)_4$ were prepared according to the literature procedures.[1] The solvents were purified and dried using an Inert PS-MD-7 Solvent Purification System. $PdCl_2$ was purchased from Sigma-Aldrich CO., Ltd. Unless otherwise noted, all reactions were performed with dry solvents under a nitrogen atmosphere in a vial.

II. Synthesis of bifunctional building blocks and their derivatives

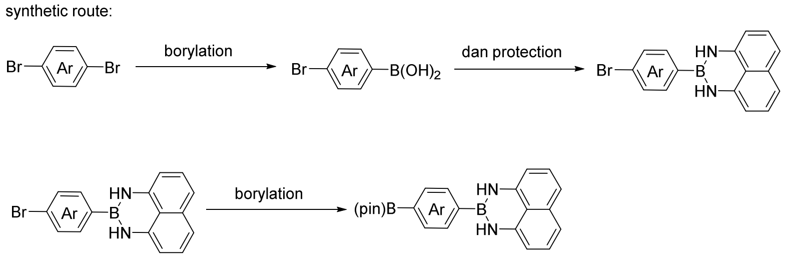

Figure S1. Synthesis of bifunctional building blocks and their derivatives.

General procedure for the borylation of dibromoaromatics[2]

The dibromoarene substrate (10 mmol) was dissolved in dry THF (50 mL) under N_2, and the solution was cooled down to -78 ºC. *n*-BuLi (2.5M, 1.2 equiv.) was added dropwise with stirring, and the mixture was stirred at -78 ºC for 1 h. Then, triisopropyl borate (1.5 equiv.) was added dropwise with stirring. The resulting mixture was stirred at room temperature overnight before HCl (1 M in H_2O, 30 mL) was added. The mixture was then extracted three times with ethyl acetate. The combined organic phase was washed three times with water, dried over anhydrous Na_2SO_4, and concentrated under reduced pressure to give the aryl boronic acid, which was directly used in the dan protection without further purification.

General procedure for the dan protection

A known procedure was followed.[3] Aryl boronic acid substrate (10 mmol), 1,8-diaminonaphthalene (11 mmol) and toluene (20 mL) were added in a vial under air atmosphere, and the mixture was refluxed at 120 ºC for 12 hours. After the reaction was completed, the solvent was removed by rotary evaporation, and the residue was purified by silica gel chromatography using hexanes/DCM as the eluent to give the desired B(dan) product.

General procedure for the borylation of bifunctional building blocks

The bifunctional building block (1.0 equiv.), $B_2(pin)_2$ (1.1 equiv.), [1,1′-bis(diphenylphosphino)ferrocene]dichloropalladium(II) complex (1 mol%), KOAc (2 equiv.), and 1,4-dioxane (0.5M) were added to a vial in a glovebox. The reaction was stirred at 80 ºC for 24 h. The solvent was removed under reduced pressure. The residue was purified by column chromatography on silica gel to provide the desired borylation product.

III. General procedure for the integrated iterative binomial synthesis

General procedure A for the SMC

The coupling partners (the ratio of aryl boronic acids and bifunctional oligomer segments was 1:1.1), [1,1′-bis(diphenylphosphino)ferrocene]dichloropalladium(II) complex (5 mol%), anhydrous K_3PO_4 (4 equiv.), H_2O (7 equiv.) and dry THF (0.1M) were added to a vial in a glovebox. The reaction was stirred at 90 ºC for 12 h. The solvent was removed under reduced pressure. The residue was purified by column chromatography on silica gel to provide the desired product. Note: The control of stoichiometry was achieved by using solutions of the polymer substrates. For example, we first dissolved the 32-mer polymer in 21 mL DCM, which was then divided into two portions with a ratio of 1:1.1 (10 mL: 11mL). By using this method, the stoichiometry can be controlled very well.

General procedure B for the SMC

The coupling partners (the ratio of aryl boronic acids and bifunctional oligomer segments was 1:1.1), tetrakis(triphenylphosphine)palladium (5 mol%), K_2CO_3 (4 equiv.) and a solvent mixture of toluene/ethanol/H_2O (4:1:1) were added to a vial inside a glovebox. The reaction was then stirred at 110 ºC for 12 h. The solvent was removed under reduced pressure. The resulting residue was purified by column chromatography on silica gel to yield the desired product.

General procedure for the B(dan) deprotection

A vial was charged with the B(dan) compound (1 mmol) under air, and then transferred to a glovebox. After adding HCl (5 M in H_2O, 15 equiv.) and dry THF (10 mL), the vial was removed from the glovebox and the reaction was stirred at 60 ºC for 20 h. The mixture was extracted with an EtOAc/hexane mixture (1:1) three times. The organic phases were combined and washed with water three times. When the polymers containing benzothiazole were used as the substrates, the organic phase should be washed with a 1M $NaHCO_3$ aqueous solution three times. After the combined organics were dried with Na_2SO_4 and concentrated by rotary evaporation, the residue was used as is in the next coupling reaction.

General procedure A for the triflation

Triethylamine (1.2 equiv.) was added to a stirred solution of phenol (1.0 equiv.) in dichloromethane (0.25 M) at 0 ºC under air. Then, trifluoromethanesulfonic anhydride (1.1 equiv.) was added to this solution dropwise. The mixture was allowed to stir at

room temperature for 4 hours, before it was concentrated under reduced pressure. The residue was purified by column chromatography on silica gel using DCM and hexane as eluent to provide the triflation products.

General procedure B for the triflation

Phenol substrate (1.0 equiv.), *N*-phenyltrifluoromethanesulfonimide (1.2 equiv.), K_2CO_3 (3.0 equiv.) and dry THF (0.1M) were added to a vial in a glovebox. The vial was taken out of glovebox and the reaction was stirred at 90 ºC for 12 h. The reaction mixture was concentrated under reduced pressure conditions. The residue was purified by column chromatography on silica gel using DCM and hexane as eluent to yield the triflated products.

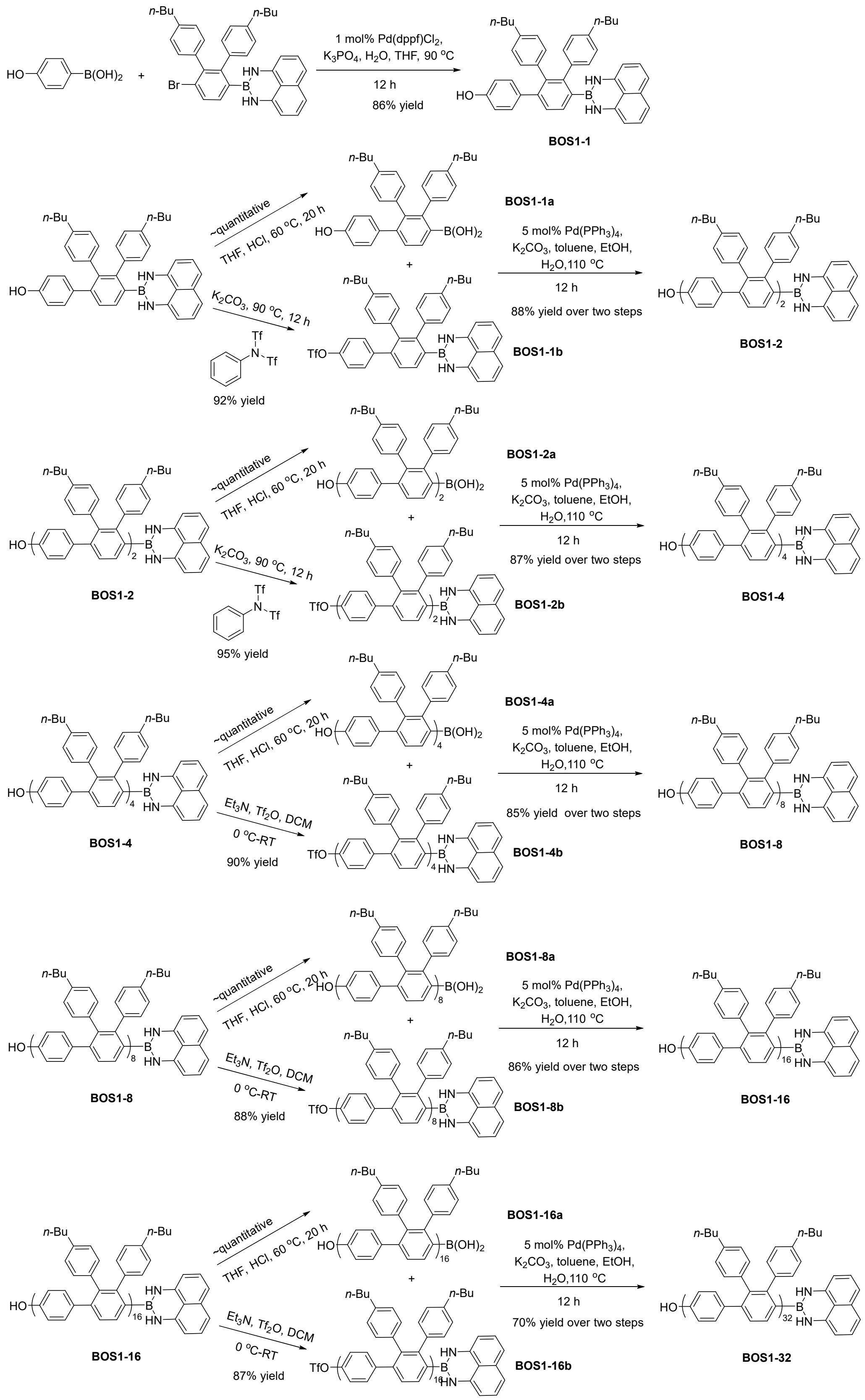

Figure S2. Synthesis of **BOS1**.

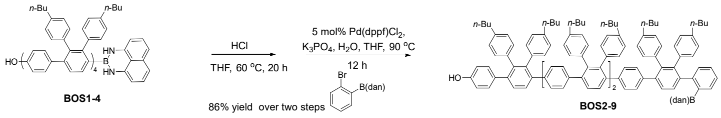

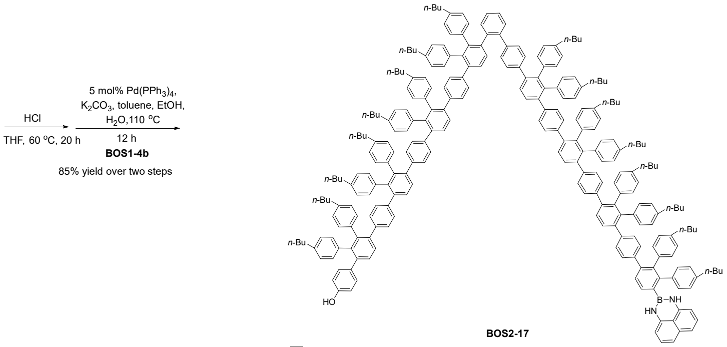

BOS2-17

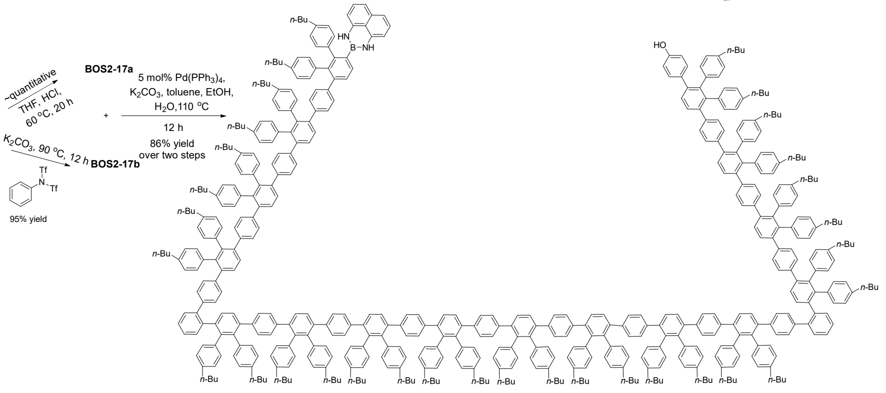

BOS2-34

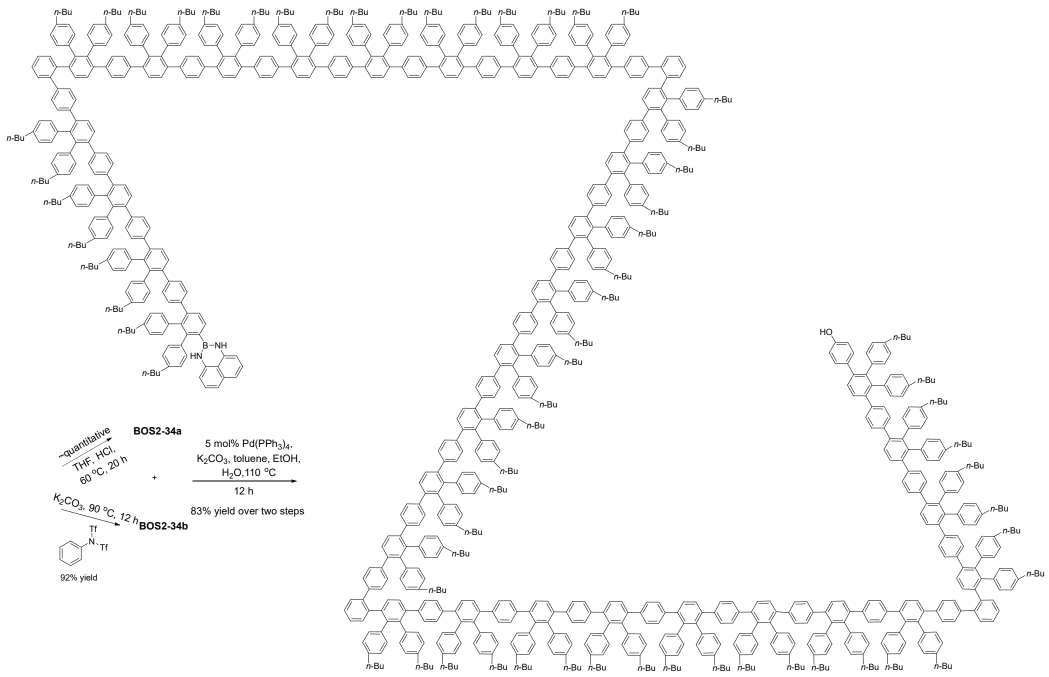

BOS2-68

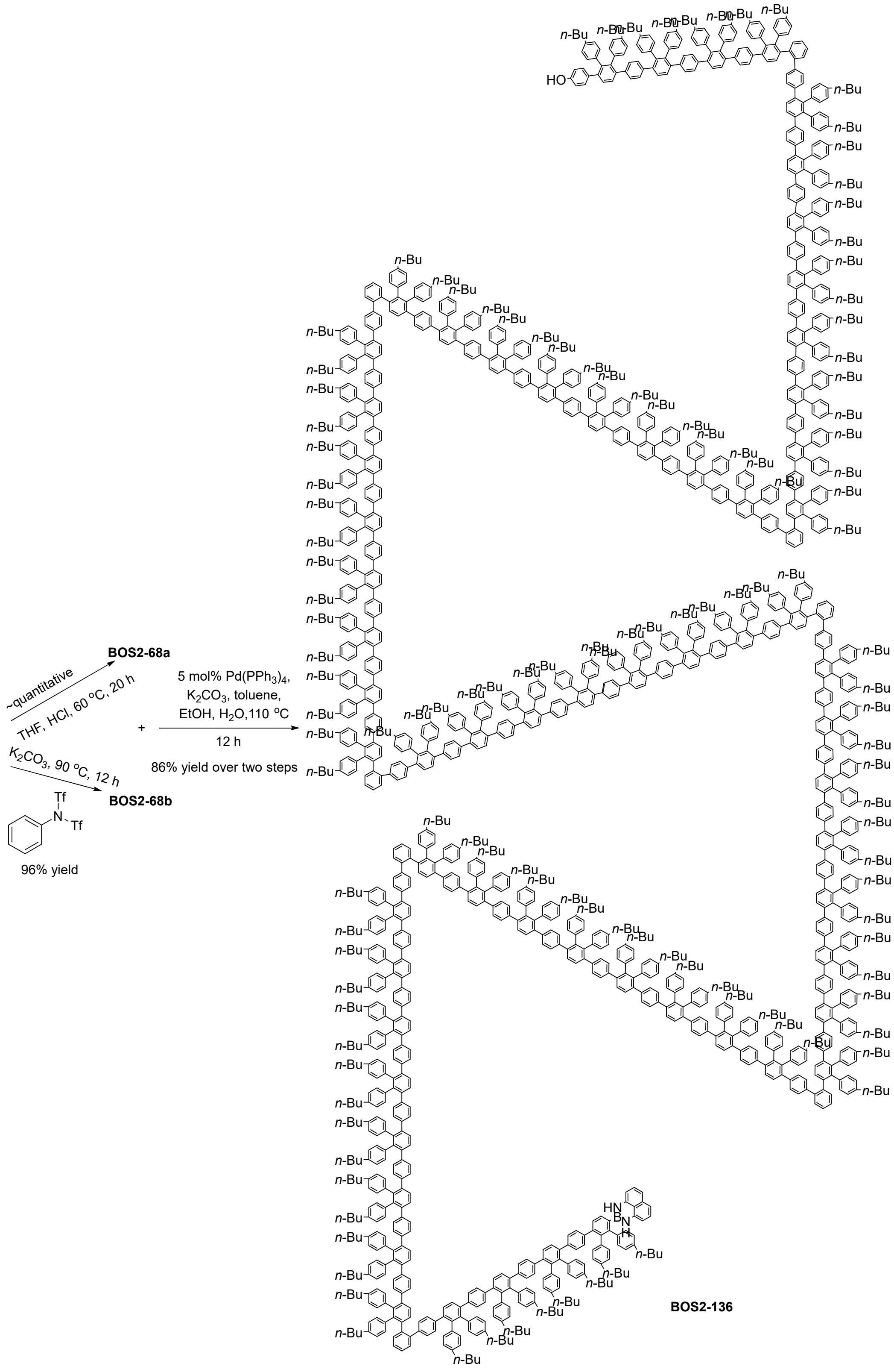

Figure S3. Synthesis of **BOS2**.

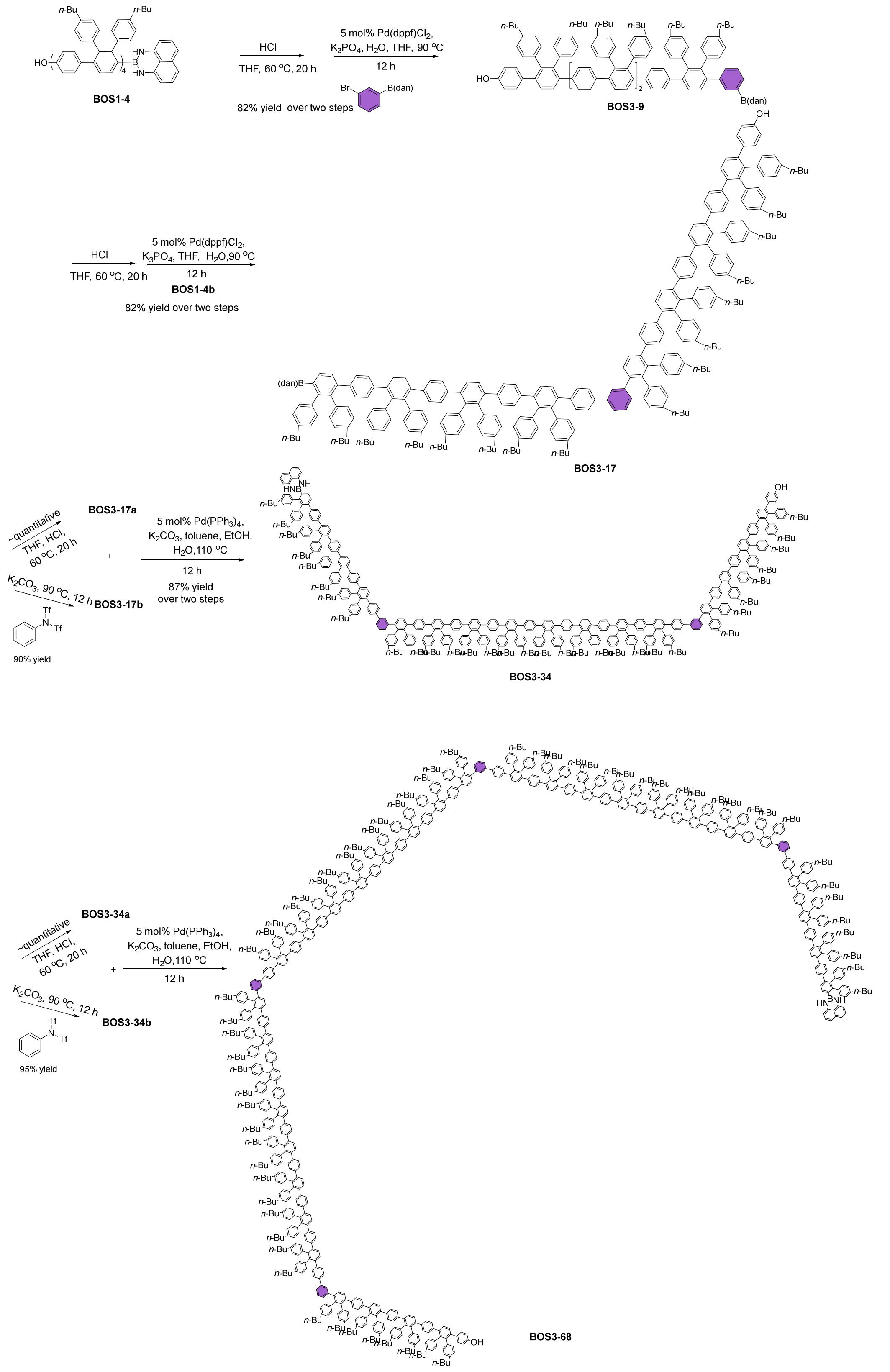

Figure S4. Synthesis of **BOS3**.

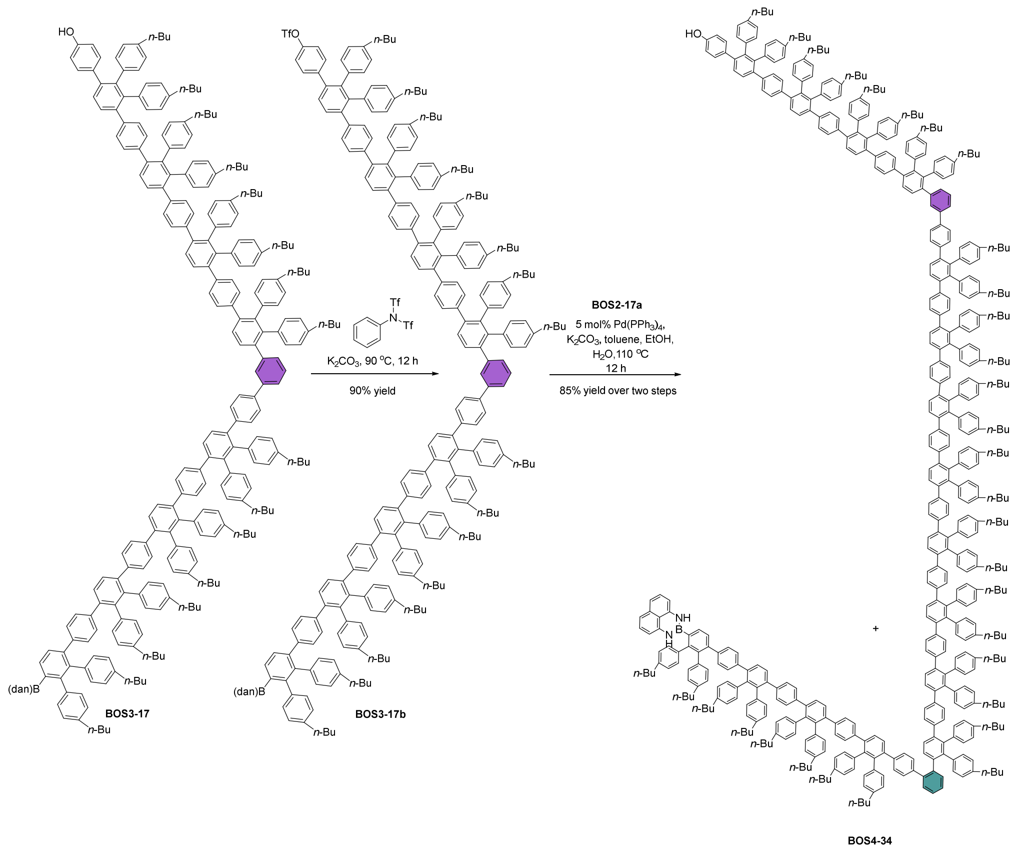
BOS3-17
K_2CO_3, 90 °C, 12 h
90% yield
BOS3-17b
BOS2-17a
5 mol% $Pd(PPh_3)_4$,
K_2CO_3, toluene, EtOH,
H_2O,110 °C
12 h
85% yield over two steps
BOS4-34

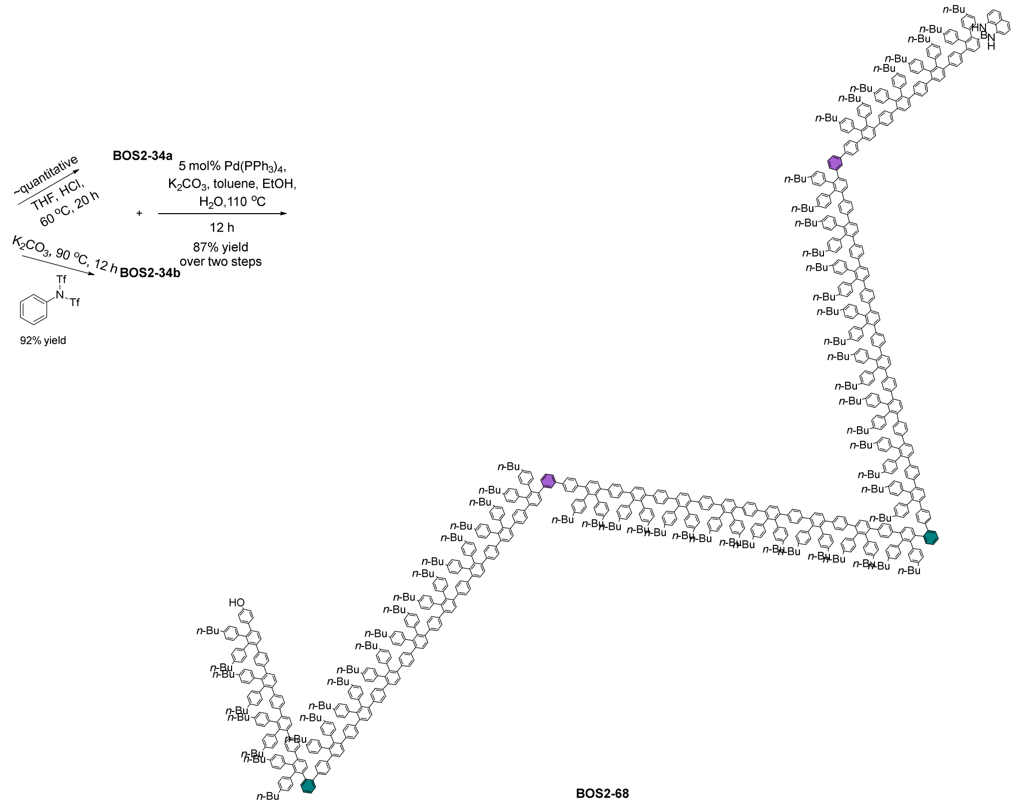
~quantitative
THF, HCl,
60 °C, 20 h
BOS2-34a
K_2CO_3, 90 °C, 12 h
BOS2-34b
92% yield
5 mol% $Pd(PPh_3)_4$,
K_2CO_3, toluene, EtOH,
H_2O,110 °C
12 h
87% yield
over two steps
BOS2-68

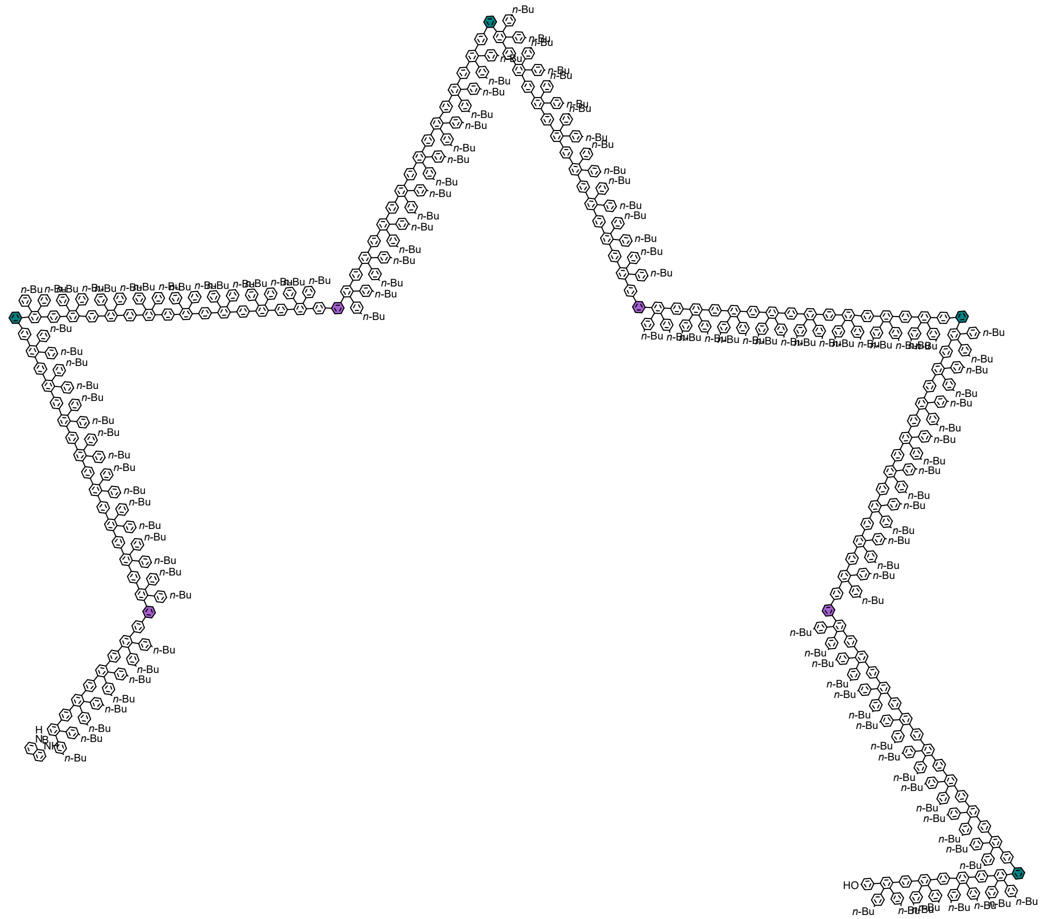

BOS4-136

Figure S5. Synthesis of **BOS4**.

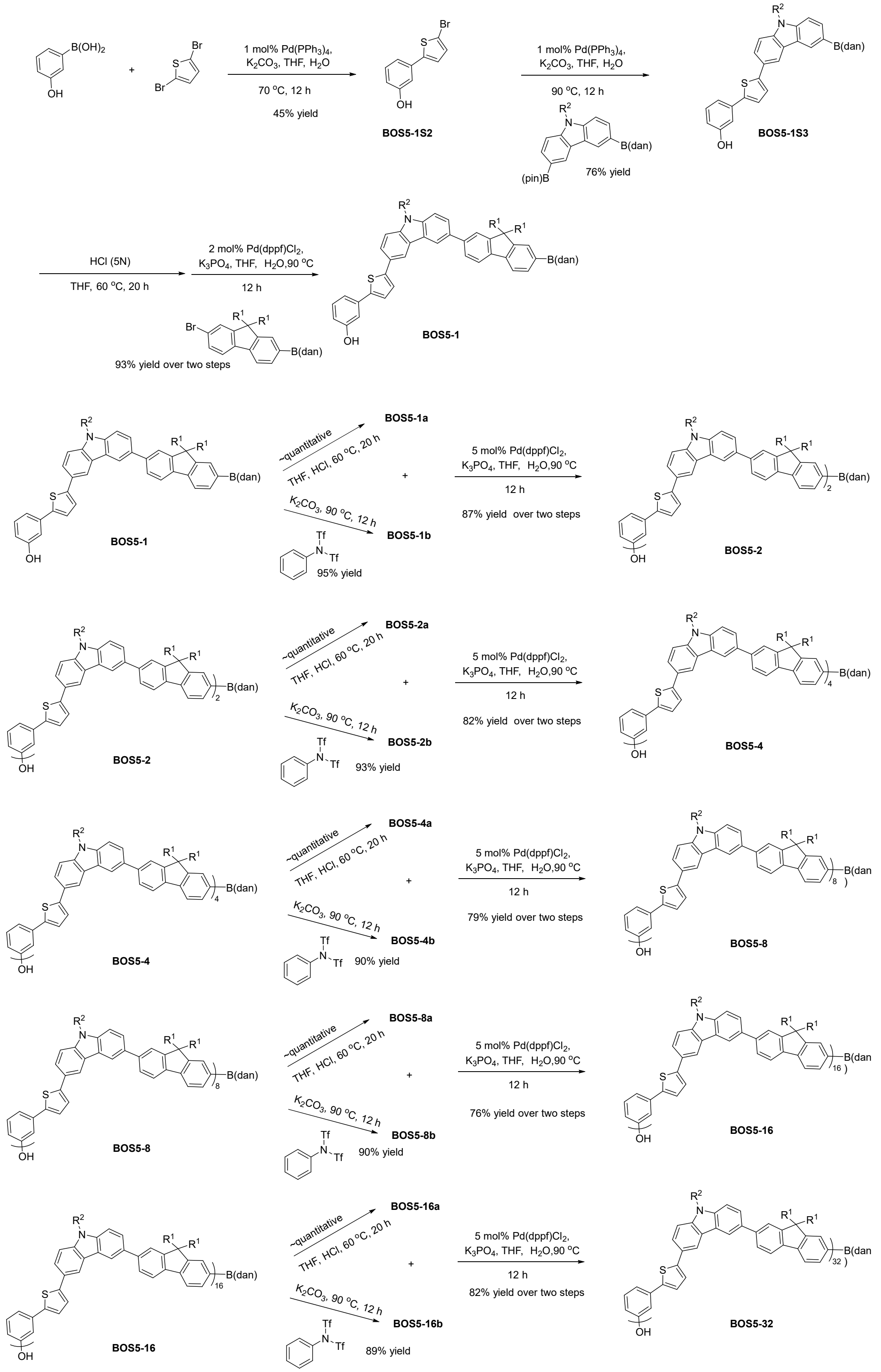

Figure S6. Synthesis of **BOS5**.

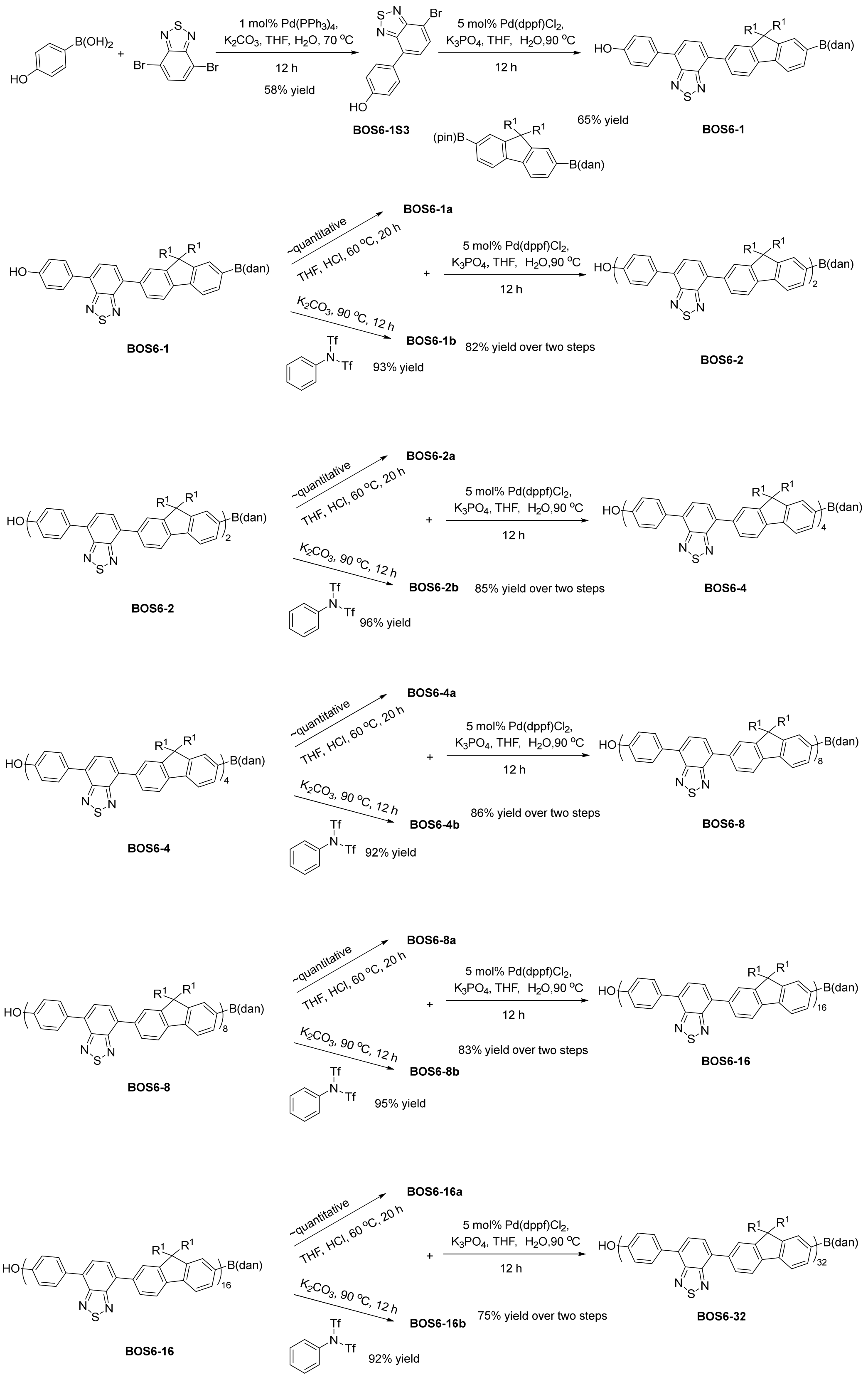

Figure S7. Synthesis of **BOS6**.

IV. General procedure for the STM measurements

General procedure for the preparation of MAD transfer samples

The polymer to be deposited was combined with pyrene at room temperature in a vial to make a 0.1 wt% mixture of sample in pyrene under N_2 atmosphere. The vial was then heated to 180 ºC and the melted mixture was swirled for 5 min to ensure homogeneous dispersion of the polymer in the pyrene melt. The melted mixture was then immediately placed into an acetone/dry ice bath to induce rapid crystallization. The obtained solid was then ground to a fine powder prior to deposition.

Sample preparation

Atomically clean Au(111) was prepared through repeated cycles of argon ion (Ar^+) bombardment and annealing. The polymer was introduced onto the surface through matrix-assisted direct (MAD) transfer using the setup described in our previous work.[2] In each MAD transfer experiment, the glass fiber was cleaned with solvents and subsequently outgassed in high vacuum ($p < 10^{-7}$ mbar) at T = 500 ºC for t = 30 min prior to MAD transfer. It was then removed from the vacuum chamber and the MAD powder was applied to it under ambient conditions. The fiber was then reintroduced into the chamber and the powder transferred onto the surface under vacuum by gently touching the clean gold surface until a barely visible amount of material was observed by eye. After the MAD transfer, the Au(111) samples were heated to T = 270 ºC for t = 20 min, in order to sublimate the pyrene matrix and to promote surface diffusion of the polymers.

STM measurements

All STM experiments were carried out using a commercial Omicron UHV Low Temperature (LT) STM held at T = 50 K using Tungsten tips. Image processing of the STM scans was performed using WSXM software. Tip passivation was performed using standard procedures.

In the large scale STM image, the very small dots are pyrene used as the MAD transfer matrix. The long segments could have many dozens of polymers making up a single one. The shape of each polymer varies because of the rotation of the backbone at the kinked positions.

V. Characterization of the synthesized compounds

Note that ^{13}C NMR spectra for oligomers and polymers were not taken due to the severe overlaps of aromatic signals. By using general matrixes such as DHB, DCTB and terthiophene, the polymers containing benzothiazole don't have obvious signal for MALDI-TOF-MS measurements. Note: the measurement of MALDI-TOF-MS is a chemical process and sometimes it generates fragment peaks. It is generally very difficult to get the MALDI-TOF-MS signals of high-molecular-weight polymers, such as **BOS2-68** and **BOS2-136**. In these cases, we usually increase the laser power to get better signals, but the higher laser power often leads to the fragment peaks at the same time. These fragments give lower mass peaks, and when small fragments couple with each other or with target molecules, higher molar mass peaks could appear. In other words, the observation of other peaks in MADLI-TOF-MS spectra doesn't necessarily indicate that the sample is not pure. Using our IIBS strategy, the monodisperse polymers are formed with an exponential increase in molecular weights. This suggests that potential impurities should have $1/2^n$ or 2^n times of the desired molecular weight. Nevertheless, these peaks were not observed in the MALDI-TOF-MS spectra. Therefore, it is more likely that these smaller peaks in MADLI-TOF-MS spectra originate from fragments generated during the measurements, though we cannot fully rule out the possibility that certain minor peaks may represent other molecular ions.

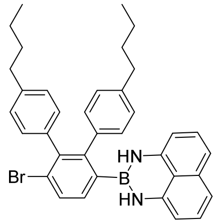

BBB$_{o3p}$

Following the general procedure for the synthesis of bifunctional building blocks, **BBB$_{o3p}$** was obtained in 76% yield (2.7 g) as a colorless gel.

^{1}H NMR ($CDCl_3$, 400 MHz): δ (ppm) 7.73 (d, J = 8.0 Hz, 1H), 7.49 (d, J = 8.0 Hz, 1H), 7.03-6.91 (m, 12H), 6.01 (dd, J_1= 7.2 Hz, J_2 = 0.8 Hz, 2H), 5.32 (s, 2H), 2.55-2.48 (m, 4H), 1.53-1.43 (m, 4H), 1.28-1.19 (m, 4H), 0.91-0.84 (m, 6H).

^{13}C NMR (101 MHz, $CDCl_3$) δ = 148.0, 142.3, 141.7, 141.4, 141.1, 138.7, 137.5, 136.3, 132.5, 131.5, 130.2, 129.9, 127.9, 127.64, 127.58, 126.6, 119.5, 117.6, 105.8, 35.41, 35.35, 33.7, 33.4, 22.3, 22.2, 14.12, 14.10.

^{11}B NMR ($CDCl_3$, 128 MHz): δ (ppm) 31.2.

HRMS: Calcd. For: $C_{36}H_{37}BBrN_2^+$ ($M+H^+$): 587.2228; found 587.2226.

IR: 669, 762, 819, 1032, 1114, 1328, 1373, 1410, 1504, 1600, 1629, 2342, 2360, 2856, 2927, 2955, 3420 cm^{-1}.

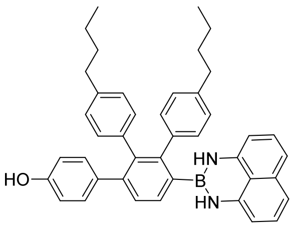

BOC1-1

General procedure A was used for the SMC. **BOC1-1** was obtained in 86% yield (4.8 mmol scale, 1.97 g) as a colorless gel.

R_f = 0.3 (Hexanes/EA = 4:1)

^{1}H NMR ($CDCl_3$, 400 MHz): δ (ppm) 7.71 (d, J = 7.6 Hz, 1H), 7.48 (d, J = 7.6 Hz, 1H), 7.05-6.94 (m, 10H), 6.78-6.70 (m, 4H), 6.63-6.61 (m, 2H), 6.02 (dd, J_1 = 7.2 Hz, J_2 = 1.2 Hz, 2H), 5.38 (s, 2H), 4.61 (s, 1H), 2.53 (t, J = 7.6 Hz, 2H), 2.44 (t, J = 7.6 Hz, 2H), 1.56-1.42 (m, 4H), 1.30-1.16 (m, 4H), 0.90-0.86 (m, 6H).

^{13}C NMR (101 MHz, $CDCl_3$) 154.1, 146.6, 142.7, 141.4, 141.3, 140.2, 140.1, 139.5, 137.0, 136.3, 134.7, 131.45, 131.39, 131.3, 130.3, 129.1, 127.8, 127.6, 127.3, 119.5, 117.4, 114.7, 105.8, 35.4, 35.2, 33.8, 33.5, 22.2, 22.1, 14.12, 14.10.

^{11}B NMR ($CDCl_3$, 128 MHz): δ (ppm) 31.9.

HRMS: Calcd. For: $C_{42}H_{42}BN_2O^+$ ($M+H^+$): 601.3385; found 601.3390.

IR: 762, 819, 1064, 1110, 1171, 1259, 1327, 1373, 1408, 1516, 1599, 1628, 2358, 2857, 2928, 2955, 3420, 3583 cm^{-1}.

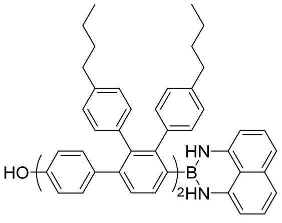

BOC1-2

General procedure B was used for the triflation step (92% yield). General procedure B was used for the SMC step. **BOC1-2** was obtained in 88% yield over two steps (0.7 mmol scale, 626.3 mg) as a white solid.

R_f = 0.3 (Hexanes/EA = 4:1)

^{1}H NMR ($CDCl_3$, 400 MHz): δ (ppm) 7.68 (d, J = 8.0 Hz, 1H), 7.46-7.41 (m, 3H), 7.03-6.87 (m, 14H), 6.75-6.59 (m, 14H), 6.01 (dd, J_1 = 7.2 Hz, J_2 = 1.2 Hz, 2H), 5.36 (s,

2H), 4.54 (s, 1H), 2.54-2.40 (m, 8H), 1.53-1.41 (m, 8H), 1.29-1.15 (m, 8H), 0.89-0.85 (m, 12H).

^{11}B NMR (CDCl$_3$, 128 MHz): δ (ppm) 31.2.

HRMS: Calcd. For: $C_{58}H_{41}BN_2O^+$ (M^+): 1016.5816; found 1016.5812.

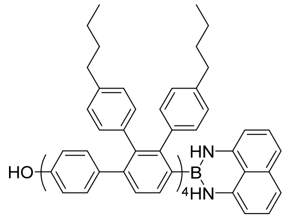

BOC1-4

General procedure B was used for the triflation step (95% yield). General procedure B was used for the SMC step. **BOC1-4** was obtained in 87% yield over two steps (0.43 mmol scale, 692.1 mg) as a white solid.

R_f = 0.25 (Hexanes/EA = 4:1)

^{1}H NMR (CDCl$_3$, 400 MHz): δ (ppm) 7.68 (d, J = 8.0 Hz, 1H), 7.47-7.40 (m, 7H), 7.04-6.94 (m, 11H), 6.88-6.85 (m, 11H), 6.76-6.59 (m, 29H), 6.01 (d, J = 7.2 Hz, 2H), 5.37 (s, 2H), 4.53 (s, 1H), 2.54-2.41 (m, 16H), 1.54-1.41 (m, 16H), 1.27-1.18 (m, 16H), 0.90-0.85 (m, 24H).

^{11}B NMR (CDCl$_3$, 128 MHz): δ (ppm) 29.3.

HRMS: Calcd. For: $C_{138}H_{137}BN_2O^+$ (M^+): 1850.0858; found 1850.0757.

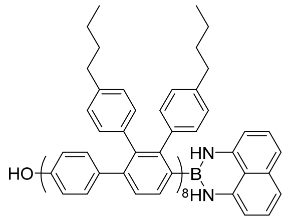

BOC1-8

General procedure A was used for the triflation step (90% yield). General procedure B was used for the SMC step. **BOC1-8** was obtained in 85% yield over two steps (0.21 mmol scale, 627.6 mg) as a white solid.

R_f = 0.2 (Hexanes/DCM = 1:1)

^{1}H NMR (CDCl$_3$, 400 MHz): δ (ppm) 7.68 (d, J = 7.6 Hz, 1H), 7.49-7.40 (m, 15H), 7.04-6.85 (m, 40H), 6.76-6.59 (m, 60H), 6.01 (d, J = 7.2 Hz, 2H), 5.37 (s, 2H), 4.53 (s, 1H), 2.56-2.41 (m, 32H), 1.53-1.44 (m, 32H), 1.27-1.17 (m, 32H), 0.90-0.86 (m, 48H).

^{11}B NMR (CDCl$_3$, 128 MHz): δ (ppm) 30.2.

MALDI-TOF-MS: Calcd. For: $C_{266}H_{265}BN_2O^+$ (M^+): 3516.09; found 3515.40.

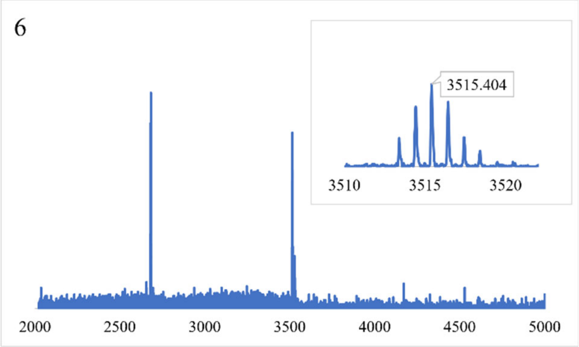

Figure S8. MALDI-TOF-MS spectrum of **BOS1-8**

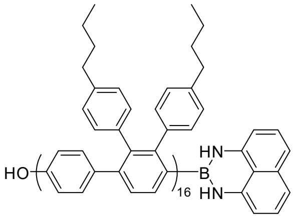

BOC1-16

General procedure A was used for the triflation step (88% yield). General procedure B was used for the SMC step. **BOC1-16** was obtained in 86% yield over two steps (0.084 mmol scale, 575.1 mg) as a white solid.

R_f = 0.2 (Hexanes/DCM = 1:1)

^{1}H NMR ($CDCl_3$, 400 MHz): δ (ppm) 7.69 (d, J = 7.6 Hz, 1H), 7.47-7.41 (m, 31H), 7.04-6.60 (m, 196H), 6.02 (d, J = 6.8 Hz, 2H), 5.37 (s, 2H), 4.60 (s, 1H), 2.55-2.45 (m, 64H), 1.53-1.45 (m, 64H), 1.28-1.19 (m, 64H), 0.90-0.87 (m, 96H).

^{11}B NMR ($CDCl_3$, 128 MHz): δ (ppm) 30.4.

MALDI-TOF-MS: Calcd. For: $C_{522}H_{521}BN_2O^+$ (M^+): 6847.10; found 6847.02.

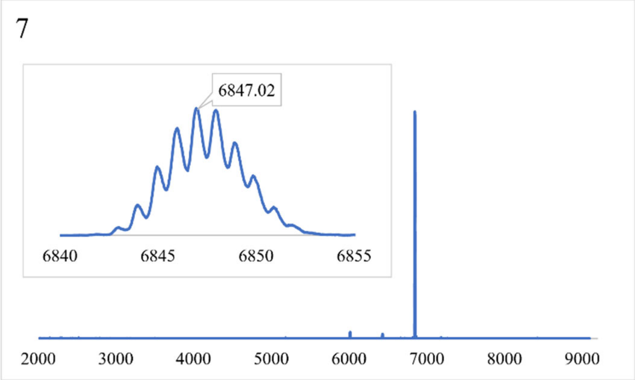

Figure S9. MALDI-TOF-MS spectrum of **BOS1-16**

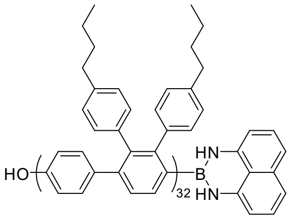

BOC1-32

General procedure A was used for the triflation step (87% yield). General procedure B was used for the SMC step. **BOC1-32** was obtained in more than 70% yield over two steps (0.028 mmol scale, 295.2 mg) as a white solid. The NMR can't be recorded because of its poor solubility in normal organic solvents.

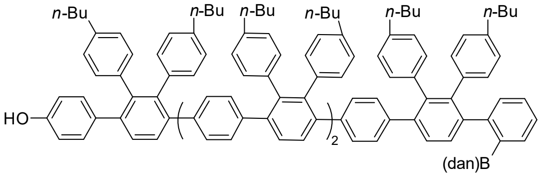

BOS2-9

General procedure A was used for the SMC. **BOS2-9** was obtained in 86% yield (0.292 mmol scale, 483.7 mg) as a white solid.

R_f = 0.3 (Hexanes/DCM = 1:1)

^{1}H NMR ($CDCl_3$, 400 MHz): δ (ppm) 7.52-7.35 (m, 11H), 7.25-7.21 (m, 1H), 7.06-7.02 (m, 2H), 6.98-6.85 (m, 16H), 6.73-6.39 (m, 34H), 6.02 (d, J = 7.2 Hz, 2H), 5.22 (s, 2H), 4.53 (s, 1H).2.49-2.26 (m, 16H), 1.51-1.34 (m, 16H), 1.24-1.10 (m, 16H), 0.90-0.81 (m, 24H).

MALDI-TOF-MS: Calcd. For: $C_{144}H_{141}BN_2O^+$ (M^+): 1926.12; found 1926.66.

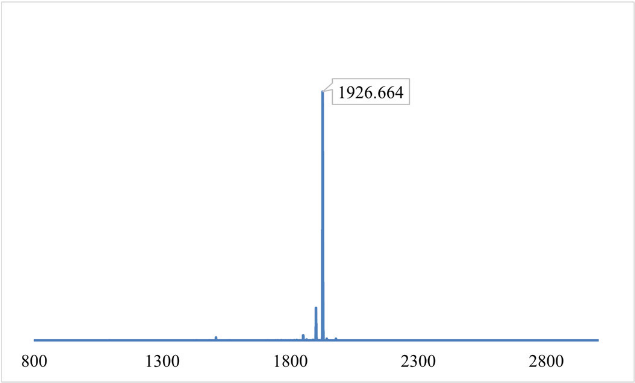

Figure S10. MALDI-TOF-MS spectrum of **BOS2-9**

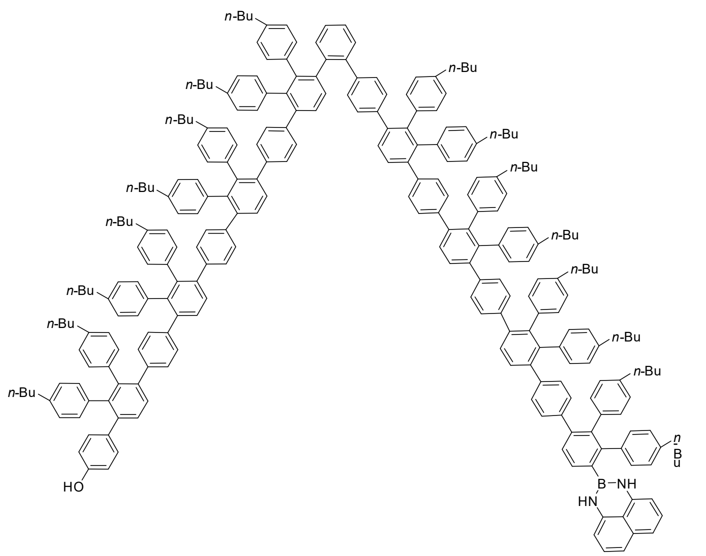

BOS2-17

General procedure B was used for the triflation step (93% yield). General procedure B was used for the SMC step. **BOS2-17** was obtained in 85% yield over two steps (0.241 mmol scale, 736.0 mg) as a white solid.

R_f = 0.25 (Hexanes/DCM = 1:1)

^{1}H NMR ($CDCl_3$, 400 MHz): δ (ppm) 7.68 (d, J = 8.0 Hz, 1H), 7.46-7.32 (m, 16H), 7.23-7.17 (m, 2H), 7.05-6.85 (m, 39H), 6.76-6.52 (m, 62H), 6.01 (d, J = 6.8 Hz, 2H), 5.36 (s, 2H), 4.52 (s, 1H), 2.54-2.28 (m, 32H), 1.51-1.45 (m, 32H), 1.23-1.11 (m, 32H), 0.89-0.82 (m, 48H).

MALDI-TOF-MS: Calcd. For: $C_{272}H_{270}BN_2O^+$ (M+H$^+$): 3593.13; found 3593.37.

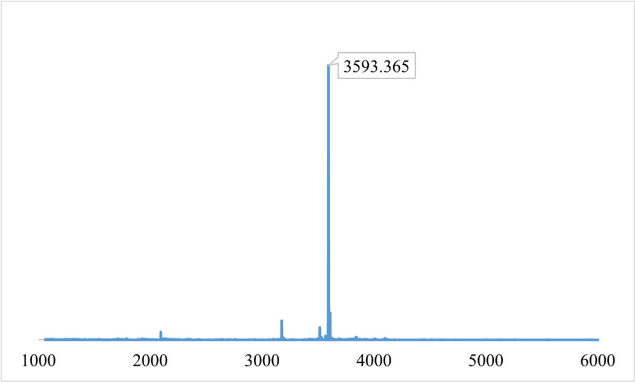

Figure S11. MALDI-TOF-MS spectrum of **BOS2-17**

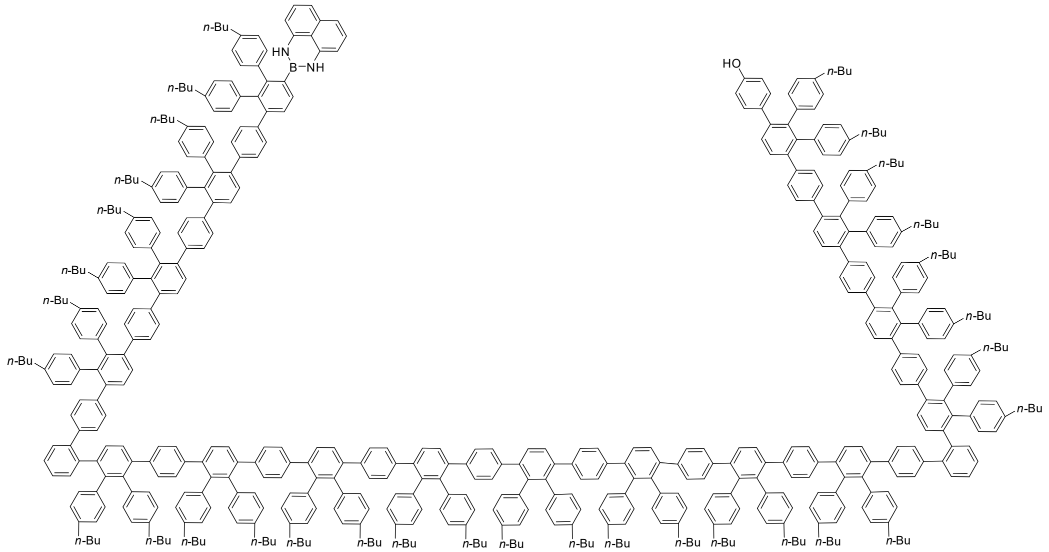

BOS2-34

General procedure B was used for the triflation step (95% yield). General procedure B was used for the SMC step. **BOS2-34** was obtained in 86% yield over two steps (511.7 mg) as a white solid.

R_f = 0.2 (Hexanes/DCM = 1:1)

^{1}H NMR ($CDCl_3$, 400 MHz): δ (ppm) 7.69 (d, J = 7.6 Hz, 1H), 7.47-7.32 (m, 32H), 7.23-7.17 (m, 4H), 7.05-6.48 (m, 195H), 6.02-6.01 (m, 4H), 5.55-5.53 (m, 2H), 5.37 (s, 2H), 4.52 (s, 1H), 2.54-2.26 (m, 64H), 1.51-1.33 (m, 64H), 1.26-1.12 (m, 64H), 0.90-0.82 (m, 96H).

MALDI-TOF-MS: Calcd. For: $C_{534}H_{530}BN_2O^+$ (M+H$^+$): 7000.17; found 7001.18.

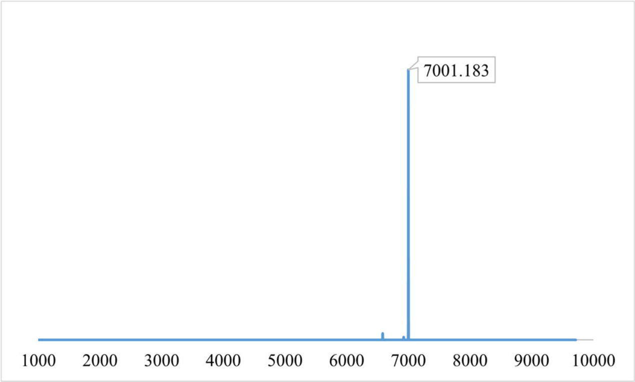

Figure S12. MALDI-TOF-MS spectrum of **BOS2-34**

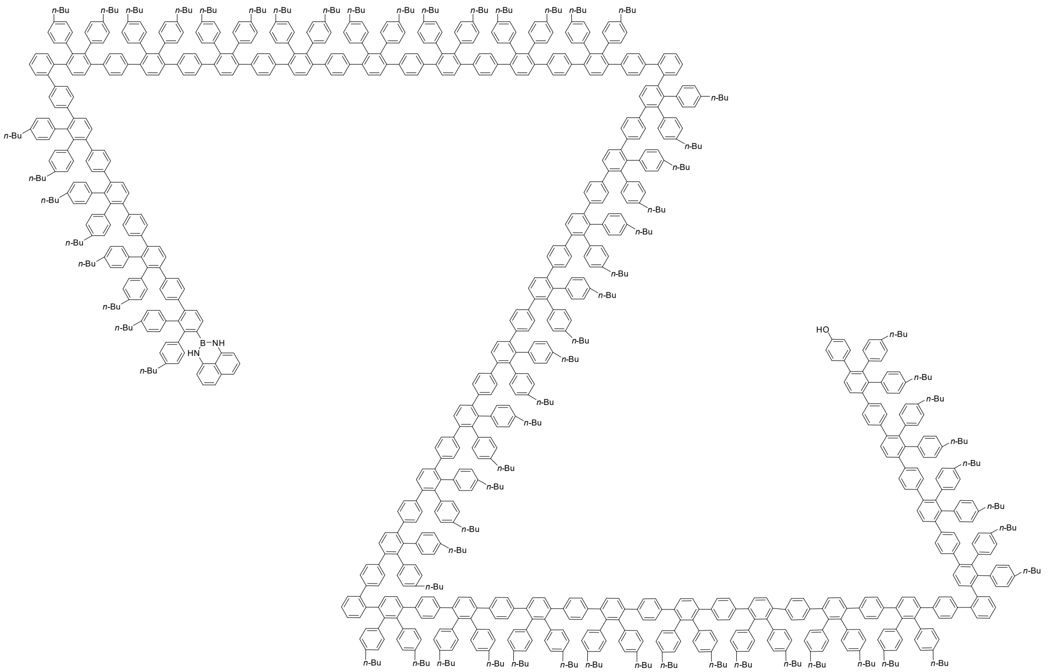

BOS2-68

General procedure B was used for the triflation step (92% yield). General procedure B was used for the SMC step. **BOS2-68** was obtained in 83% yield over two steps (0.036 mmol scale, 412.8 mg) as a white solid.

R_f = 0.2 (Hexanes/DCM = 1:1)

^{1}H NMR (CDCl$_3$, 400 MHz): δ (ppm) 7.69 (d, J = 8.0 Hz, 1H), 7.47-7.33 (m, 64H), 7.23-7.17 (m, 8H), 7.05-6.59 (m, 389H), 6.02-6.01 (m, 6H), 5.55-5.51 (m, 4H), 5.37 (s, 2H), 4.52 (s, 1H), 2.54-2.26 (m, 128H), 1.51-1.33 (m, 128H), 1.26-1.11 (m, 128H), 0.90-0.82 (m, 192H).

MALDI-TOF-MS: Calcd. For: $C_{1058}H_{1049}BN_2O^+$ (M^+): 13813.94; found 13821.49.

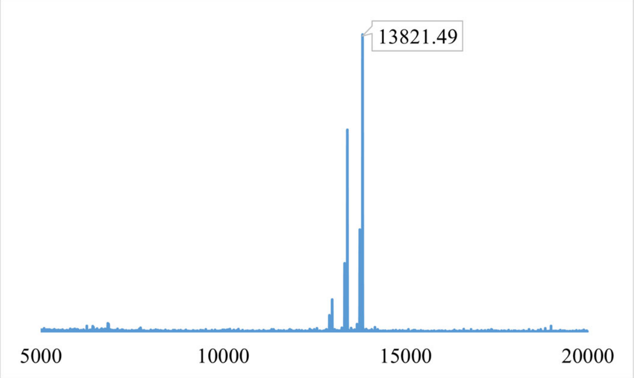

Figure S13. MALDI-TOF-MS spectrum of **BOS2-68**

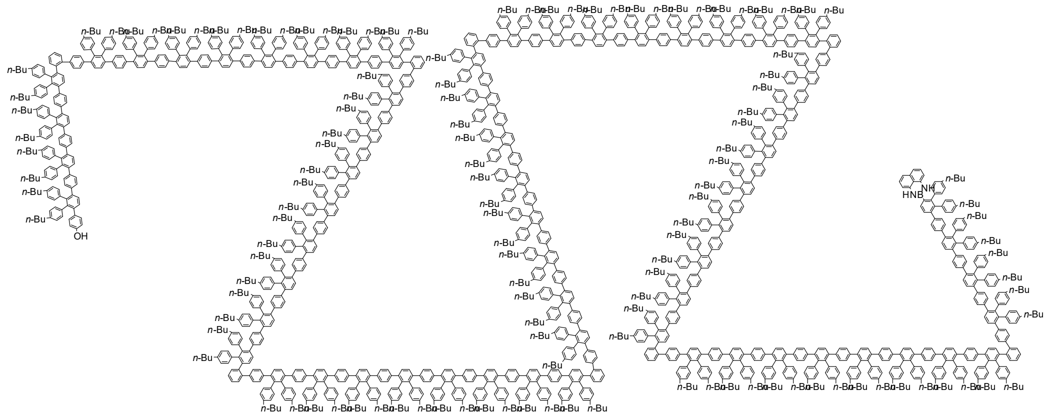

BOS2-136

General procedure B was used for the triflation step (96% yield). General procedure B was used for the SMC step. **BOS2-136** was obtained in 86% yield over two steps (0.0037 mmol scale, 87.2 mg) as a white solid.

R_f = 0.2 (Hexanes/DCM = 1:1.2)

^{1}H NMR ($CDCl_3$, 400 MHz): δ (ppm) 7.68 (d, J = 9.6 Hz, 1H), 7.52-7.32 (m, 131H), 7.23-7.16 (m, 16H), 7.05-6.39 (m, 766H), 6.02-5.97 (m, 11H), 5.55-5.52 (m, 9H), 5.36 (s, 2H), 4.53 (s, 1H), 2.54-2.26 (m, 256H), 1.51-1.33 (m, 256H), 1.25-1.11 (m, 256H), 0.89-0.82 (m, 384H).

MALDI-TOF-MS: Calcd. For: $C_{2106}H_{2089}BN_2O^+$ (M^+): 27451.0; found 27374.

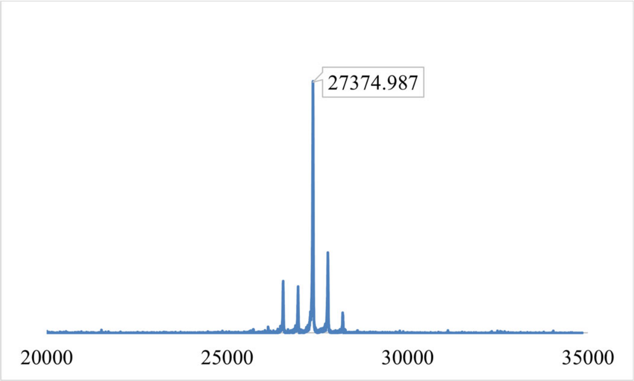

Figure S14. MALDI-TOF-MS spectrum of **BOS2-136**

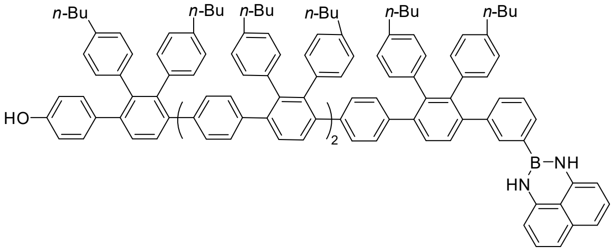

BOS3-9

General procedure A was used for the SMC. **BOS3-9** was obtained in 82% yield (0.68 mmol scale, 1.08 g) as a white solid.

R_f = 0.3 (Hexanes/DCM = 1:1)

^{1}H NMR ($CDCl_3$, 400 MHz): δ (ppm) 7.55-7.48 (m, 2H), 7.43-7.30 (m, 9H), 7.22 (s, 1H), 7.13-7.10 (m, 2H), 7.03-6.96 (m, 12H), 6.83-6.81 (m, 2H), 6.77-6.72 (m, 16H)，6.67-6.60 (m, 16H), 6.32 (d, J = 7.2 Hz, 2H), 5.68 (s, 2H), 4.56 (s, 1H).2.48-2.41 (m, 16H), 1.50-1.42 (m, 16H), 1.27-1.13 (m, 16H), 0.90-0.86 (m, 21H), 0.78 (t, J = 7.2 Hz, 3H).

MALDI-TOF-MS: Calcd. For: $C_{144}H_{141}BN_2O^+$ (M^+): 1926.12; found 1927.26.

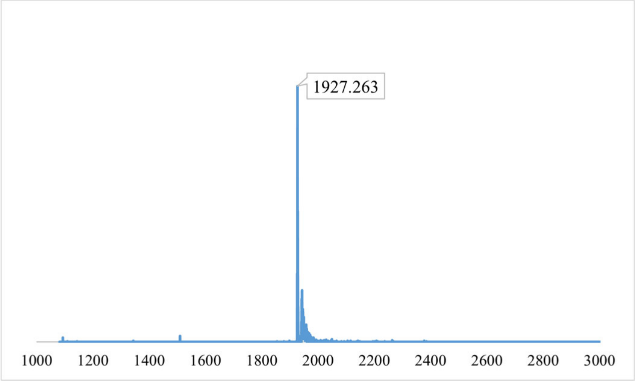

Figure S15. MALDI-TOF-MS spectrum of **BOS3-9**

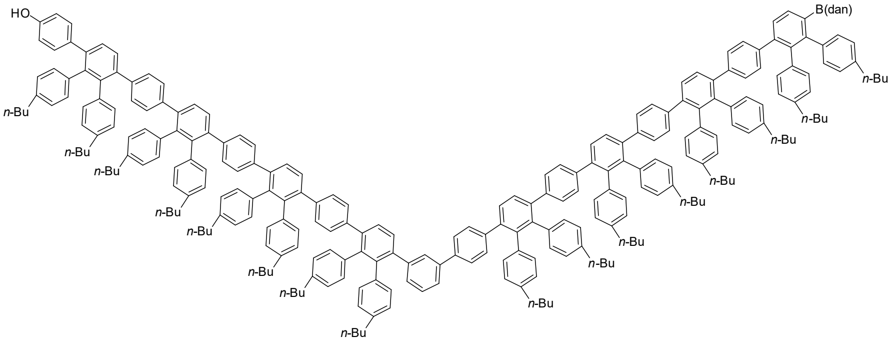

BOS3-17

General procedure B was used for the triflation step (93% yield). General procedure A was used for the SMC step. **BOS3-17** was obtained in 82% yield over two steps (0.46 mmol scale, 1.35 g) as a white solid.

R_f = 0.25 (Hexanes/DCM = 1:1)

^{1}H NMR ($CDCl_3$, 400 MHz): δ (ppm) 7.69 (d, J = 7.6 Hz, 1H), 7.51-7.41 (m, 16H), 7.30 (d, J = 7.2 Hz, 1H), 7.21 (t, J = 8.0 Hz, 1H), 7.13-6.94 (m, 16H), 6.88-6.86 (m, 23H), 6.77-6.60 (m, 62H), 6.02 (d, J = 6.8 Hz, 2H), 5.37 (s, 2H), 4.55 (s, 1H), 2.55-2.41 (m, 32H), 1.52-1.44 (m, 32H), 1.28-1.19 (m, 32H), 0.90-0.83 (m, 48H).

MALDI-TOF-MS: Calcd. For: $C_{272}H_{270}BN_2O^+$ (M+H$^+$): 3593.13; found 3594.97.

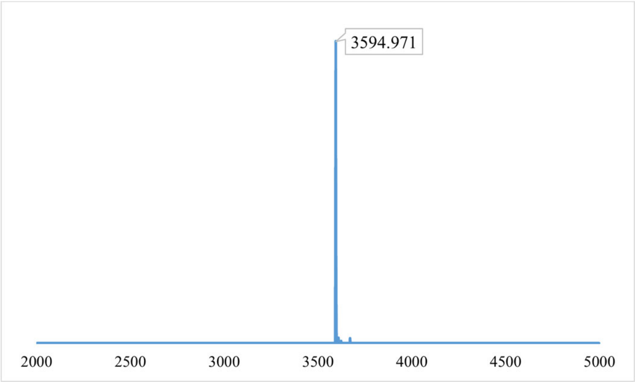

Figure S16. MALDI-TOF-MS spectrum of **BOS3-17**

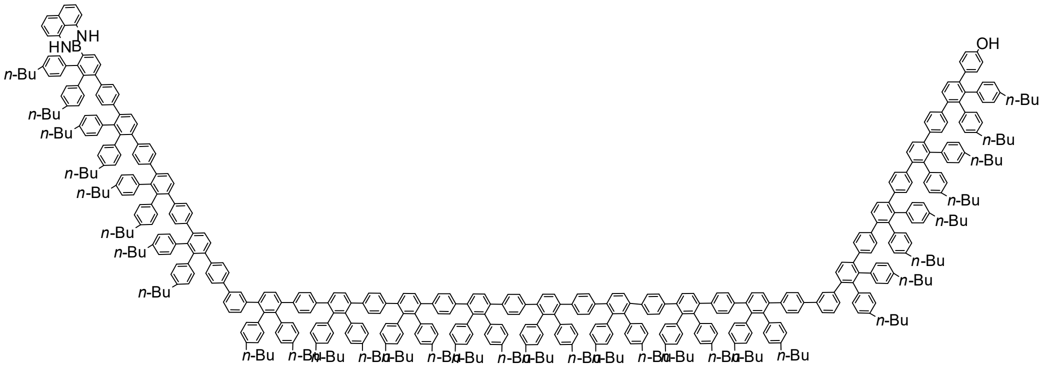

BOS3-34

General procedure B was used for the triflation step (90% yield). General procedure B was used for the SMC step. **BOS3-34** was obtained in 87% yield over two steps (0.12 mmol, 730.8 mg) as a white solid.

R_f = 0.2 (Hexanes/DCM = 1:1)

^{1}H NMR ($CDCl_3$, 400 MHz): δ (ppm) 7.69 (d, J = 7.6 Hz, 1H), 7.50-7.41 (m, 30H), 7.30 (d, J = 6.8 Hz, 2H), 7.21 (t, J = 7.2 Hz, 2H), 7.13-6.94 (m, 21H), 6.87-6.86 (m, 50H), 6.76-6.71 (m, 68H), 6.67-6.59 (m, 62H), 6.01 (d, J = 6.8 Hz, 2H), 5.37 (s, 2H), 4.56 (s, 1H), 2.54-2.41 (m, 64H), 1.52-1.44 (m, 64H), 1.26-1.16 (m, 64H), 0.90-0.82 (m, 96H).

MALDI-TOF-MS: Calcd. For: $C_{534}H_{530}BN_2O^+$ (M+H$^+$): 7000.17; found 6986.18.

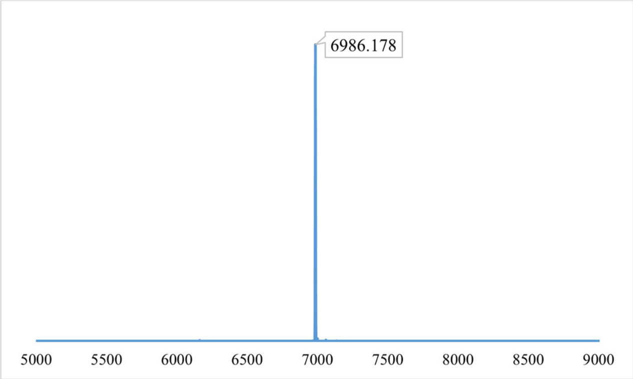

Figure S17. MALDI-TOF-MS spectrum of **BOS3-34**

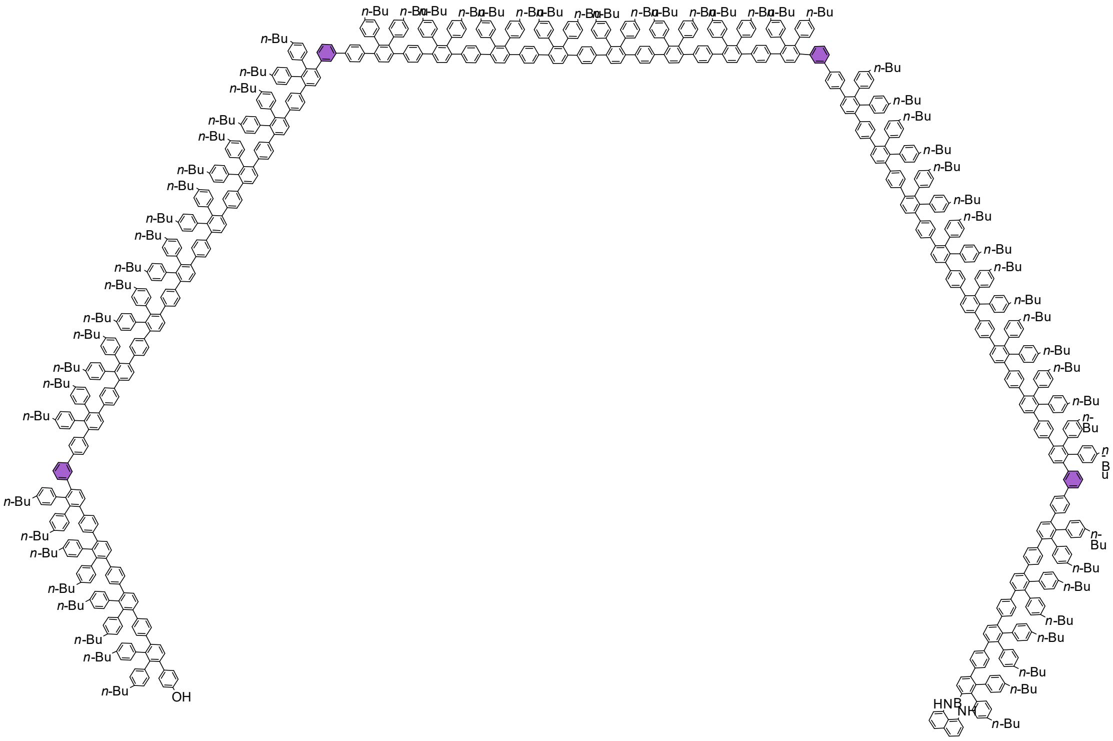

BOS3-68

General procedure B was used for the triflation step (95% yield). General procedure B was used for the SMC step. **BOS3-68** was obtained in more than 70% yield over two steps (0.057 mmol scale, 567.0 mg) as a white solid. The NMR can’t be recorded because of its poor solubility in normal organic solvents.

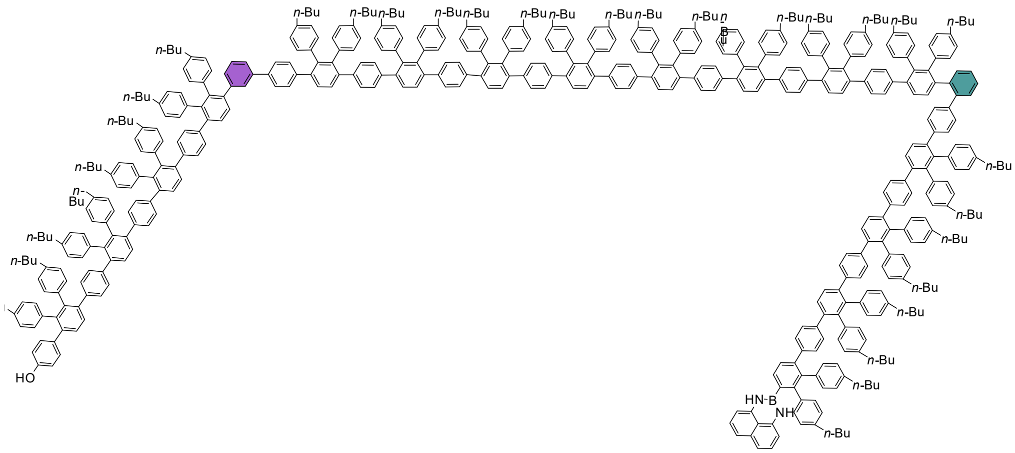

BOS4-34

General procedure B was used for the triflation step (90% yield). General procedure B was used for the SMC step. **BOS3-34** was obtained in 85% yield over two steps (0.12 mmol, 714.2 mg) as a white solid.

R_f = 0.2 (Hexanes/DCM = 1:1)

^{1}H NMR ($CDCl_3$, 400 MHz): δ (ppm) 7.69 (d, J = 8.0 Hz, 1H), 7.52-7.29 (m, 34H), 7.21-6.47 (m, 200H), 6.02 (d, J = 7.2 Hz, 2H), 5.54 (s, 1H), 5.37 (s, 2H), 4.53 (s, 1H), 2.55-2.27 (m, 64H), 1.53-1.34 (m, 64H), 1.28-1.12 (m, 64H), 0.90-0.75 (m, 96H).

MALDI-TOF-MS: Calcd. For: $C_{534}H_{530}BN_2O^+$ (M+H$^+$): 7000.17; found 7008.84.

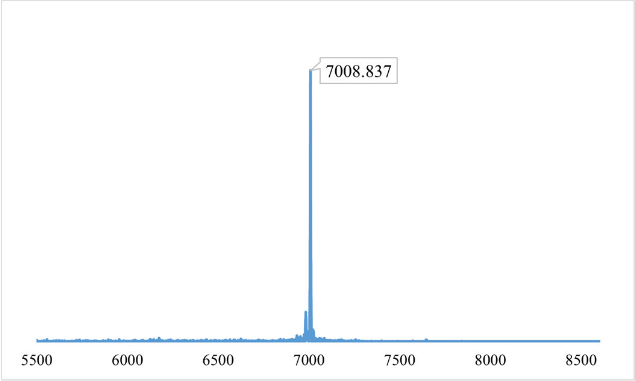

Figure S18. MALDI-TOF-MS spectrum of **BOS4-34**

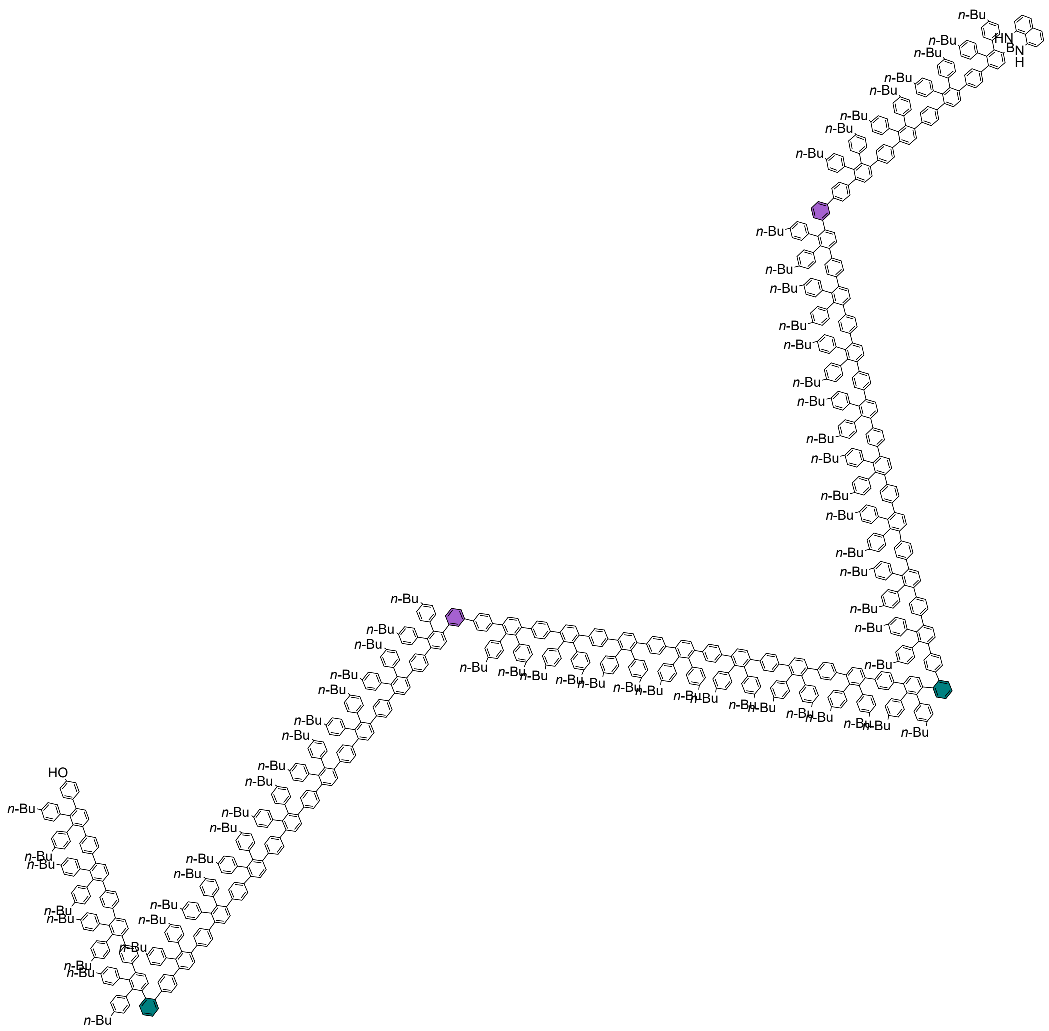

BOS4-68

General procedure B was used for the triflation step (92% yield). General procedure B was used for the SMC step. **BOS4-68** was obtained in 87% yield over two steps (0.048 mmol scale, 578.5 mg) as a white solid.

R_f = 0.2 (Hexanes/DCM = 1:1)

^{1}H NMR ($CDCl_3$, 400 MHz): δ (ppm) 7.69 (d, J = 8.0 Hz, 1H), 7.52-7.29 (m, 68H), 7.21-6.86 (m, 140H), 6.74-6.51 (m, 255H), 6.02 (d, J = 7.2 Hz, 2H), 5.55 (s, 2H), 5.37 (s, 2H), 4.51 (s, 1H), 2.55-2.27 (m, 128H), 1.52-1.40 (m, 128H), 1.26-1.12 (m, 128H), 0.90-0.83 (m, 192H).

MALDI-TOF-MS: Calcd. For: $C_{1058}H_{1049}BN_2O^+$ (M^+): 13813.94; found 13827.86.

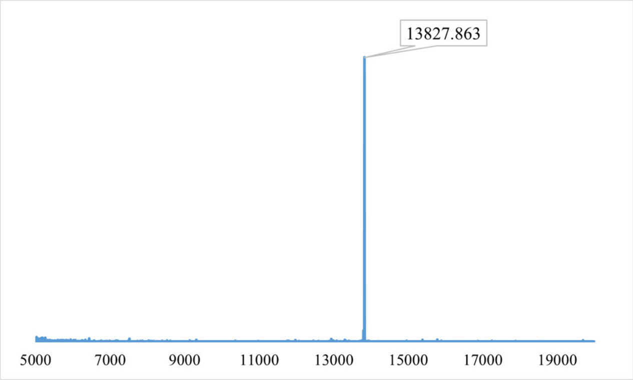

Figure S19. MALDI-TOF-MS spectrum of **BOS4-68**

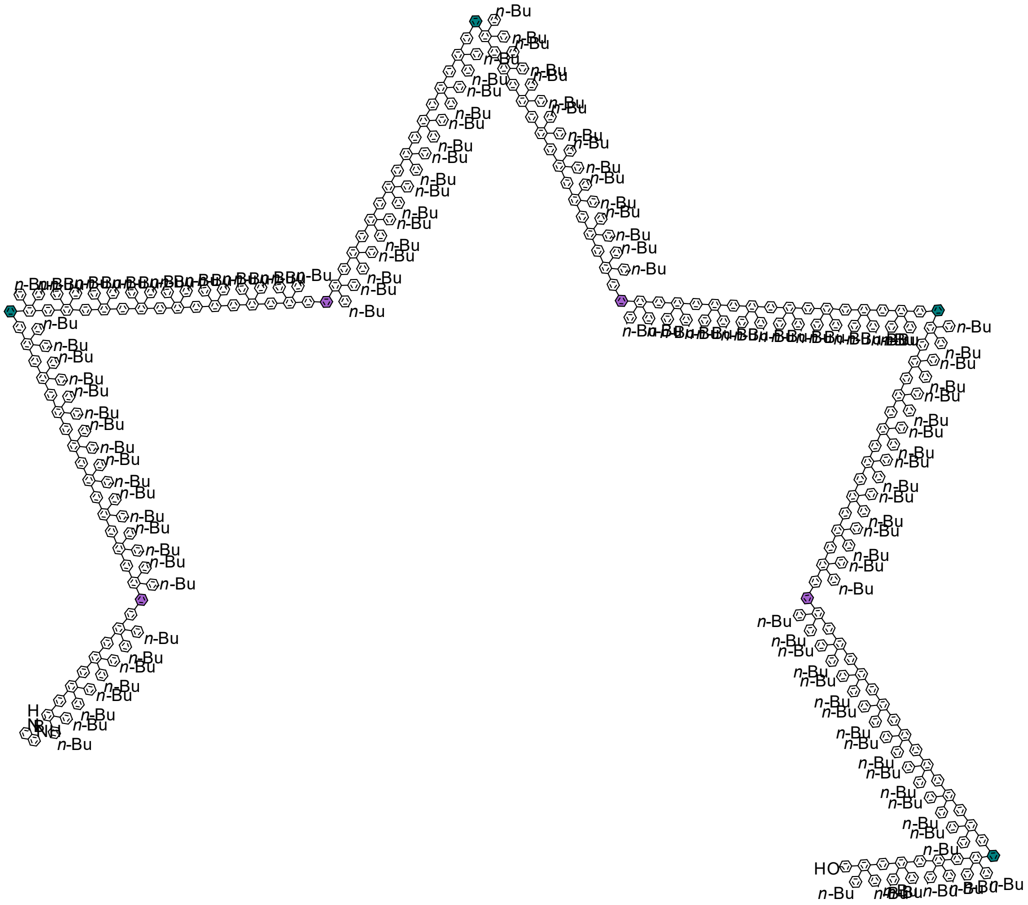

BOS4-136

General procedure B was used for the triflation step (93% yield). General procedure B was used for the SMC step. **BOS2-136** was obtained in 82% yield over two steps (0.012 mmol scale, 270.1 mg) as a white solid.

R_f = 0.2 (Hexanes/DCM = 1:1.2)

^{1}H NMR ($CDCl_3$, 400 MHz): δ (ppm) 7.68 (d, J = 7.6 Hz, 1H), 7.45-7.32 (m, 133H), 7.21-7.02 (m, 40H), 6.96-6.59 (m, 748H), 6.01 (d, J = 6.8 Hz, 2H), 5.55-5.53 (m, 6H),

5.37 (s, 2H), 4.57 (s, 1H), 2.46-2.29 (m, 256H), 1.49-1.46 (m, 256H), 1.26-1.16 (m, 256H), 0.90-0.82 (m, 384H).

MALDI-TOF-MS: Calcd. For: $C_{2106}H_{2089}BN_2O^+$ (M^+): 27451.0; found 27325.

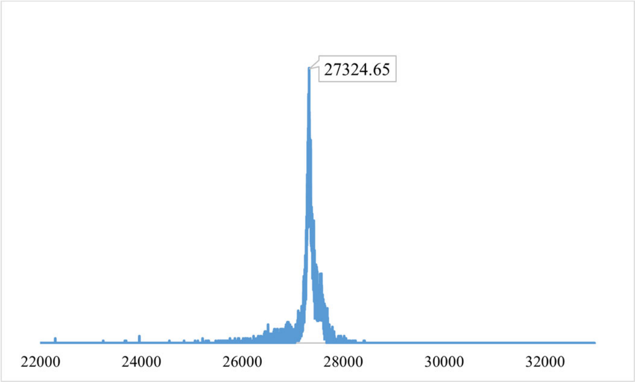

Figure S20. MALDI-TOF-MS spectrum of **BOS4-136**

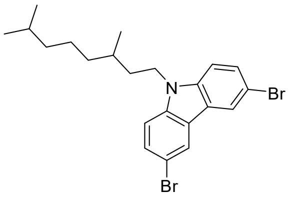

BBB$_{\mathbf{carbazole}}$-S1

By following the reported procedure,[4] **BBB$_{\mathbf{carbazole}}$-S1** was obtained in 83% yield (13.2 g) as a colorless gel.

R_f = 0.3 (Hexanes/DCM = 4:1)

^{1}H NMR ($CDCl_3$, 400 MHz): δ (ppm) 8.08 (d, J = 1.6 Hz, 2H), 7.54-7.52 (m, 2H), 7.21 (d, J = 8.8 Hz, 2H), 4.24-4.11 (m, 2H), 1.83-1.74 (m, 1H), 1.62-1.46 (m, 3H), 1.37-1.28 (m, 2H), 1.24-1.09 (m, 4H), 1.02-1.00 (m, 3H), 0.88-0.85 (m, 6H).

^{13}C NMR ($CDCl_3$, 101 MHz) 139.2, 129.1, 123.5, 123.3, 112.0, 110.3, 41.5, 39.3, 37.1, 35.5, 30.9, 28.1, 24.7, 22.8, 22.7, 19.8.

HRMS: Calcd. For: $C_{22}H_{27}Br_2N^+$ (M^+): 465.0490; found 465.0492.

IR: 564, 635, 648, 796, 832, 866, 1019, 1058, 1147, 1212, 1285, 1318, 1349, 1378, 1437, 1471, 1592, 1722, 1851, 2867, 2926, 2954, 3058 cm^{-1}.

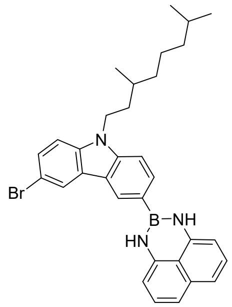

BBB$_{carbazole}$

Following the general procedure for the synthesis of bifunctional building block, **BBB$_{carbazole}$** was obtained in 79% yield (8.7 g) as a grey solid.

R_f = 0.3 (Hexanes/DCM = 3:1)

^{1}H NMR ($CDCl_3$, 400 MHz): δ (ppm) 8.27-8.26 (m, 2H), 7.69 (dd, J_1 = 8.0 Hz, J_2 = 0.8 Hz, 1H), 7.55 (dd, J_1 = 8.8 Hz, J_2 = 2.0 Hz, 1H), 7.40 (d, J = 8.0 Hz, 1H), 7.26-7.24 (m, 1H), 7.18-7.14 (m, 2H), 7.08-7.06 (m, 2H), 6.44 (d, J = 6.8 Hz, 2H), 6.08 (s, 2H), 4.29-4.17 (m, 2H), 1.87-1.78 (m, 1H), 1.66-1.56 (m, 1H), 1.54-1.46 (m, 2H), 1.38-1.28 (m, 2H), 1.25-1.09 (m, 4H), 1.02 (d, J = 6.8 Hz, 3H), 0.87-0.85 (m, 6H).

^{13}C NMR ($CDCl_3$, 101 MHz) 142.1, 141.4, 139.2, 136.5, 129.5, 128.6, 127.8, 124.7, 124.2, 123.3, 122.1, 119.8, 117.8, 112.2, 110.3, 108.8, 106.1, 41.5, 39.3, 37.2, 35.6, 31.0, 28.1, 24.7, 22.8, 22.7, 19.9.

^{11}B NMR ($CDCl_3$, 128 MHz): δ (ppm) 30.8.

HRMS: Calcd. For: $C_{32}H_{36}BBr_2N_3^+$ (M + H^+): 552.2181; found 552.2185.

IR: 660, 762, 802, 818, 1084, 1208, 1237, 1332, 1373, 1408, 1476, 1515, 1598, 1627, 2341, 2360, 2866, 2925, 2952, 3052, 3420 cm^{-1}.

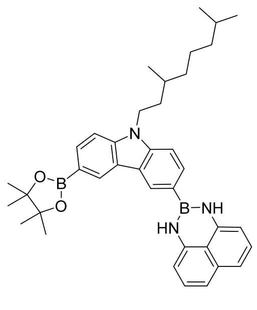

BBB$_{carbazole}$-2

Following the general procedure for the borylation of bifunctional building block, **BBB$_{carbazole}$-2** was obtained in 88% yield (6.3 g) as a grey solid.

R_f = 0.3 (Hexanes/DCM = 3:1)

^{1}H NMR ($CDCl_3$, 400 MHz): δ (ppm) 8.69 (s, 1H), 8.48 (s, 1H), 7.96 (dd, J_1 = 7.6 Hz,

J_2 = 0.8 Hz, 1H), 7.73 (d, J = 8.4 Hz, 1H), 7.47-7.41 (m, 2H), 7.20-7.16 (m, 2H), 7.09-7.07 (m, 2H), 6.49 (d, J = 7.2 Hz, 2H), 6.17 (s, 2H), 4.41-4.28 (m, 2H), 1.92-1.84 (m, 1H), 1.71-1.62 (m, 1H), 1.54-1.48 (m, 1H), 1.42 (s, 12H), 1.39-1.19 (m, 5H), 1.16-1.10 (m, 2H), 1.05-1.03 (m, 3H), 0.87-0.85 (m, 6H).

^{13}C NMR ($CDCl_3$, 101 MHz) 142.8, 142.0, 141.6, 136.5, 132.5, 128.9, 128.0, 127.8, 124.2, 123.4, 122.7, 119.9, 117.7, 108.7, 108.3, 106.1, 83.8, 41.5, 39.4, 37.3, 35.7, 31.0, 28.1, 25.1, 24.7, 22.8, 22.7, 19.9.

^{11}B NMR ($CDCl_3$, 128 MHz): δ (ppm) 32.1.

HRMS: Calcd. For: $C_{38}H_{48}B_2N_3O_2^+$ (M+H$^+$): 600.3928; found 600.3927.

IR: 638, 661, 764, 819, 856, 891, 963, 1084, 1144, 1353, 1408, 1481, 1518, 1599, 1627, 2868, 2926, 2954, 3052, 3421 cm^{-1}.

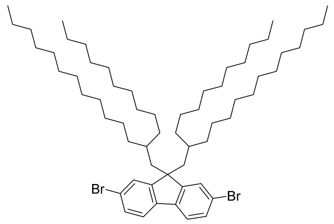

BBB$_{\text{fluorene}}$-S1

By following the reported procedure,[5, 6] the known compound **BBB$_{\text{fluorene}}$-S1**was obtained in 80% yield (23.9 g) as a colorless gel.

R_f = 0.4 (Hexanes/DCM = 5:1)

^{1}H NMR ($CDCl_3$, 400 MHz): δ (ppm) 7.52-7.43 (m, 6H), 1.93 (d, J = 5.2 Hz, 4H), 1.31-1.15 (m, 45H), 1.10-1.09 (m, 7H), 0.90-0.87 (m, 30H), 0.72-0.70 (m, 8H), 0.52-0.48 (m, 2H).

^{13}C NMR ($CDCl_3$, 101 MHz) δ (ppm) 152.5, 139.4, 130.2, 127.7, 121.14, 121.10, 55.6, 44.9, 34.9, 33.4, 32.1, 29.88, 29.85, 29.83, 29.78, 29.73, 29.5, 26.1, 22.9, 14.3.

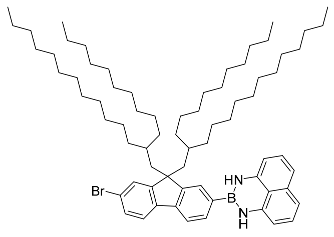

BBB$_{\text{fluorene}}$

Following the general procedure for the synthesis of bifunctional building block,

BBB$_{fluorene}$ was obtained in 78% yield (8.5 g) as a colorless gel.

R_f = 0.3 (Hexanes/DCM = 4:1)

^{1}H NMR ($CDCl_3$, 400 MHz): δ (ppm) 7.72 (d, J = 8.0 Hz, 1H), 7.62-7.53 (m, 4H), 7.46 (dd, J_1 = 8.0 Hz, J_2 = 1.6 Hz, 1H), 7.17-7.13 (m, 2H), 7.07-7.05 (m, 2H), 6.44 (d, J = 7.2 Hz, 2H), 6.01 (s, 2H), 2.08-1.96 (m, 4H), 1.33-1.05 (m, 58H), 0.91-0.82 (m, 26H), 0.76-0.65 (m, 8H), 0.54-0.48 (m, 2H).

^{13}C NMR ($CDCl_3$, 101 MHz) δ (ppm) 153.2, 150.0, 142.6, 141.2, 140.1, 136.6, 130.4, 130.1, 127.8, 127.7, 127.0, 121.4, 121.2, 120.0, 119.7, 118.0, 106.2, 55.4, 44.9, 35.0, 34.9, 33.4, 32.11, 32.08, 32.04, 29.94, 29.88, 29.86, 29.79, 29.75, 29.54, 29.51, 26.3, 26.2, 22.87, 22.85, 22.81, 14.29, 14.26.

^{11}B NMR ($CDCl_3$, 128 MHz): δ (ppm) 31.8.

HRMS: Calcd. For: $C_{71}H_{113}BBrN_2^+$ (M+H$^+$): 1083.8175; found 1083.8141.

IR: 720, 760, 818, 1083, 1165, 1236, 1325, 1375, 1407, 1458, 1508, 1588, 1602, 1628, 2852, 2923, 3440 cm^{-1}.

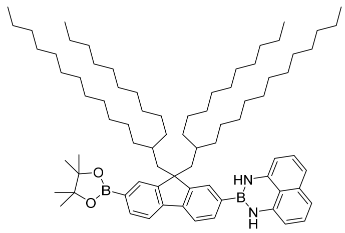

BBB$_{fluorene}$-2

Following the general procedure for the borylation of bifunctional building block, **BBB$_{fluorene}$-2** was obtained in 75% yield (5.9 g) as a colorless gel.

R_f = 0.2 (Hexanes/DCM = 3:1)

^{1}H NMR ($CDCl_3$, 400 MHz): δ (ppm) 7.85 (s, 1H), 7.81-7.76 (m, 2H), 7.73-7.71 (m, 1H), 7.63-7.60 (m, 2H), 7.17-7.13 (m, 2H), 7.07-7.05 (m, 2H), 6.45 (d, J = 7.2 Hz, 2H), 6.03 (s, 2H), 2.10-2.00 (m, 4H), 1.37 (s, 12H), 1.30-1.04 (m, 57H), 0.90-0.66 (m, 37H), 0.52-0.46 (m, 2H).

^{13}C NMR ($CDCl_3$, 101 MHz) δ (ppm) 150.9, 150.0, 144.0, 143.6, 141.3, 136.6, 133.8, 130.7, 130.1, 128.5, 127.7, 127.1, 120.03, 120.00, 119.3, 117.9, 106.1, 83.7, 55.1, 44.8, 35.1, 35.0, 33.5, 32.1, 32.0, 29.99, 29.96, 29.92, 29.90, 29.87, 29.83, 29.81, 29.76, 29.72, 29.55, 29.53, 29.51, 26.3, 26.1, 25.0, 22.9, 22.8, 14.28, 14.26.

^{11}B NMR ($CDCl_3$, 128 MHz): δ (ppm) 31.7.

HRMS: Calcd. For: $C_{77}H_{125}B_2N_2O_2^+$ (M+H$^+$): 1131.9922; found 1131.9915.

IR: 761, 819, 1082, 1145, 1237, 1356, 1401, 1465, 1488, 1509, 1602, 2852, 2923, 3440.

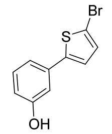

BOS5-1S2

General procedure B was used for the SMC and a mixture of THF and H_2O (v/v, 4/1) was used as the solvent. **BOS5-1S2** was obtained in 45 % yield (3.2 g) as a white solid.

^{1}H NMR ($CDCl_3$, 400 MHz): δ (ppm) 7.24 (t, J = 7.6 Hz, 1H), 7.09 (d, J = 7.6 Hz, 1H), 7.03-6.99 (m, 3H), 7.77 (dd, J_1 = 8.0 Hz, J_2 = 2.0 Hz, 1H), 5.02 (d, J = 1.6 Hz, 1H).

^{13}C NMR ($CDCl_3$, 101 MHz) δ (ppm) 156.0, 145.4, 135.3, 130.9, 130.5, 123.6, 118.5, 115.0, 112.6, 111.8.

HRMS: Calcd. For: $C_{10}H_8BrOS^+$ (M+H$^+$): 254.9474; found 254.9453.

IR: 677, 777, 792, 841, 961, 1003, 1089, 1171, 1182, 1209, 1226, 1263, 1275, 1333, 1432, 1454, 1479, 1581, 1594, 3404 cm^{-1}.

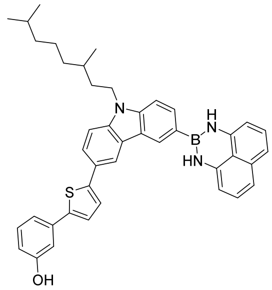

BOS5-1S3

General procedure B was used for the SMC and a mixture of THF and H_2O (v/v, 4/1) was used as the solvent. **BOS5-1S3** was obtained in 76% yield (984.5 g) as a white solid. It was directly used in the next step without characterization because of its very bad solubility in normal organic solvents.

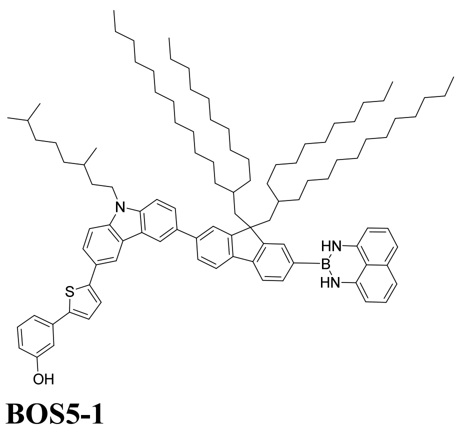

BOS5-1

General procedure A was used for the SMC. **BOS5-1** was obtained in 93% yield (3.1 mmol scale, 4.3 g) as a colorless gel.

R_f = 0.3 (Hexanes/EA = 4:1)

^{1}H NMR ($CDCl_3$, 400 MHz): δ (ppm) 8.41 (dd, J_1 = 6.4 Hz, J_2 = 1.2 Hz, 2H), 7.85-7.73 (m, 6H), 7.67-7.64 (m, 2H), 7.48 (d, J = 8.4 Hz, 1H), 7.43 (d, J = 8.4 Hz, 1H), 7.35-7.32 (m, 2H), 7.28-7.27 (m, 2H), 7.19-7.15 (m, 3H), 7.09-7.07 (m, 2H), 6.78-6.76 (m, 1H), 6.47 (d, J = 7.2 Hz, 2H), 6.07 (s, 2H), 4.91 (s, 1H), 4.43-4.30 (m, 2H), 2.16-2.15 (m, 4H), 1.98-1.90 (m, 1H), 1.77-1.68 (m, 1H), 1.64-1.51 (m, 3H), 1.44-1.35 (m, 2H), 1.25-1.02 (m, 62H), 0.94-0.72 (m, 44H), 0.67-0.63 (m, 2H). ^{13}C NMR ($CDCl_3$, 101 MHz) δ (ppm) 156.1, 151.5, 150.5, 145.5, 143.7, 141.9, 141.3, 141.0, 140.5, 140.4, 139.6, 136.6, 136.3, 133.3, 130.3, 127.7, 127.0, 126.3, 125.8, 124.4, 124.3, 123.7, 123.5, 123.2, 123.0, 120.4, 120.0, 119.6, 119.1, 118.4, 117.9, 117.8, 114.3, 112.4, 109.2, 109.1, 106.1, 55.3, 45.1, 41.6, 39.4, 37.3, 35.9, 35.11, 35.07, 33.6, 32.07, 32.04, 32.02, 31.10, 31.08, 30.02, 29.98, 29.87, 29.84, 29.83, 29.82, 29.80, 29.78, 29.71, 29.52, 29.50, 29.48, 28.1, 26.33, 26.29, 24.8, 22.83, 22.82, 22.78, 22.75, 19.9, 14.29, 14.27, 14.24.

^{11}B NMR ($CDCl_3$, 128 MHz): δ (ppm) 31.2.

HRMS: Calcd. For: $C_{103}H_{146}BN_3OS^+$ (M^+): 1485.1313; found 1485.1315.

IR: 662, 686, 761, 802, 819, 1083, 1165, 1214, 1234, 1293, 1326, 1375, 1407, 1463, 1510, 1602, 1629, 2852, 2923, 3053, 3439 cm^{-1}.

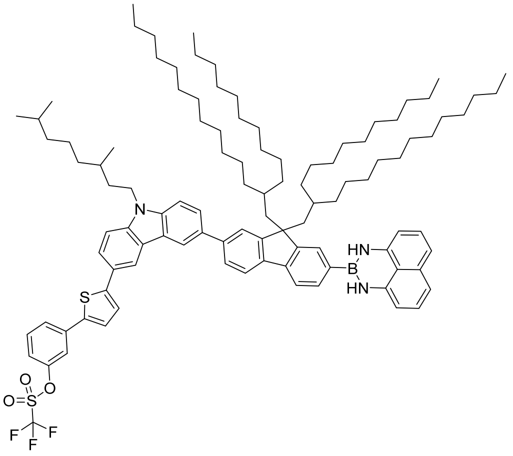

BOS5-1b

General procedure B was used for the triflation. **BOS5-1b** was obtained in 97% yield (1.252 mmol scale, 1.97 g) as a colorless gel.

R_f = 0.3 (Hexanes/DCM = 2:1)

^{1}H NMR ($CDCl_3$, 400 MHz): δ (ppm) 8.41 (dd, J_1 = 8.0 Hz, J_2 = 1.6 Hz, 2H), 7.85-7.72 (m, 6H), 7.68-8.64 (m, 3H), 7.55 (t, J = 2.0 Hz, 1H), 7.50-7.43 (m, 3H), 7.39-7.36 (m,

2H), 7.20-7.15 (m, 3H), 7.08-7.06 (m, 2H), 6.47 (d, *J* = 7.2 Hz, 2H), 6.06 (s, 2H), 4.44-4.31 (m, 2H), 2.19-2.10 (m, 4H), 1.98-1.90 (m, 1H), 1.78-1.70 (m, 1H), 1.66-1.61 (m, 1H), 1.57-1.49 (m, 1H), 1.46-1.35 (m, 2H), 1.30-1.02 (m, 64H), 0.94-0.77 (m, 40H), 0.66-0.61 (m, 3H).

^{13}C NMR ($CDCl_3$, 101 MHz) δ (ppm) 151.6, 150.5, 150.2, 147.0, 143.7, 141.3, 140.9, 140.7, 140.4, 139.7, 139.6, 137.4, 136.6, 133.4, 130.8, 130.3, 127.7, 127.0, 126.4, 125.9, 125.6, 125.32, 125.30, 124.3, 123.8, 123.5, 123.2, 120.5, 120.4, 120.0, 119.6, 119.5, 119.1, 118.1, 118.0, 117.9, 117.3, 109.3, 109.1, 106.1, 55.3, 45.1, 41.7, 39.4, 37.3, 35.9, 35.1, 33.6, 32.09, 32.07, 32.05, 32.02, 31.1, 30.02, 29.99, 29.87, 29.85, 29.82, 29.79, 29.78, 29.72, 29.53, 29.51, 29.50, 29.48, 28.1, 26.33, 26.29, 24.8, 22.85, 22.82, 22.78, 22.75, 19.9, 14.29, 14.26, 14.23.

^{11}B NMR ($CDCl_3$, 128 MHz): δ (ppm) 31.2.

HRMS: Calcd. For: $C_{104}H_{146}BF_3N_3O_3S_2^+$ (M^+): 1618.0879; found 1618.0880.

IR: 606, 801, 821, 886, 1084, 1143, 1214, 1246, 1425, 1461, 1602, 2852, 2923, 2954, 3053, 3436 cm^{-1}.

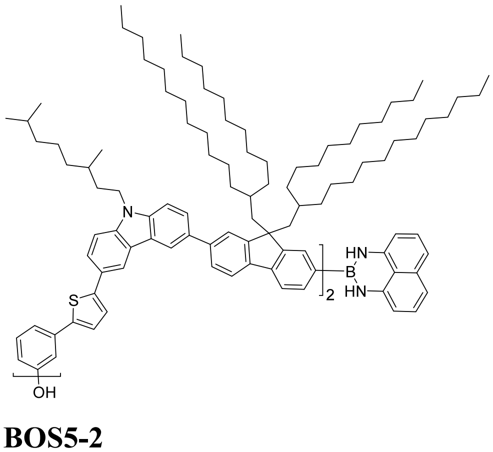

BOS5-2

General procedure B was used for the triflation step (95% yield). General procedure A was used for the SMC step. **BOS5-2** was obtained in 87% yield over two steps (1.2 mmol scale, 2.9 g) as a colorless gel.

R_f = 0.3 (Hexanes/EA = 5:1)

^{1}H NMR ($CDCl_3$, 400 MHz): δ (ppm) 8.46-8.45 (m, 1H), 8.42-8.41 (m, 3H), 7.96 (s, 1H), 7.85-7.73 (m, 12H), 7.69-7.63 (m, 5H), 7.59-7.57 (m, 1H), 7.52-7.47 (m, 3H), 7.46-7.42 (m, 3H), 7.40-7.39 (m, 1H), 7.35-7.32 (m, 2H), 7.28-7.27 (m, 2H), 7.18-7.14 (m, 3H), 7.08-7.06 (m, 2H), 6.78-6.75 (m, 1H), 6.46 (d, *J* = 6.8 Hz, 2H), 6.06 (s, 2H), 4.78 (s, 1H), 4.45-4.31 (m, 4H), 2.21-2.11 (m, 8H), 1.99-1.90 (m, 2H), 1.79-1.69 (m,

2H), 1.65-1.59 (m, 2H), 1.58-1.49 (m, 2H), 1.47-1.35 (m, 5H), 1.30-1.03 (m, 125H), 0.96-0.61 (m, 88H).

MALDI-TOF-MS: Calcd. For: $C_{196}H_{283}BN_4S_2^+$ (M^+): 2786.18; found 2786.12.

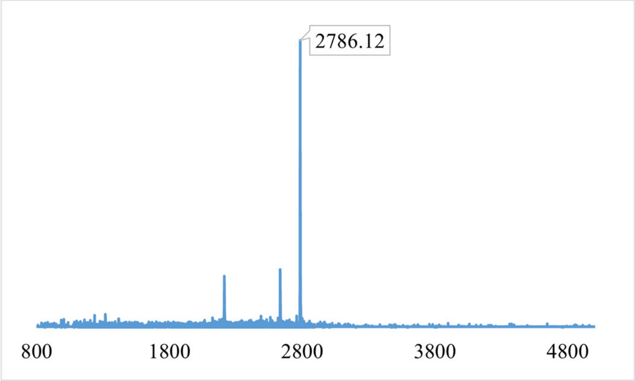

Figure S21. MALDI-TOF-MS spectrum of **BOS3-2**

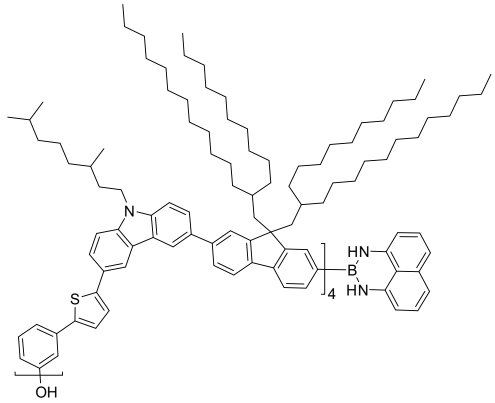

BOS5-4

General procedure B was used for the triflation step (93% yield). General procedure A was used for the SMC step. **BOS5-4** was obtained in 82% yield over two steps (0.42 mmol scale, 1.9 g) as a white solid.

R_f = 0.4 (Hexanes/EA = 5:1)

^{1}H NMR ($CDCl_3$, 400 MHz): δ (ppm) 8.45-8.40 (m, 8H), 7.96 (m, 3H), 7.84-7.63 (m, 35H), 7.51-7.38 (m, 17H), 7.34-7.32 (m, 2H), 7.28-7.27 (m, 2H), 7.18-7.14 (m, 3H), 7.08-7.06 (m, 2H), 6.78-6.73 (m, 1H), 6.46 (d, J = 6.8 Hz, 2H), 6.05 (s, 2H), 4.75 (s, 1H), 4.44-4.30 (m, 8H), 2.15-2.12 (m, 16H), 1.99-1.89 (m, 4H), 1.78-1.55 (m, 8H), 1.58-1.49 (m, 4H), 1.44-1.35 (m, 6H), 1.28-0.61 (m, 430H).

MALDI-TOF-MS: Calcd. For: $C_{382}H_{557}BN_6S_4^+$ (M^+): 5388.28; found 5389.24.

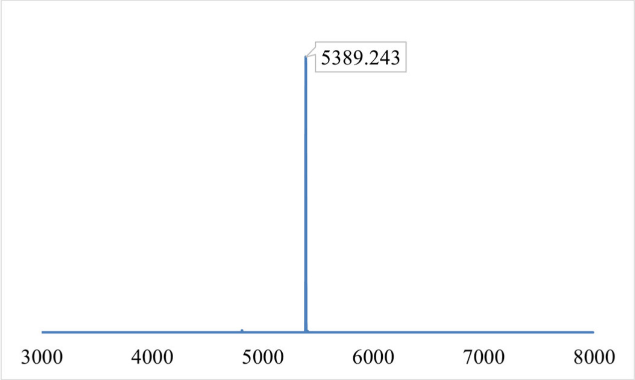

Figure S22. MALDI-TOF-MS spectrum of **BOS3-4**

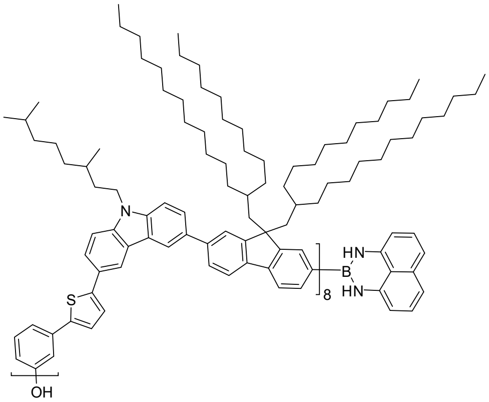

BOS5-8

General procedure B was used for the triflation step (90% yield). General procedure A was used for the SMC step. **BOS5-8** was obtained in 79% yield over two steps (0.13 mmol scale, 1.1 g) as a white solid.

R_f = 0.3 (Hexanes/DCM = 1:2)

^{1}H NMR ($CDCl_3$, 400 MHz): δ (ppm) 8.46-8.40 (m, 16H), 7.96 (m, 6H), 7.85-7.31 (m, 124H), 7.28-7.27 (m, 2H), 7.18-7.14 (m, 3H), 7.08-7.05 (m, 2H), 6.46 (d, J = 6.8 Hz, 2H), 6.05 (s, 2H), 4.77 (s, 1H), 4.44-4.31 (m, 16H), 2.15-2.12 (m, 32H), 1.99-1.90 (m, 8H), 1.78-1.59 (m, 16H), 1.58-1.49 (m, 8H), 1.46-1.35 (m, 12H), 1.30-0.95 (m, 560H), 0.89-0.68 (m, 300H).

MALDI-TOF-MS: Calcd. For: $C_{754}H_{1105}BN_{10}S_8{}^+$ (M^+): 10588.57; found 10595.01.

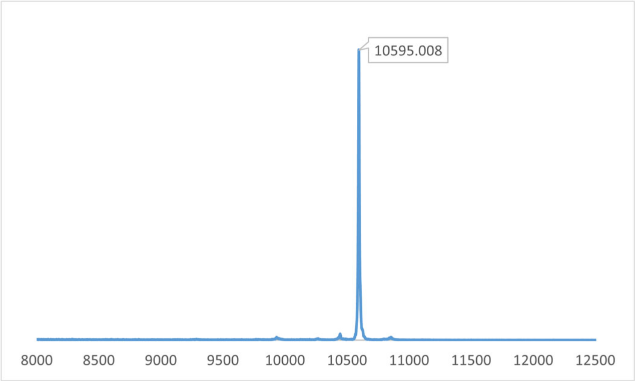

Figure S23. MALDI-TOF-MS spectrum of **BOS3-8**

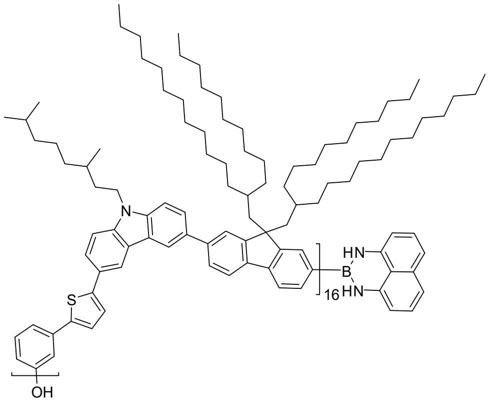

BOS5-16

General procedure B was used for the triflation step (90% yield). General procedure A was used for the SMC step. **BOS5-16** was obtained in 76% yield over two steps (0.048 mmol scale, 765.9 mg) as a white solid.

R_f = 0.3 (Hexanes/DCM = 1:2)

^{1}H NMR ($CDCl_3$, 400 MHz): δ (ppm) 8.46-8.40 (m, 32H), 7.96 (s, 16H), 7.85-7.73 (m, 95H), 7.69-7.63 (m, 47H), 7.59-7.57 (m, 16H), 7.51-7.39 (m, 76H), 7.34-7.32 (m, 2H), 7.28-7.27 (m, 2H), 7.18-7.14 (m, 3H), 7.08-7.06 (m, 2H), 6.77-6.74 (m, 1H), 6.46 (d, *J* = 6.8 Hz, 2H), 6.06 (s, 2H), 4.75 (s, 1H), 4.44-4.31 (m, 32H), 2.16-1.59 (m, 112H), 1.58-0.70 (m, 1794H).

MALDI-TOF-MS: Calcd. For: $C_{1498}H_{2201}BN_{18}OS_{16}{}^{+}$ (M^{+}): 20995.7703; found 21013.41.

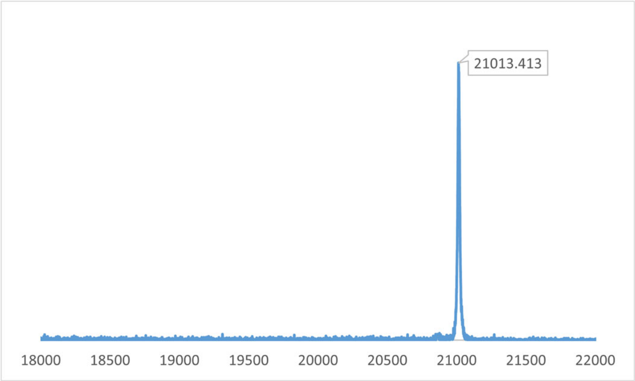

Figure S24. MALDI-TOF-MS spectrum of **BOS3-16**

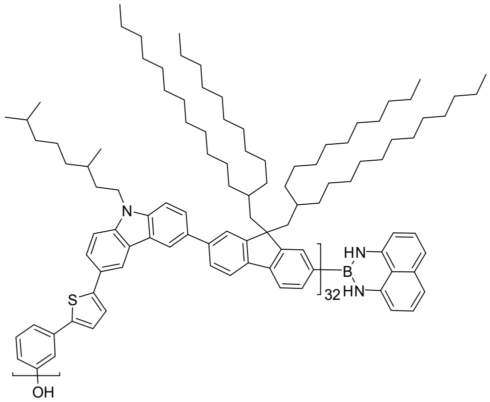

BOS5-32

General procedure B was used for the triflation step (89% yield). General procedure A was used for the SMC step. **BOS5-32** was obtained in 82% yield over two steps (0.0024 mmol scale, 82.2 mg) as a white solid.

R_f = 0.4 (Hexanes/DCM = 1:2)

^{1}H NMR ($CDCl_3$, 400 MHz): δ (ppm) 8.46-8.43 (m, 64H), 7.96 (m, 32H), 7.85-7.66 (m, 283H), 7.59-7.57 (m, 32H), 7.52-7.39 (m, 160H), 7.35-7.33 (m, 3H), 7.18-7.06 (m, 5H), 6.77-6.74 (m, 1H), 6.47 (d, J = 7.2 Hz, 2H), 6.06 (s, 2H), 4.75 (s, 1H), 4.38 (m, 64H), 2.17 (m, 128H), 1.99-1.89 (m, 32H), 1.79-1.60 (m, 64H), 1.47-1.36 (m, 64H), 1.25-0.78 (m, 3369H), 0.74-0.66 (m, 96H).

MALDI-TOF-MS: Calcd. For: $C_{2986}H_{4393}N_{34}S_{32}BO^{+}$ (M^{+}): 41816.10; found 41816.35.

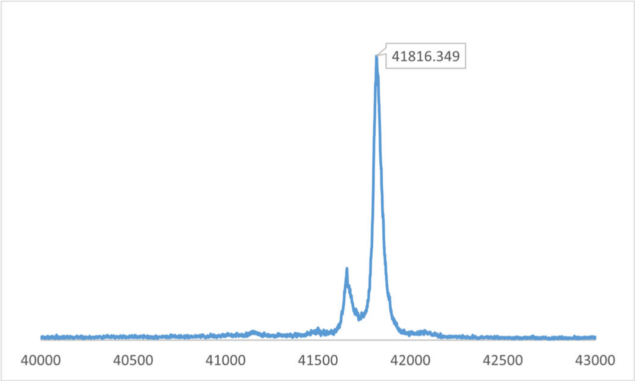

Figure S25. MALDI-TOF-MS spectrum of **BOS3-32**

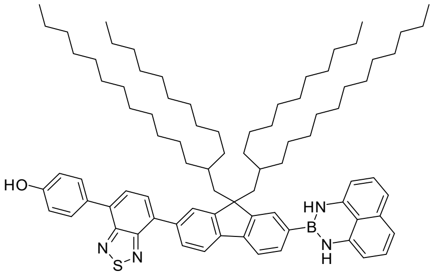

BOS6-1

General procedure A was used for the SMC. **BOS6-1** was obtained in 65% yield (1.8 mmol scale, 1.44 g) as a yellow solid.

R_f = 0.4 (Hexanes/EA = 4:1)

^{1}H NMR ($CDCl_3$, 400 MHz): δ (ppm) 8.04-8.03 (m, 2H), 7.93-7.88 (m, 3H), 7.84-7.82 (m, 2H), 7.76-7.75 (m, 1H), 7.68-7.64 (m, 2H), 7.18-7.14 (m, 2H), 7.08-7.02 (m, 4H), 6.46 (d, J = 7.2 Hz, 2H), 6.05 (s, 2H), 4.94 (s, 1H), 2.14-2.13 (m, 4H), 1.27-0.63 (m, 94H).

^{13}C NMR ($CDCl_3$, 101 MHz) δ (ppm) 156.0, 154.4, 151.1, 150.8, 143.4, 141.3, 136.6, 136.3, 133.4, 132.7, 130.8, 130.44, 130.35, 128.4, 127.9, 127.7, 127.5, 127.1, 125.3, 120.1, 120.0, 119.9, 118.0, 115.7, 106.1, 55.4, 45.0, 35.2, 35.0, 33.6, 32.08, 32.07, 32.04, 30.01, 29.97, 29.87, 29.79, 29.75, 29.74, 29.52, 29.50, 29.48, 26.3, 26.2, 22.84, 22.81, 14.28, 14.26.

^{11}B NMR ($CDCl_3$, 128 MHz): δ (ppm) 31.2.

HRMS: Calcd. For: $C_{83}H_{119}BN_4OS^+$ (M^+): 1230.9198; found 1230.9197.

IR: 761, 819, 1082, 1175, 1233, 1328, 1375, 1408, 1466, 1510, 1602, 1629, 2851, 2922, 3438, 3583 cm^{-1}.

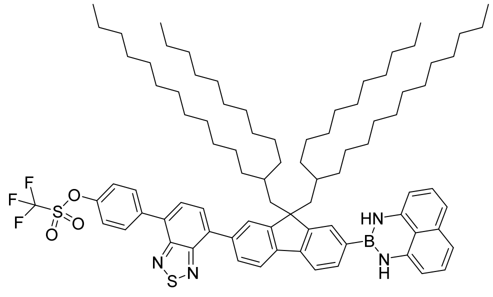

BOS6-1b

General procedure B was used for the triflation. **BOS6-1b** was obtained in 93% yield (1.03 mmol scale, 1.31 g) as a yellow solid.

R_f = 0.3 (Hexanes/DCM = 2:1)

^{1}H NMR ($CDCl_3$, 400 MHz): δ (ppm) 8.12-8.03 (m, 4H), 7.91-7.81 (m, 4H), 7.68-7.65 (m, 2H), 7.48-7.46 (m, 2H), 7.18-7.14 (m, 2H), 7.08-7.06 (m, 2H), 6.46 (d, J = 7.2 Hz, 2H), 6.05 (s, 2H), 2.15-2.13 (m, 4H), 1.26-0.62 (m, 94H).

^{13}C NMR ($CDCl_3$, 101 MHz) δ (ppm) 154.3, 154.0, 151.2, 150.8, 149.6, 143.2, 141.7, 141.3, 138.0, 136.6, 135.8, 134.9, 131.1, 130.9, 130.4, 128.7, 128.5, 127.7, 127.6, 127.1, 125.4, 121.7, 120.6, 120.2, 120.0, 119.96, 118.0, 106.2, 55.4, 45.0, 35.2, 35.0, 33.6, 32.1, 32.0, 30.0, 29.96, 29.87, 29.81, 29.79, 29.78, 29.74, 29.52, 29.51, 29.49, 29.47, 26.29, 26.25, 22.85, 22.82, 22.79, 14.29, 14.26, 14.25, 14.22.

^{11}B NMR ($CDCl_3$, 128 MHz): δ (ppm) 31.5.

F^{19} NMR ($CDCl_3$, 128 MHz): δ (ppm) -72.7.

HRMS: Calcd. For: $C_{84}H_{118}BF_3N_4O_3S_2^+$ (M^+): 1362.8691; found 1362.8692.

IR: 761, 819, 887, 1083, 1142, 1215, 1250, 1375, 1425, 1466, 1509, 1602, 1629, 2852, 2923, 2954, 3438 cm^{-1}.

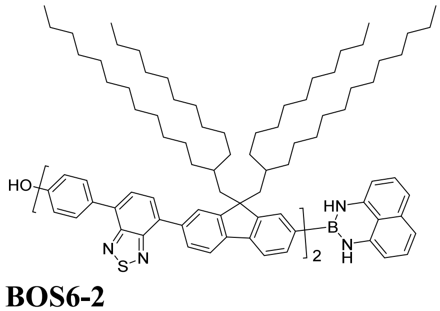

BOS6-2

General procedure B was used for the triflation step (93% yield). General procedure A was used for the SMC step. **BOS6-2** was obtained in 82% yield over two steps (1.03 mmol scale, 1.92 g) as a yellow solid.

R_f = 0.4 (Hexanes/EA = 5:1)

^{1}H NMR ($CDCl_3$, 400 MHz): δ (ppm) 8.15-8.13 (m, 2H), 8.07-8.05 (m, 4H), 7.94-7.84 (m, 11H), 7.77-7.74 (m, 2H), 7.71-7.65 (m, 3H), 7.19-7.15 (m, 2H), 7.09-7.02 (m, 4H), 6.47 (d, J = 6.8 Hz, 2H), 6.06 (s, 2H), 4.94 (s, 1H), 2.20-2.09 (m, 8H), 1.18-0.65 (m, 188H).

^{13}C NMR (101 MHz, $CDCl_3$) 156.0, 154.5, 154.4, 151.8, 151.19, 151.15, 150.8, 144.2, 143.4, 141.9, 141.40, 141.37, 141.29, 140.7, 139.3, 136.6, 136.33, 136.25, 135.8, 134.0, 133.5, 132.8, 132.6, 130.8, 130.5, 130.4, 129.7, 128.44, 128.40, 128.0, 127.88, 127.85, 127.7, 127.48, 127.46, 127.09, 127.07, 126.3, 125.3, 125.2, 123.13, 123.11, 120.4, 120.2, 120.0, 119.9, 118.0, 115.7, 106.2, 55.5, 55.4, 45.3, 45.1, 35.18, 35.13, 35.06, 34.9, 33.6, 32.08, 32.05, 30.02, 29.99, 29.98, 29.87, 29.84, 29.80, 29.75, 29.55, 29.53, 29.51, 29.49, 26.3, 26.2, 22.85, 22.83, 22.82, 14.28, 14.27.

^{11}B NMR ($CDCl_3$, 128 MHz): δ (ppm) 31.2.

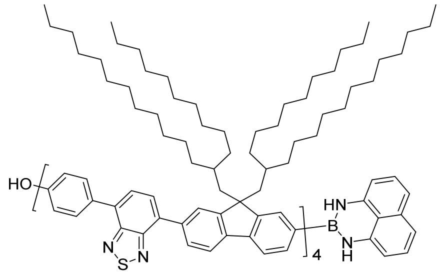

BOS6-4

General procedure B was used for the triflation step (96% yield). General procedure A was used for the SMC step. **BOS6-4** was obtained in 85% yield over two steps (0.24 mmol scale, 905 mg) as a yellow solid.

R_f = 0.2 (Hexanes/DCM = 1:2)

^{1}H NMR ($CDCl_3$, 400 MHz): δ (ppm) 8.15-8.04 (m, 14H), 7.94-7.83 (m, 23H), 7.77-7.65 (m, 9H), 7.18-7.02 (m, 6H), 6.47 (d, J = 7.2 Hz, 2H), 6.06 (s, 2H), 4.93 (s, 1H), 2.16 (m, 16H), 1.18-0.65 (m, 376 H).

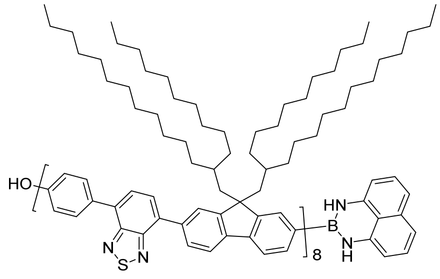

BOS6-8

General procedure B was used for the triflation step (92% yield). General procedure A

was used for the SMC step. **BOS6-8** was obtained in 86% yield over two steps (670.4 mg) as a yellow solid.

R_f = 0.3 (Hexanes/DCM = 1:2)

^{1}H NMR ($CDCl_3$, 400 MHz): δ (ppm) 8.15-7.65 (m, 94H), 7.18-7.15 (m, 2H), 7.09-7.02 (m, 4H), 6.47 (d, J = 6.8 Hz, 2H), 6.06 (s, 2H), 4.90 (s, 1H), 2.16 (m, 32H), 1.29-0.66 (m, 752H).

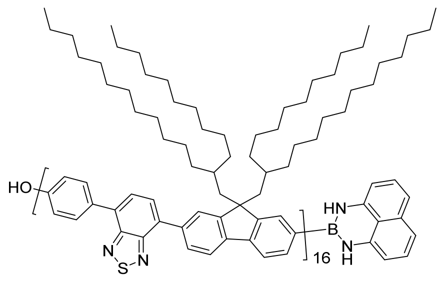

BOS6-16

General procedure B was used for the triflation step (95% yield). General procedure A was used for the SMC step. **BOS6-16** was obtained in 83% yield over two steps (0.035 mmol scale, 494.0 mg) as a yellow solid.

R_f = 0.3 (Hexanes/DCM = 1:2)

^{1}H NMR ($CDCl_3$, 400 MHz): δ (ppm) 8.14-7.65 (m, 190H), 7.18-7.15 (m, 2H), 7.08-7.00 (m, 4H), 6.47 (d, J = 6.8 Hz, 2H), 6.06 (s, 2H), 4.95 (s, 1H), 2.88-1.86 (m, 64H), 1.29-0.70 (m, 1504H).

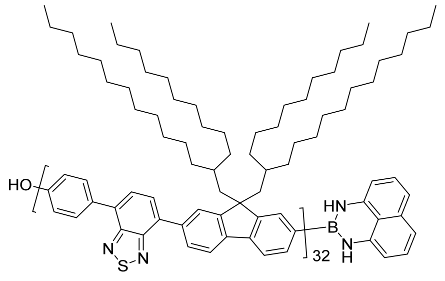

BOS6-32

General procedure B was used for the triflation step (92% yield). General procedure A was used for the SMC step. **BOS6-32** was obtained in 75% yield over two steps (0.003 mmol scale, 75.7 mg) as a yellow solid.

R_f = 0.3 (Hexanes/DCM = 1:2)

^{1}H NMR ($CDCl_3$, 400 MHz): δ (ppm) 8.14-7.66 (m, 382H), 7.19-7.15 (m, 2H), 7.09-7.02 (m, 4H), 6.47 (d, J = 6.8 Hz, 2H), 6.06 (s, 2H), 4.90 (s, 1H), 2.68-1.84 (m, 128H), 1.19-0.76 (m, 3006H).

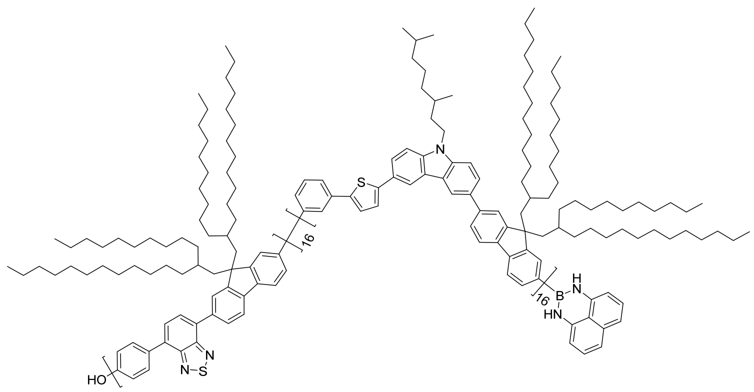

BOS(5, 6)-16

General procedure A was used for the SMC. **BOS(5, 6)-16** was obtained in 82% yield (0.00286 mmol scale, 88.5 mg) as a yellow solid.

R_f = 0.2 (Hexanes/DCM = 1:2)

^{1}H NMR ($CDCl_3$, 400 MHz): δ (ppm) 8.45-8.42 (m, 32H), 8.14-8.05 (m, 60H), 7.95-7.65 (m, 282H), 7.58-7.56 (m, 18H), 7.51-7.39 (m, 80H), 7.17-7.13 (m, 2H), 7.07-7.00 (m, 4H), 6.46 (d, J = 6.4 Hz, 2H), 6.05 (s, 2H), 4.90 (s, 1H), 4.45-4.29 (m, 32H), 2.16-1.90 (m, 160H), 1.75-1.62 (m, 64H), 1.37-0.72 (m, 3250H).

VI. UV-Vis absorption measurements of the representative products

UV-Vis spectra were measured on a Agilent Cary 5000 spectrophotometer. The baseline was corrected by subtracting a measurement of the cuvette filled with pure solvent used for the measurement. The samples were measured in CH_2Cl_2 solution at room temperature.

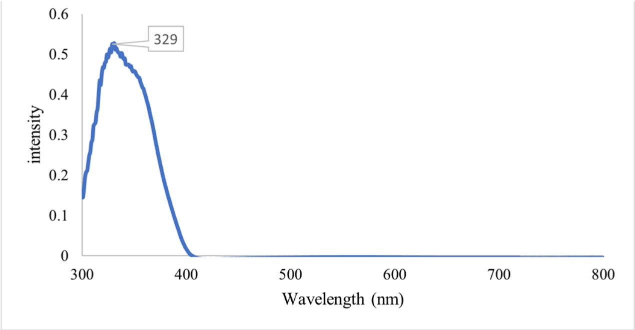

Figure S26. UV-Vis absorption spectrum of **BOS5-1** (10.0 μM)

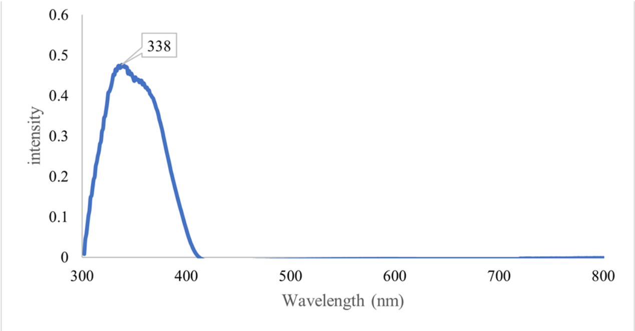

Figure S27. UV-Vis absorption spectrum of **BOS5-2** (5.0 μM)

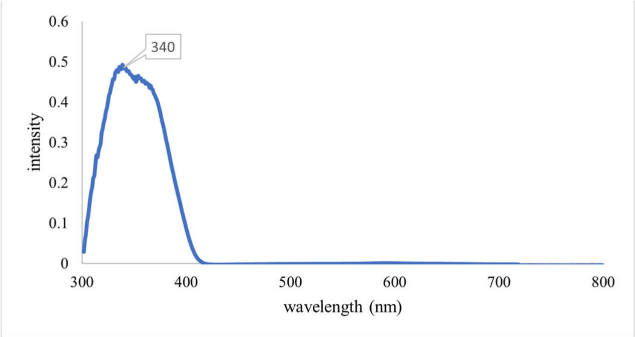

Figure S28. UV-Vis absorption spectrum of **BOS5-4** (2.5 μM)

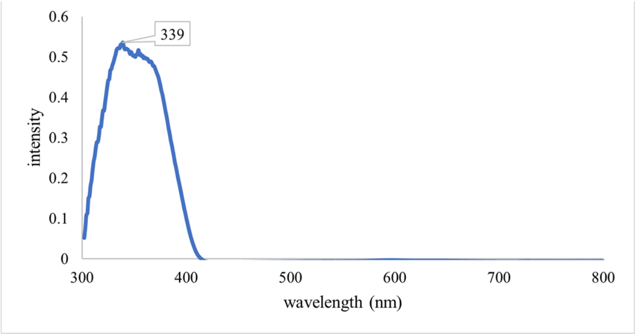

Figure S29. UV-Vis absorption spectrum of **BOS5-8** (1.25 μM)

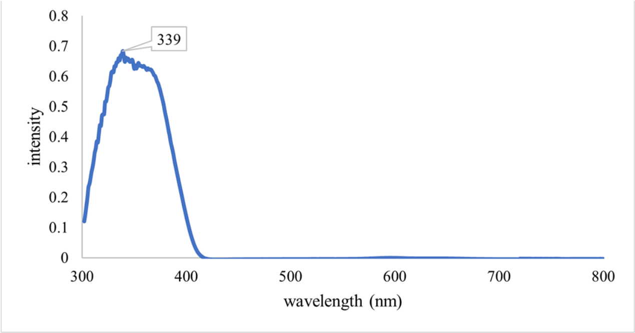

Figure S30. UV-Vis absorption spectrum of **BOS5-16** (0.625 μM)

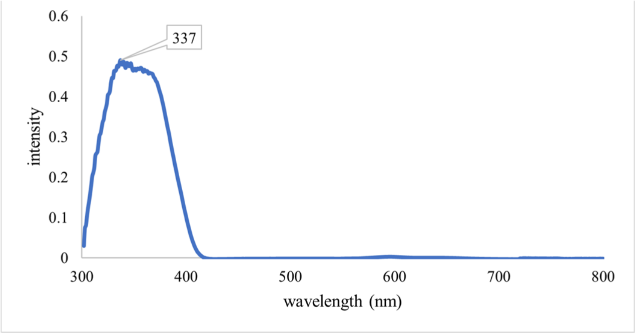

Figure S31. UV-Vis absorption spectrum of **BOS5-32** (0.3125 μM)

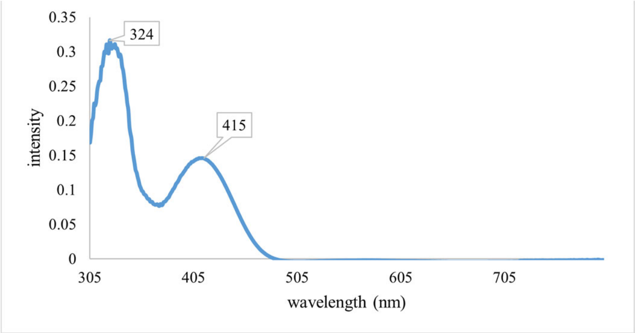

Figure S32. UV-Vis absorption spectrum of **BOS6-1** (10.0 μM)

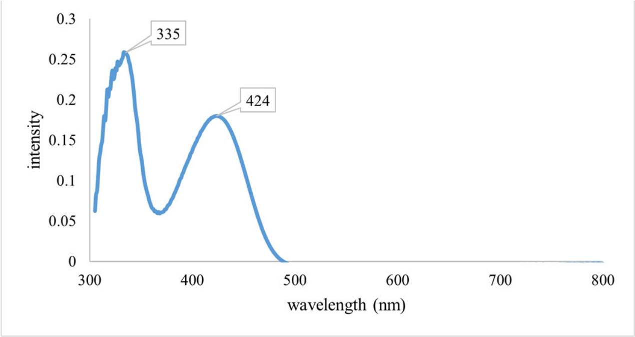

Figure S33. UV-Vis absorption spectrum of **BOS6-2** (5.0 μM)

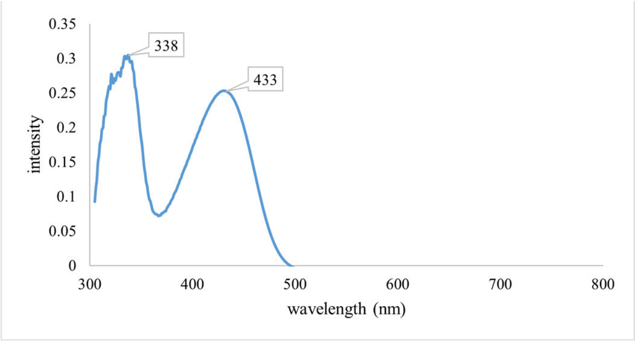

Figure S34. UV-Vis absorption spectrum of **BOS6-4** (2.5 μM)

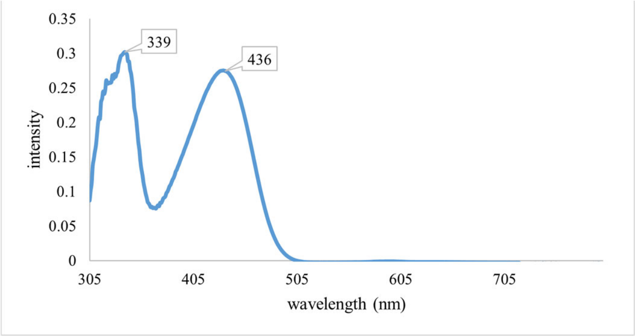

Figure S35. UV-Vis absorption spectrum of **BOS6-8** (1.25 μM)

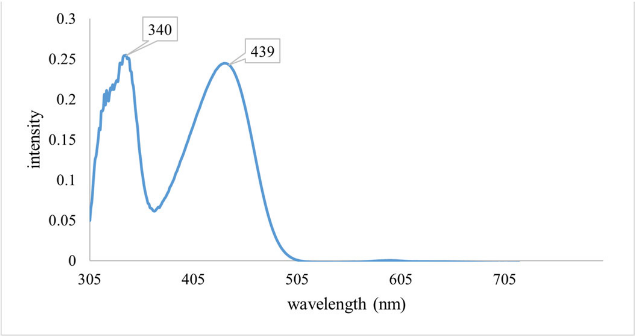

Figure S36. UV-Vis absorption spectrum of **BOS6-16** (0.625 μM)

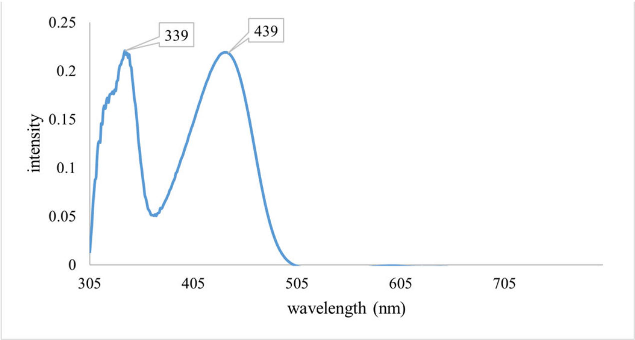

Figure S37. UV-Vis absorption spectrum of **BOS6-32** (0.3125 μM)

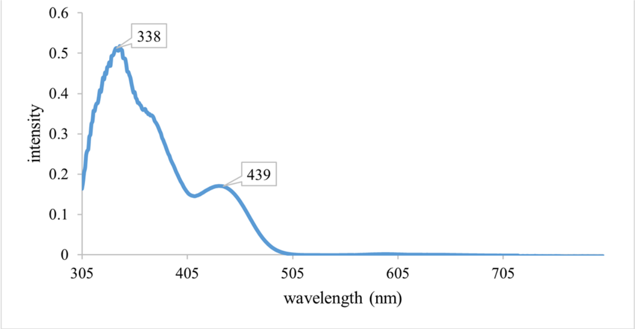

Figure S38. UV-Vis absorption spectrum of **BOS(5,6)-16** (0.3125 μM)

VII. GPC measurements of the representative products

Size exclusion chromatography (SEC) for polymer molecular weight analysis (based on polystyrene standard) was carried out with an Agilent 1260 Infinity system (VWD UV detector) and two 300 x 7.5 mm ResiPore GPC columns eluted with THF (HPLC grade, Sigma-Aldrich). Flow rate was 1.0 mL/min and the column temperature was maintained at 35 ºC. The sample concentration used for GPC measurements was 1.0 mg/mL.

The shoulders at the higher molecular weight regions in the SEC curves could be attributed to the aggregations of the polymers (*J. Am. Chem. Soc.* **2016**, *138*, 9369–9372, cited as ref 13m); however, at this stage, the existence of higher molecular weight impurities cannot be excluded. The shoulders at the lower molecular weight regions in certain samples could be attributed to a very small amount of shorter polymer left in the previous deprotection step.

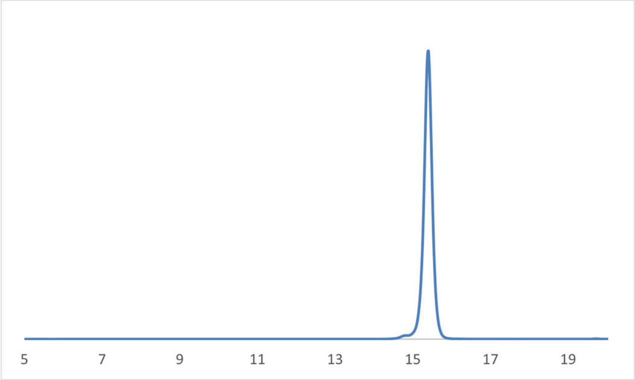

Figure S39. GPC trace of **BOS2-34**

Table S1. Molecular weight distribution analysis of **BOS2-34**

$M_{Calcd.}$ (Da)	M_n (Da)	M_w (Da)	PDI
7000.2	25558	25970	1.02

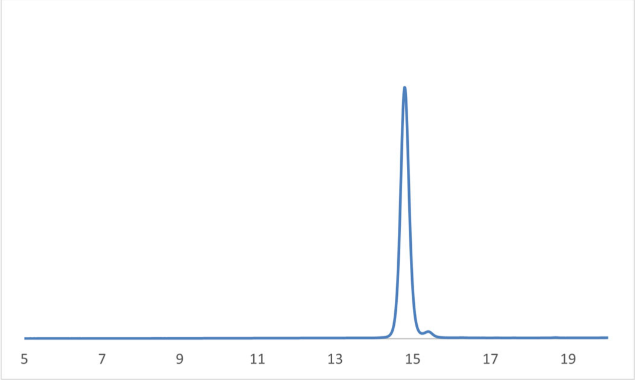

Figure S40. GPC trace of **BOS2-68**

Table S2. Molecular weight distribution analysis of **BOS2-68**

$M_{Calcd.}$ (Da)	M_n (Da)	M_w (Da)	PDI
13813.9	42534	43397	1.02

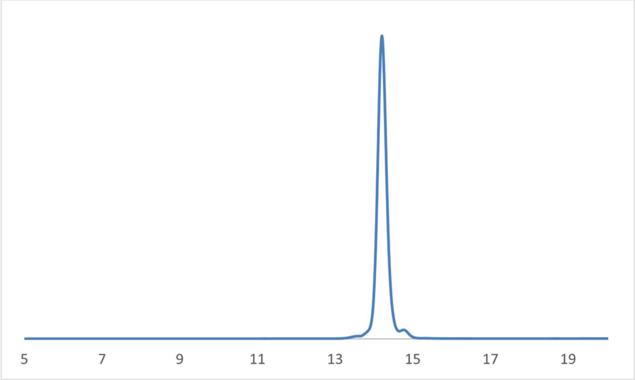

Figure S41. GPC trace of **BOS2-136**

Table S3. Molecular weight distribution analysis of **BOS2-136**

$M_{Calcd.}$ (Da)	M_n (Da)	M_w (Da)	PDI
27451.0	72230	74035	1.03

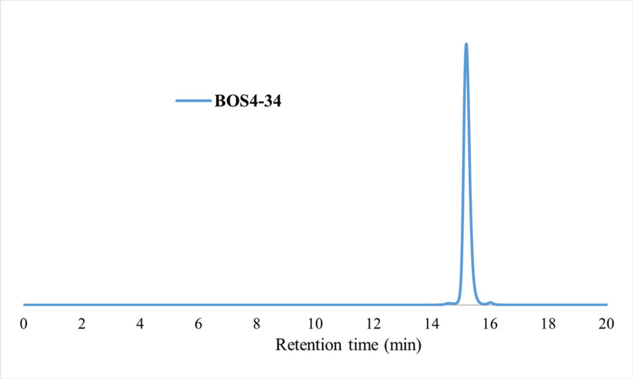

Figure S42. GPC trace of **BOS4-34**

Table S4. Molecular weight distribution analysis of **BOS4-34**

$M_{Calcd.}$ (Da)	M_n (Da)	M_w (Da)	PDI
7000.2	29409	29925	1.02

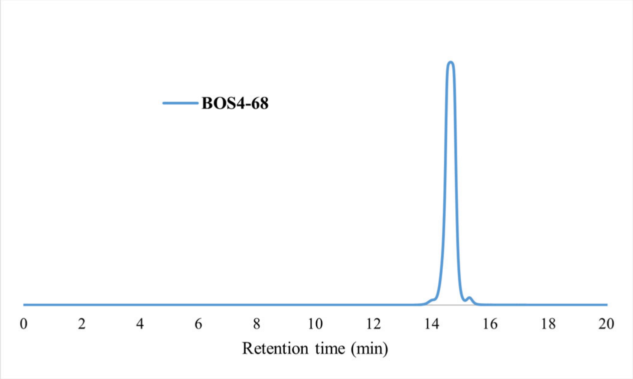

Figure S43. GPC trace of **BOS4-68**

Table S5. Molecular weight distribution analysis of **BOS4-68**

$M_{Calcd.}$ (Da)	M_n (Da)	M_w (Da)	PDI
13813.9	48783	50364	1.03

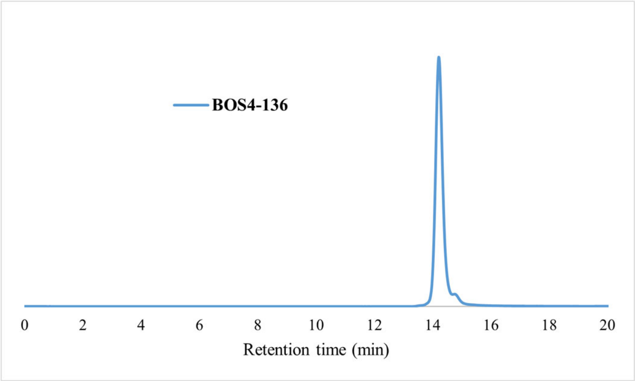

Figure S44. GPC trace of **BOS4-136**

Table S6. Molecular weight distribution analysis of **BOS4-136**

$M_{Calcd.}$ (Da)	M_n (Da)	M_w (Da)	PDI
27451.0	67845	70594	1.04

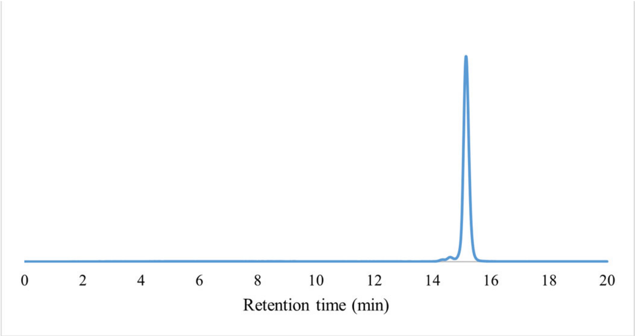

Figure S45. GPC trace of **BOS5-8**

Table S7. Molecular weight distribution analysis of **BOS5-8**

$M_{Calcd.}$ (Da)	M_n (Da)	M_w (Da)	PDI
10588.6	31470	32045	1.02

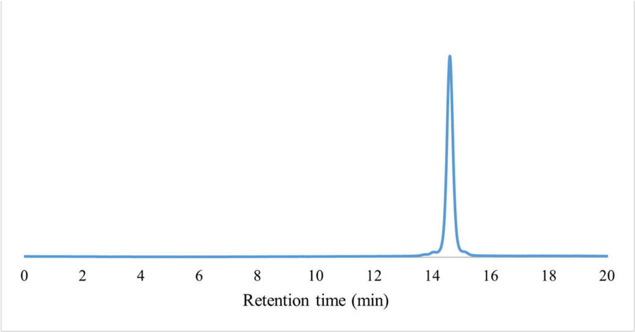

Figure S46. GPC trace of **BOS5-16**

Table S8. Molecular weight distribution analysis of **BOS5-16**

$M_{Calcd.}$ (Da)	M_n (Da)	M_w (Da)	PDI
20995.8	51304	52797	1.03

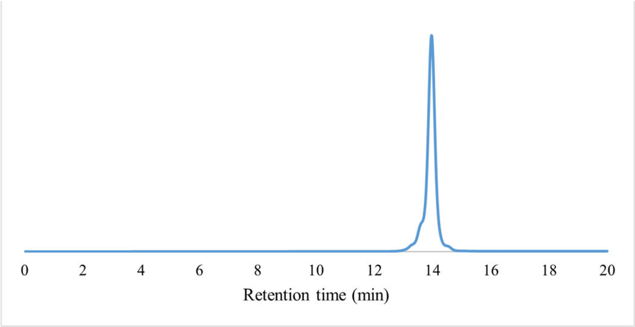

Figure S47. GPC trace of **BOS5-32**

Table S9. Molecular weight distribution analysis of **BOS5-32**

$M_{Calcd.}$ (Da)	M_n (Da)	M_w (Da)	PDI
41816.1	92741	96552	1.04

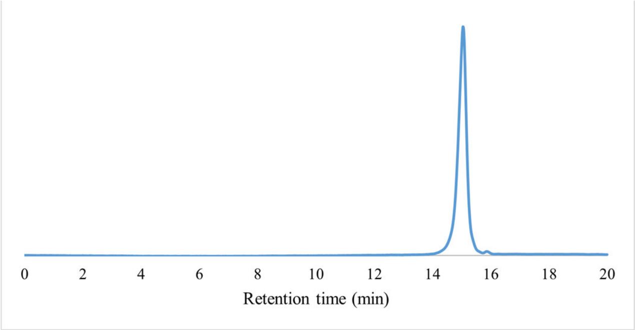

Figure S48. GPC trace of **BOS6-8**

Table S10. Molecular weight distribution analysis of **BOS6-8**

$M_{Calcd.}$ (Da)	M_n (Da)	M_w (Da)	PDI
8561.8	35163	36580	1.04

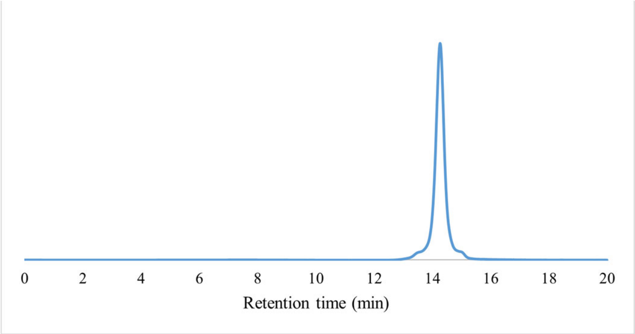

Figure S49. GPC trace of **BOS6-16**

Table S11. Molecular weight distribution analysis of **BOS6-16**

$M_{Calcd.}$ (Da)	M_n (Da)	M_w (Da)	PDI
16928.8	67918	72961	1.07

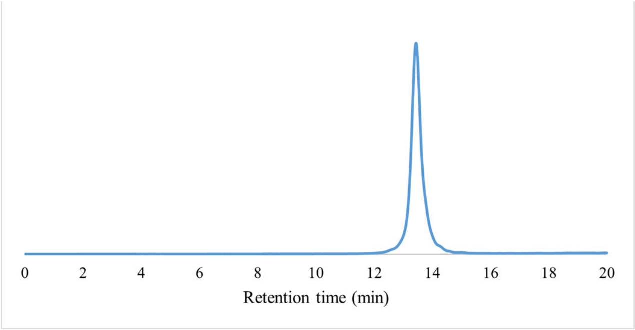

Figure S50. GPC trace of **BOS6-32**

Table S12. Molecular weight distribution analysis of **BOS6-32**

$M_{Calcd.}$ (Da)	M_n (Da)	M_w (Da)	PDI
33678.5	136319	147110	1.08

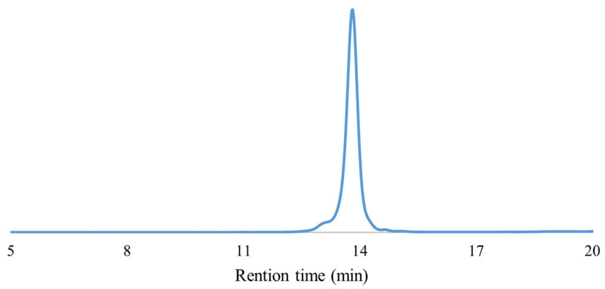

Figure S51. GPC trace of **BOS(5,6)-16**

Table S13. Molecular weight distribution analysis of **BOS(5,6)-16**

$M_{Calcd.}$ (Da)	M_n (Da)	M_w (Da)	PDI
37747.4	106802	112867	1.06

VIII. Cyclic voltammetry (CV) measurements

General procedure: Cyclic voltammogram measurements were conducted on CHI660E using a platinum plate working electrode, an Ag/Ag^+ (0.01 M of $AgNO_3$ in CH_3CN) reference electrode, and a platinum wire counter electrode. Electrochemical experiments were carried out in dry HPLC grade CH_2Cl_2 using ferrocene/ferrocenium as reference. Unless otherwise stated, the cyclic voltammogram measurements were performed at a scan rate of 100 mV/s and the supporting electrolyte was tetrabutylammonium hexafluorophosphate ($TBAPF_6$, 0.1 M) in all cases.

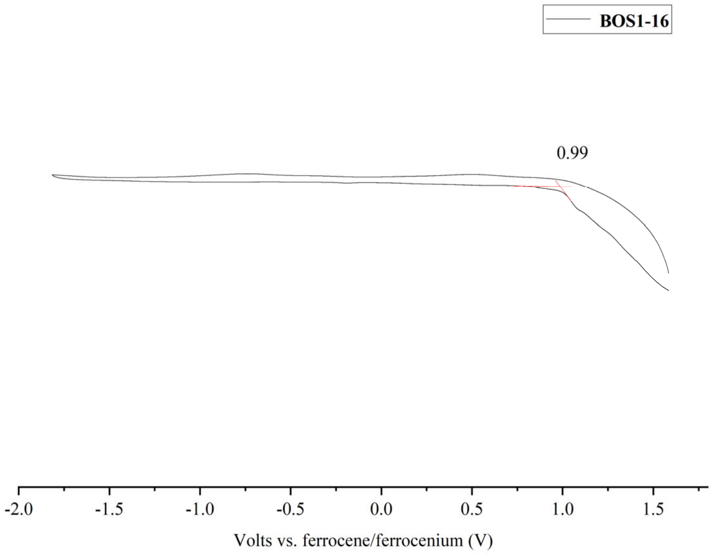

Figure S52. Cyclic voltammogram spectrum of **BOS1-16**

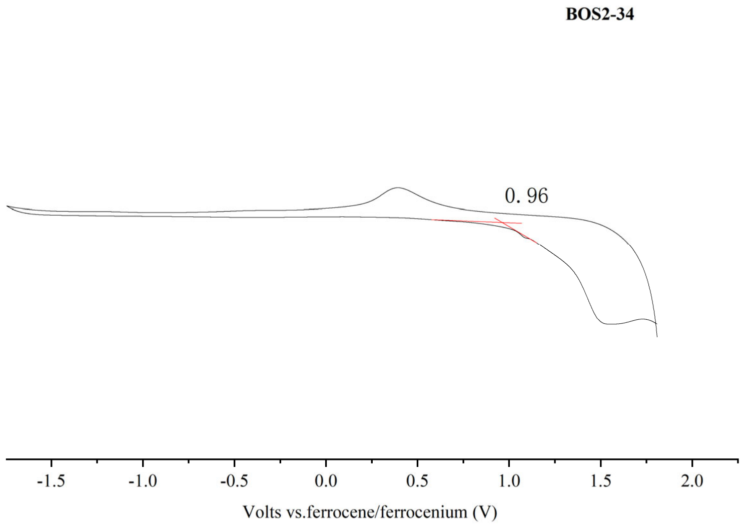

Figure S53. Cyclic voltammogram spectrum of **BOS2-34**

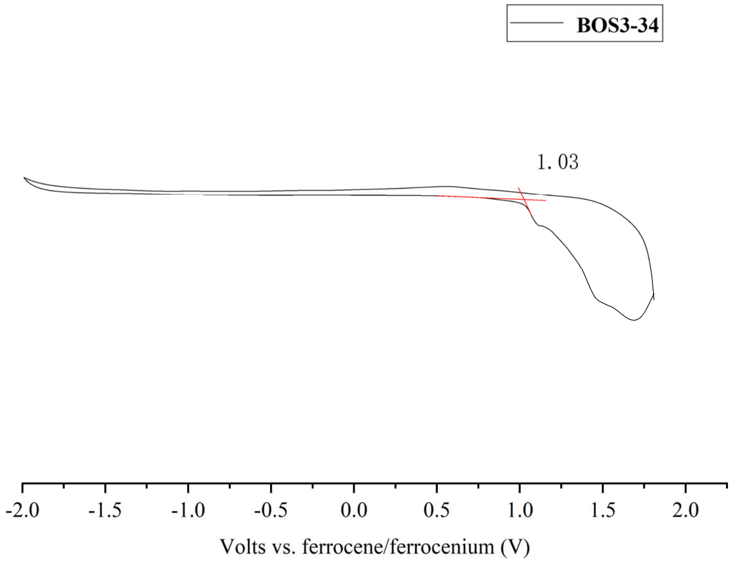

Figure S54. Cyclic voltammogram spectrum of **BOS3-34**

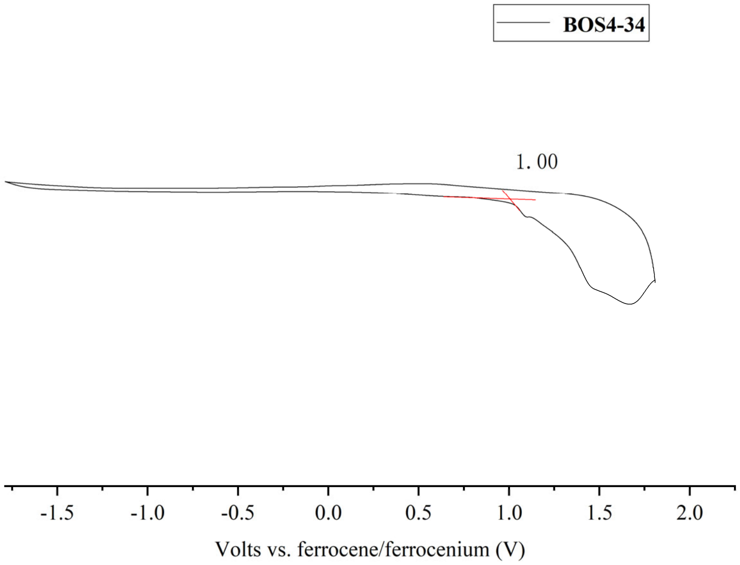

Figure S55. Cyclic voltammogram spectrum of **BOS4-34**

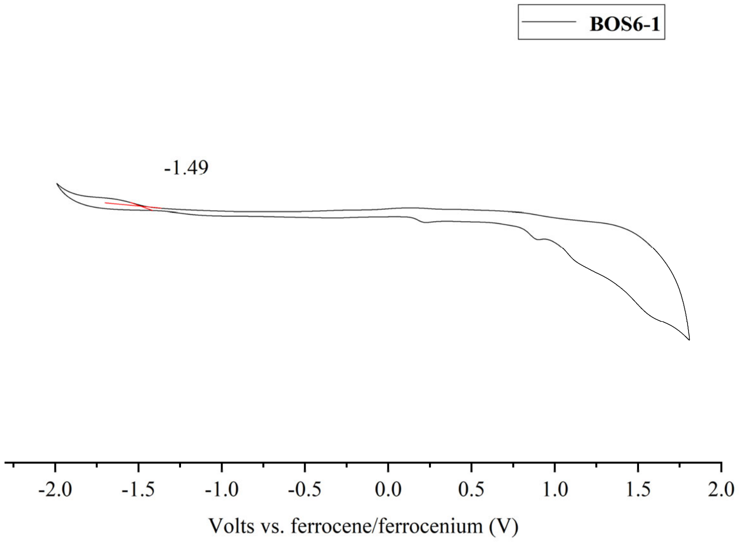

Figure S56. Cyclic voltammogram spectrum of **BOS6-1**

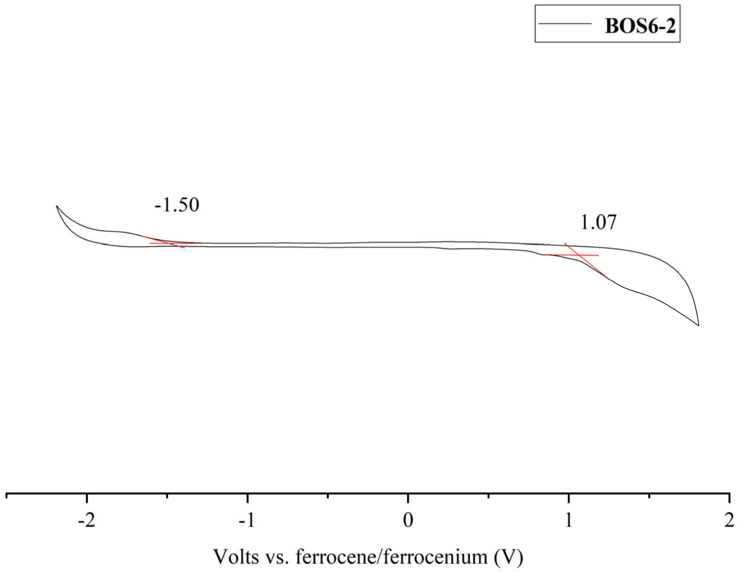

Figure S57. Cyclic voltammogram spectrum of **BOS6-2**

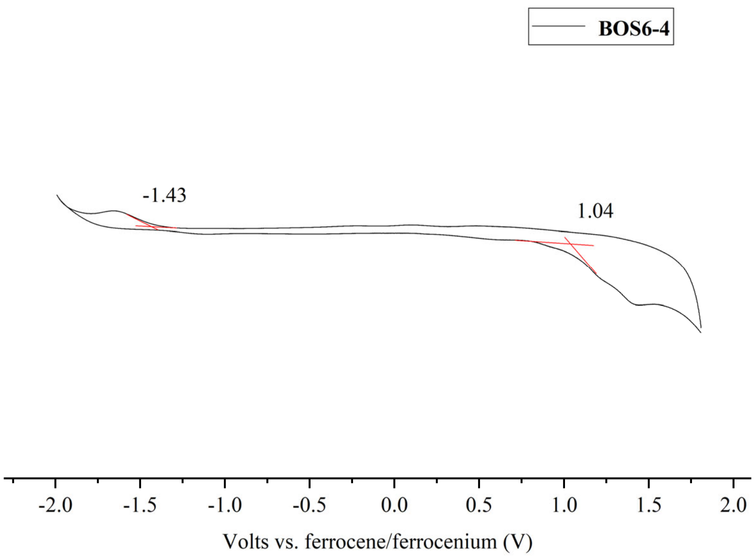

Figure S58. Cyclic voltammogram spectrum of **BOS6-4**

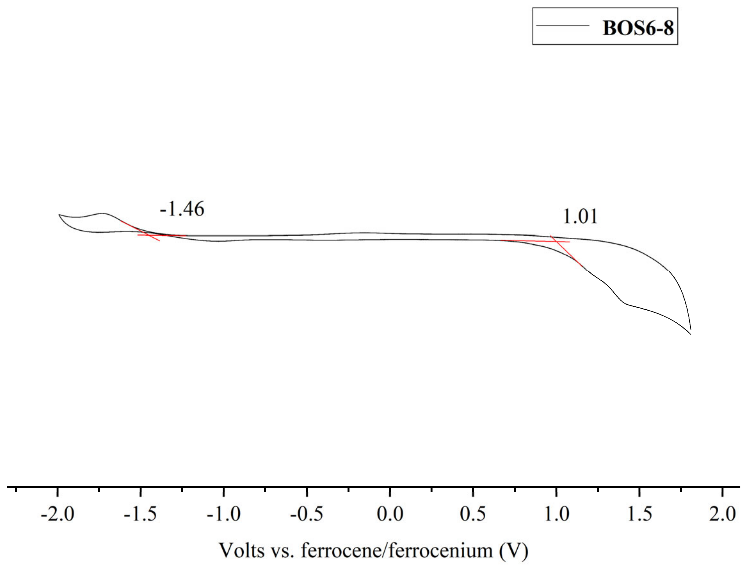

Figure S59. Cyclic voltammogram spectrum of **BOS6-8**

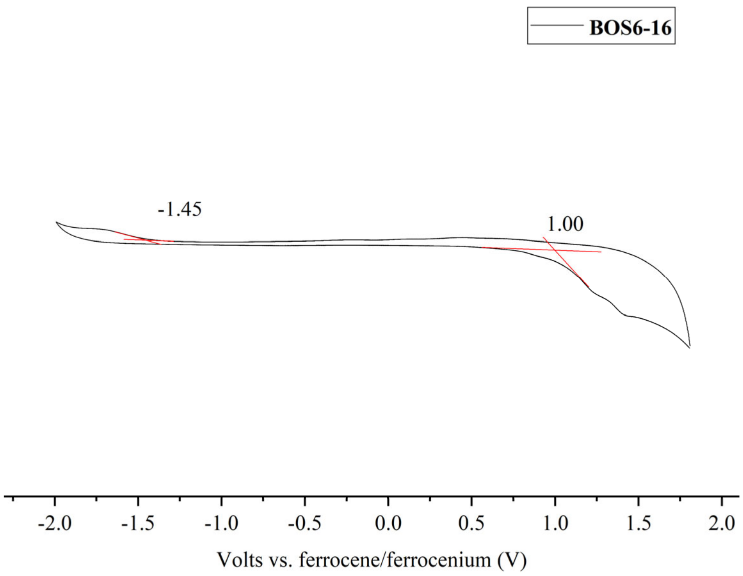

Figure S60. Cyclic voltammogram spectrum of **BOS6-16**

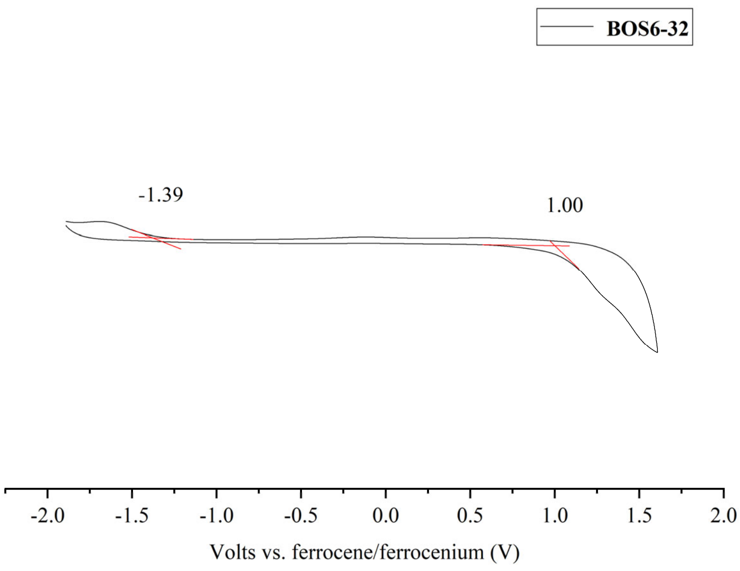

Figure S61. Cyclic voltammogram spectrum of **BOS6-32**

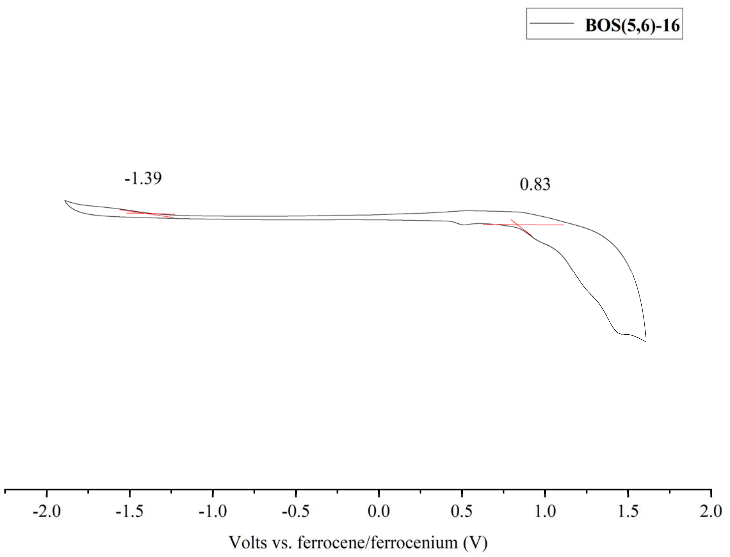

Figure S62. Cyclic voltammogram spectrum of **BOS(5,6)-16**

IX. References

[1] Tobin, J. M.; McCabe, T. J. D.; Prentice, A. W.; Holzer, S.; Lloyd, G. O.; Paterson, M. J.; Arrighi, V.; Cormack, P. A. G.; Vilela, F. Polymer-Supported Photosensitizers for Oxidative Organic Transformations in Flow and under Visible Light Irradiation. *ACS Catal.* **2017**, *7*, 4602.

[2] Yin, J.; Jacobse, P. H.; Pyle, D.; Wang, Z.; Crommie, M. F.; Dong, G. Programmable Fabrication of Monodisperse Gra-phene Nanoribbons via Deterministic Iterative Synthesis. *J. Am. Chem. Soc.* **2022**, *144*, 16012.

[3] Noguchi, H.; Hojo, K.; Suginome, M. Boron-masking Strategy for the Selective Synthesis of Oligoarenes via Iterative Suzuki-Miyaura Coupling. *J. Am. Chem. Soc.* **2007**, *129*, 758.

[4] Liu, S.-J.; Lin, W.-P.; Yi, M.-D.; Xu, W.-J.; Tang, C.; Zhao, Q.; Ye, S.-H.; Liu, X.-M.; Huang, W. Conjugated Polymers with Cationic Iridium(Iii) Complexes in the Side-Chain for Flash Memory Devices Utilizing Switchable through-Space Charge Transfer. *J. Mater. Chem.* **2012**, *22*, 22964.

[5] Ivanov, M. V.; Talipov, M. R.; Boddeda, A.; Abdelwahed, S. H.; Rathore, R. Hückel Theory + Reorganization Energy = Marcus–Hush Theory: Breakdown of the 1/ n Trend in π-Conjugated Poly-p -Phenylene Cation Radicals Is Explained. *J. Phys. Chem. C* **2017**, *121*, 1552.

[6] Rugen-Penkalla, N.; Klapper, M.; Müllen, K. Highly Charged Conjugated Polymers with Polyphenylene Backbones and Poly(Acrylic Acid) Side Chains. *Macromolecules* **2012**, *45*, 2301.

X. NMR spectra

^{1}H NMR of **BBB$_{o3p}$** ($CDCl_3$)

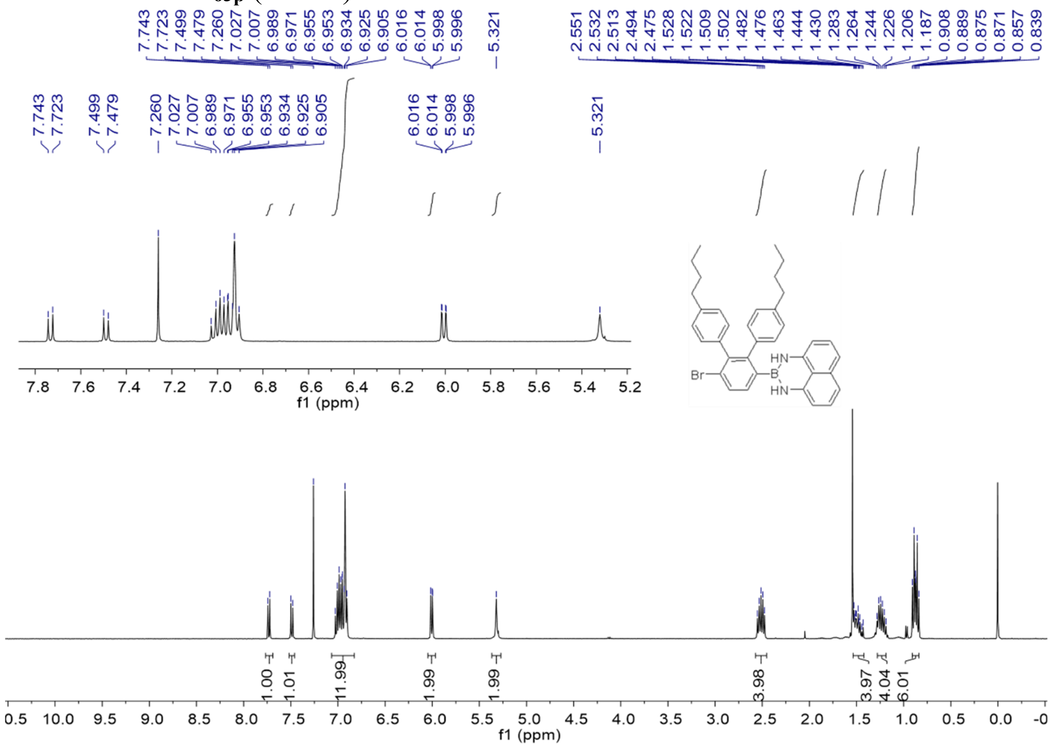

^{13}C NMR of **BBB$_{o3p}$** ($CDCl_3$)

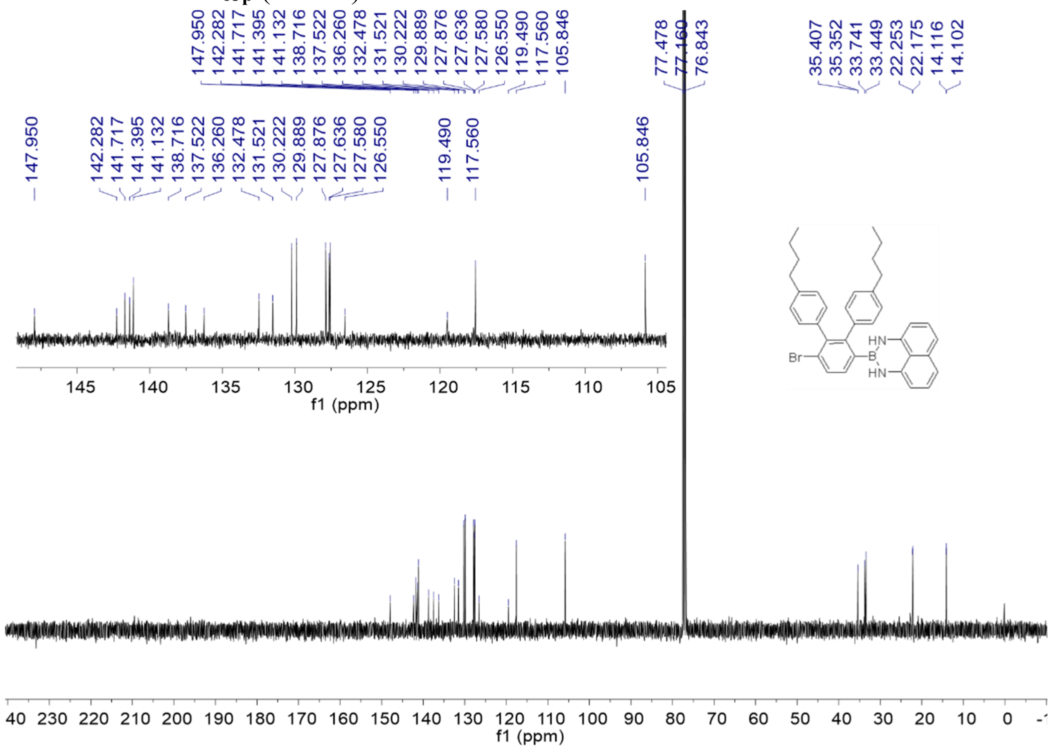

^{11}B NMR of **BBB$_{o3p}$** (CDCl$_3$)

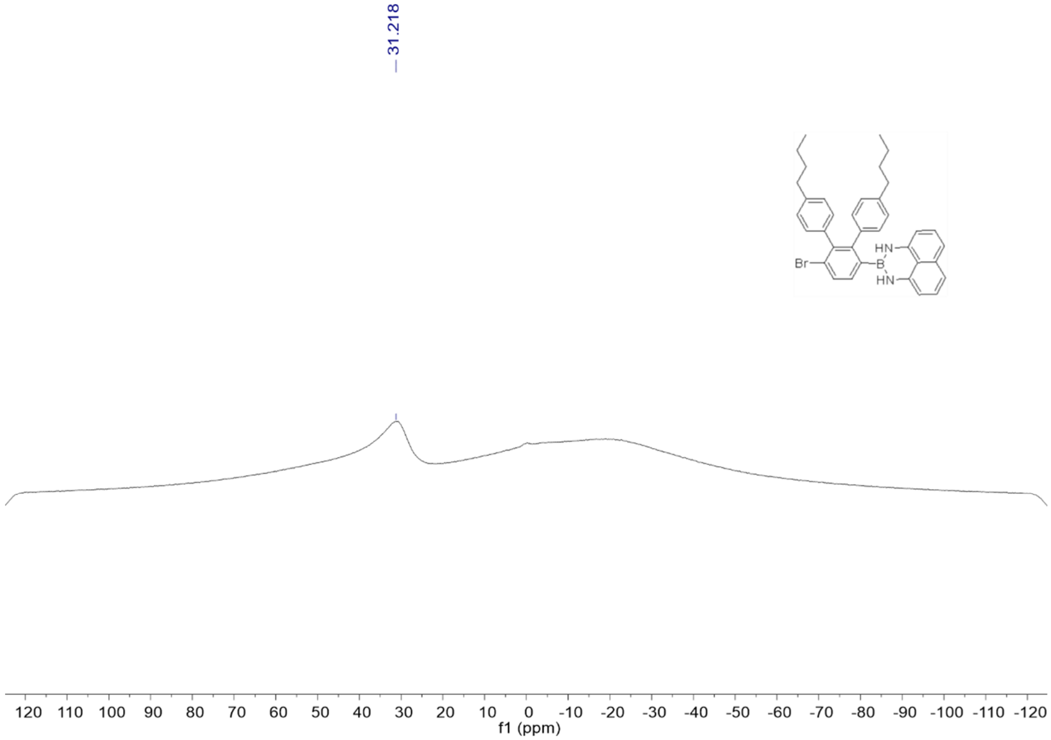

^{1}H NMR of **BOS1-1** (CDCl$_3$)

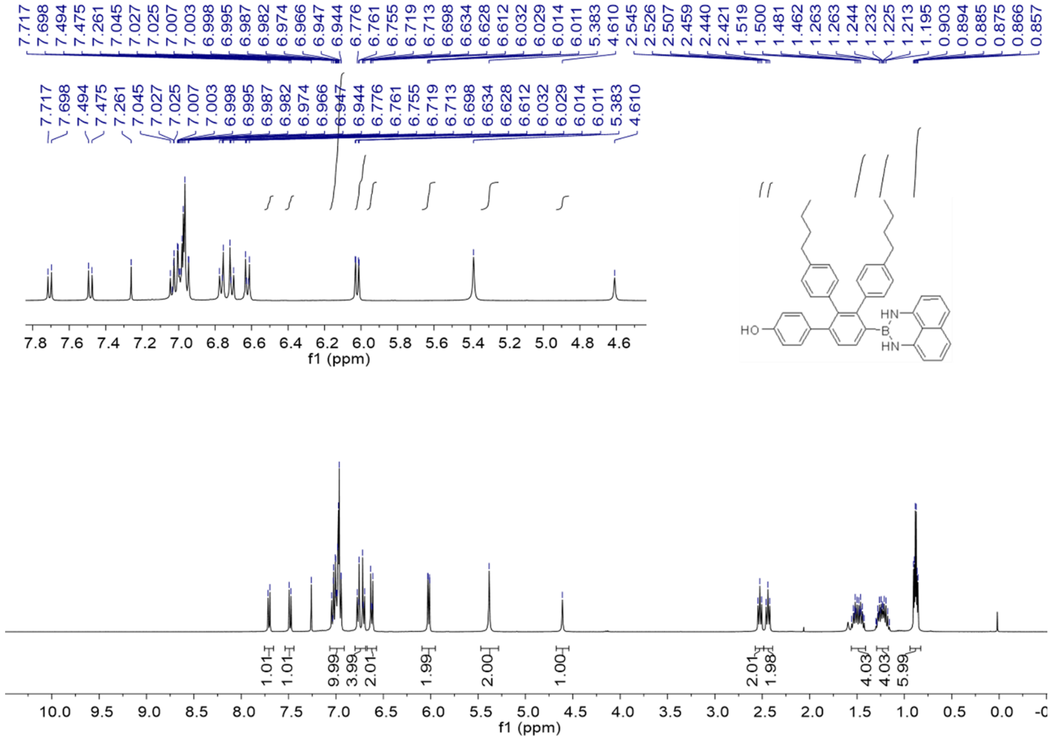

^{13}C NMR of **BOS1-1** ($CDCl_3$)

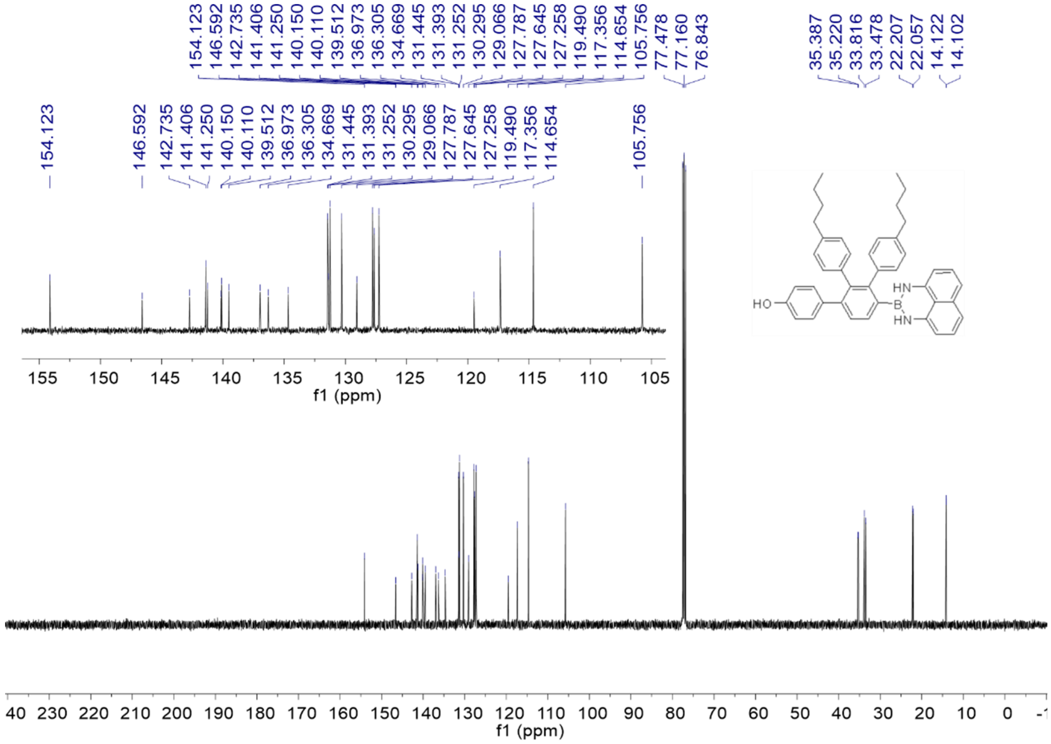

^{11}B NMR of **BOS1-1** ($CDCl_3$)

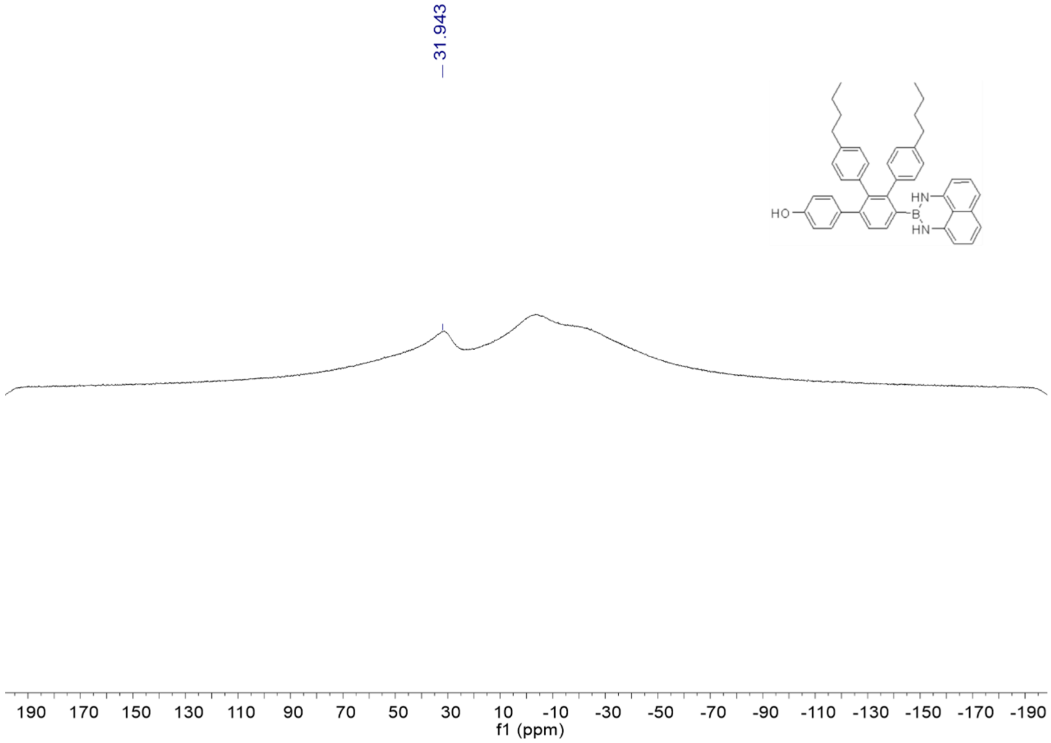

^{1}H NMR of **BOS1-2** ($CDCl_3$)

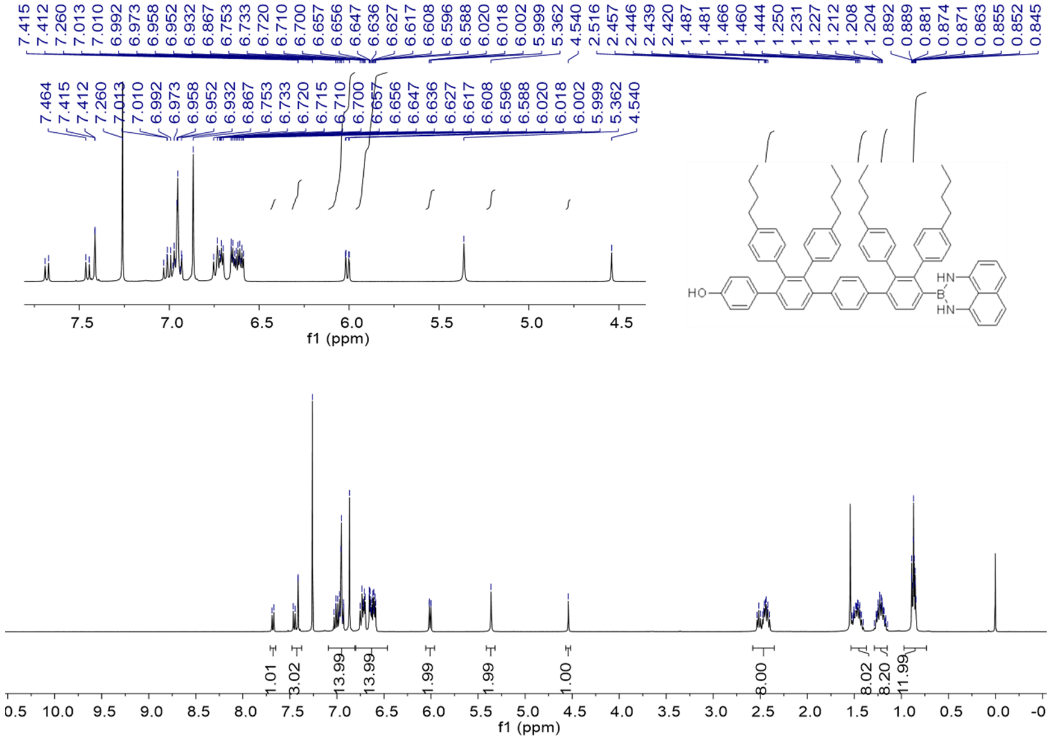

^{11}B NMR of **BOS1-2** ($CDCl_3$)

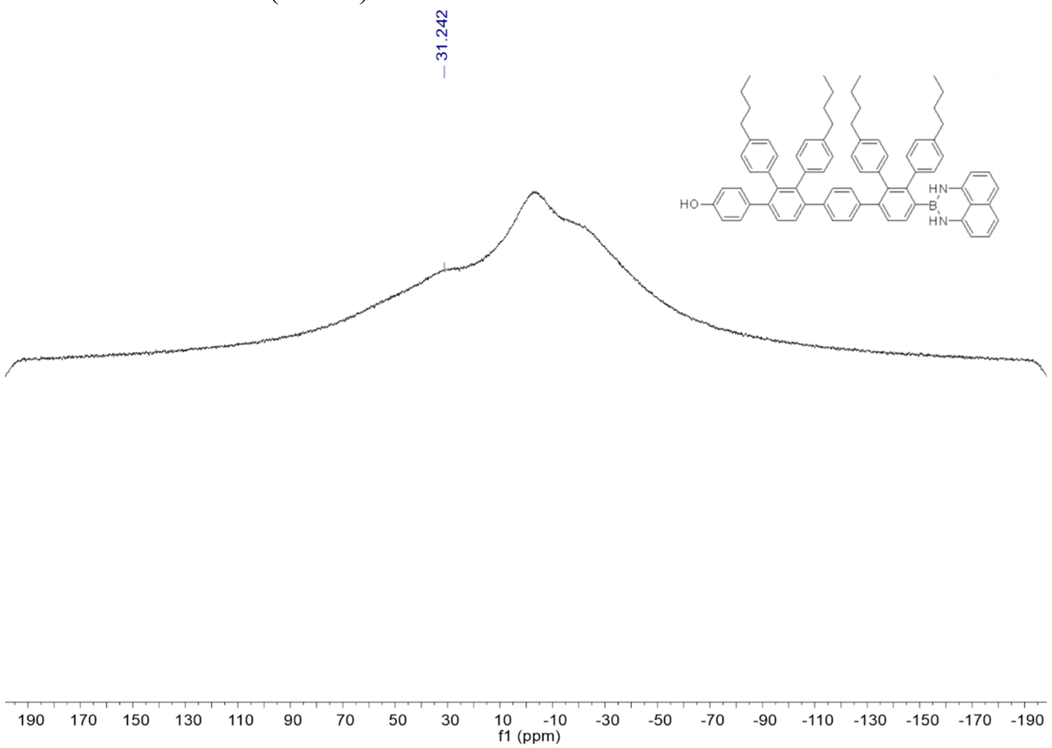

^{1}H NMR of **BOS1-4** ($CDCl_3$)

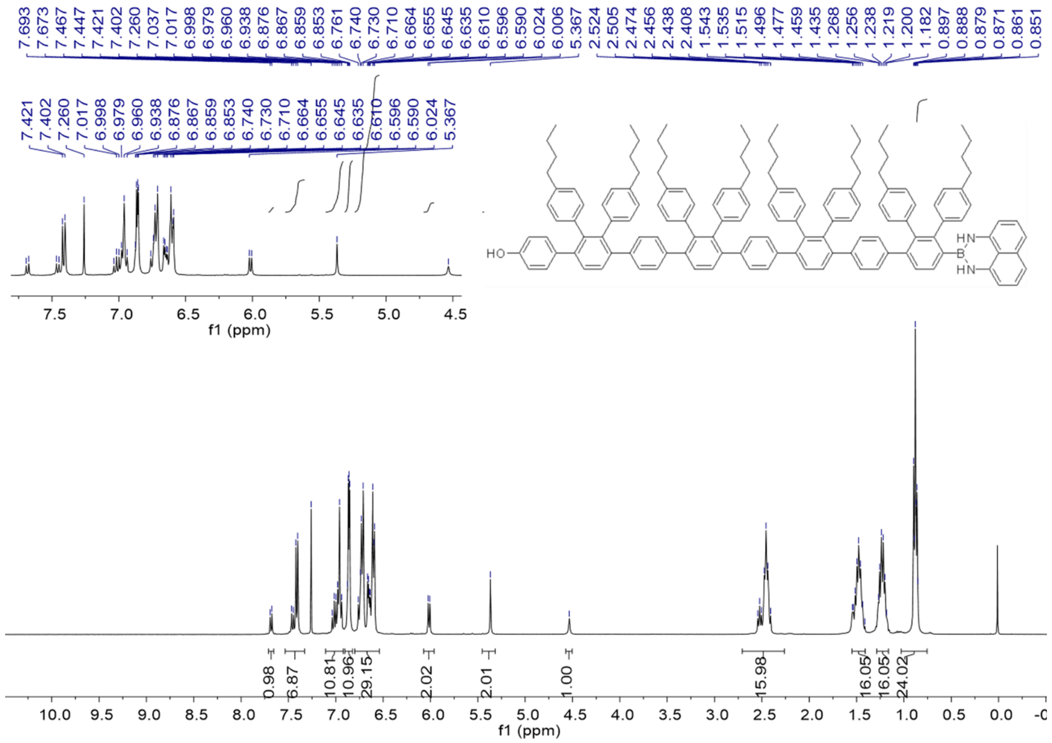

^{11}B NMR of **BOS1-4** ($CDCl_3$)

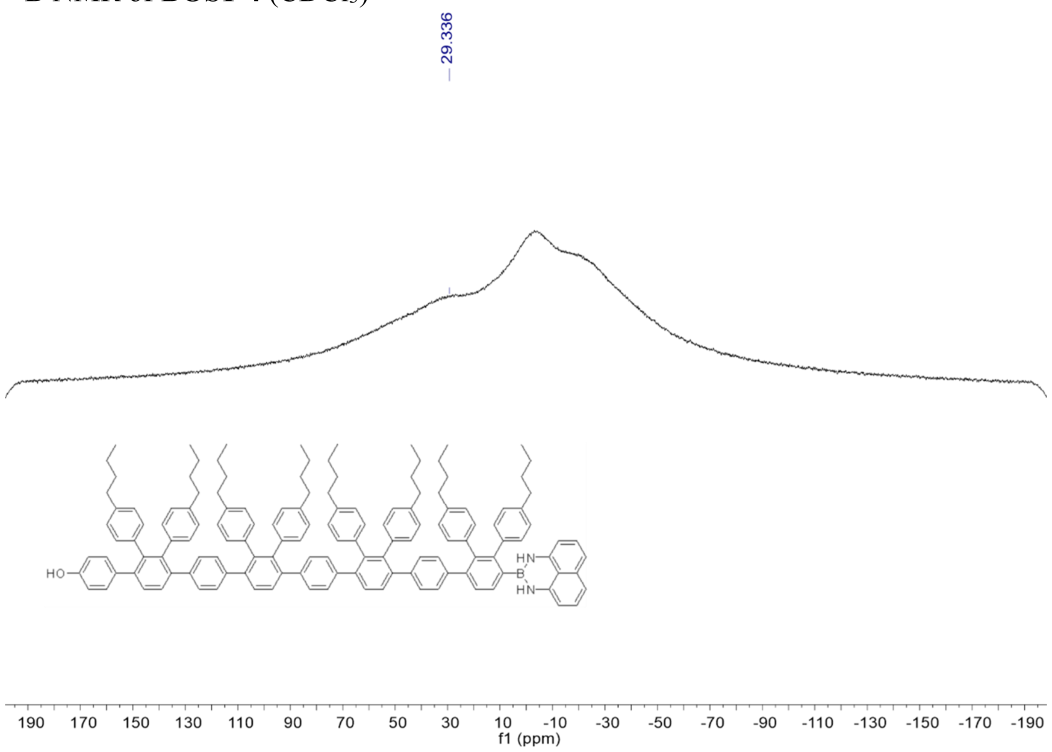

^{1}H NMR of **BOS1-8** ($CDCl_3$)

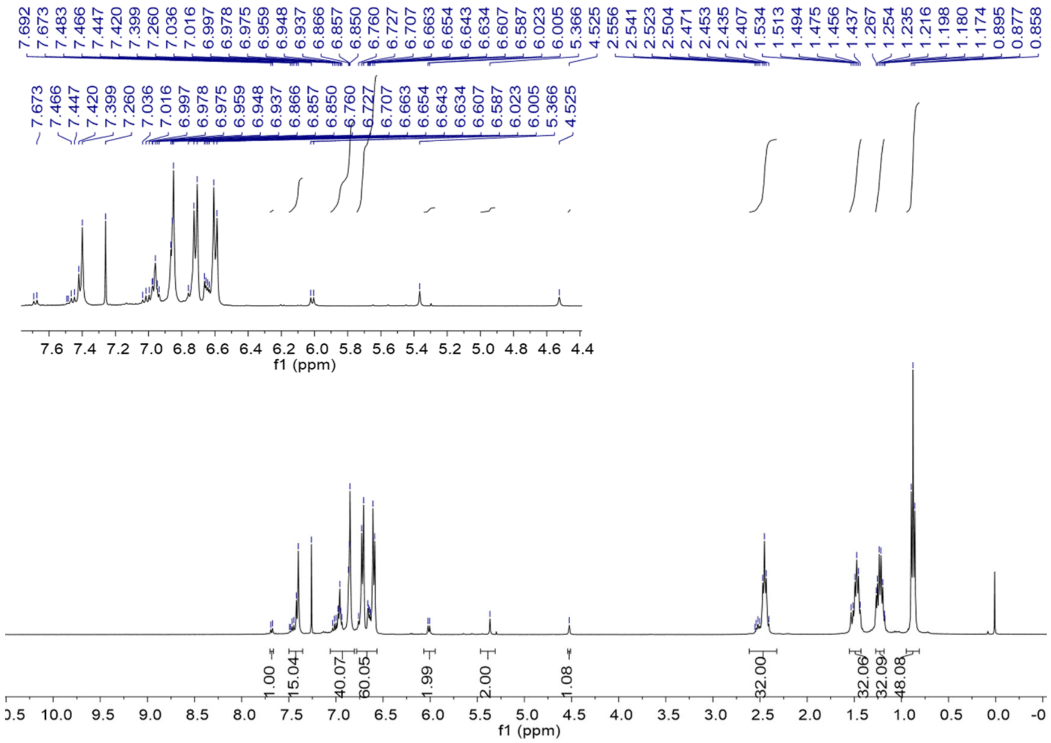

^{11}B NMR of **BOS1-8** ($CDCl_3$)

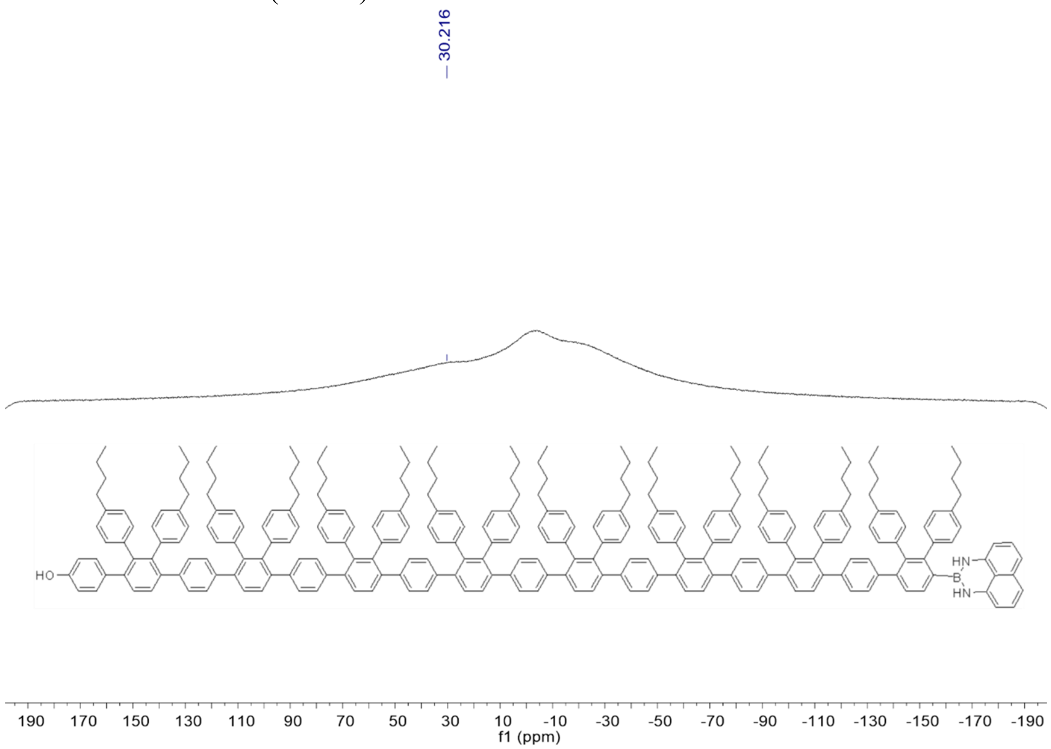

^{1}H NMR of **BOS1-16** ($CDCl_3$)

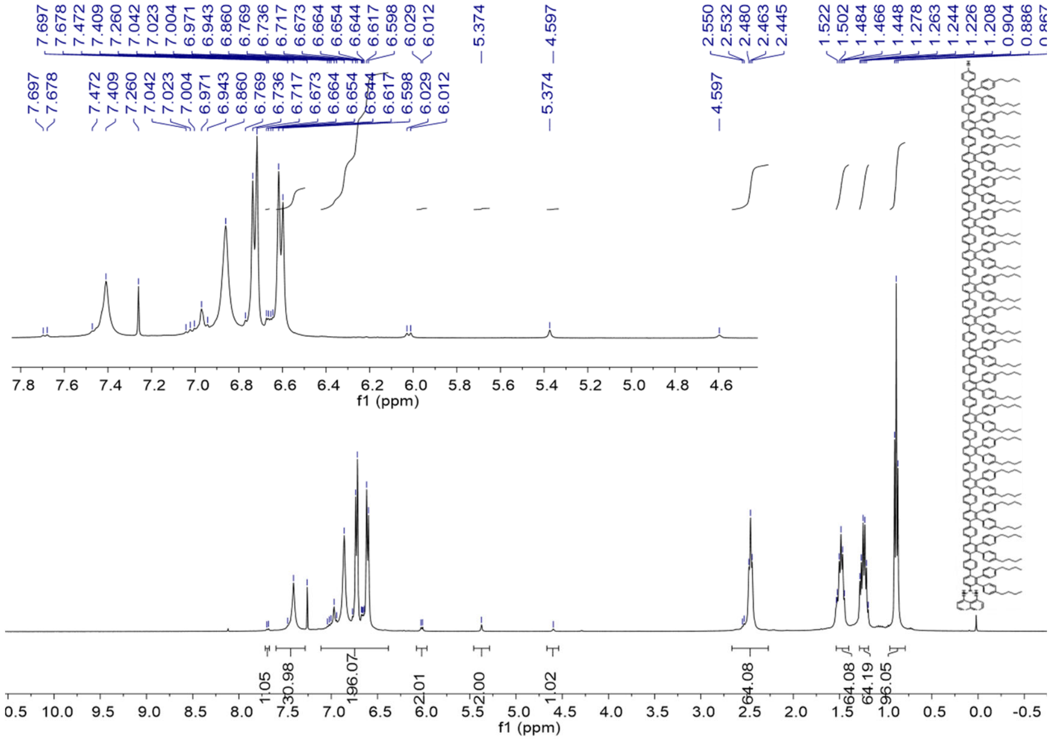

^{11}B NMR of **BOS1-16** ($CDCl_3$)

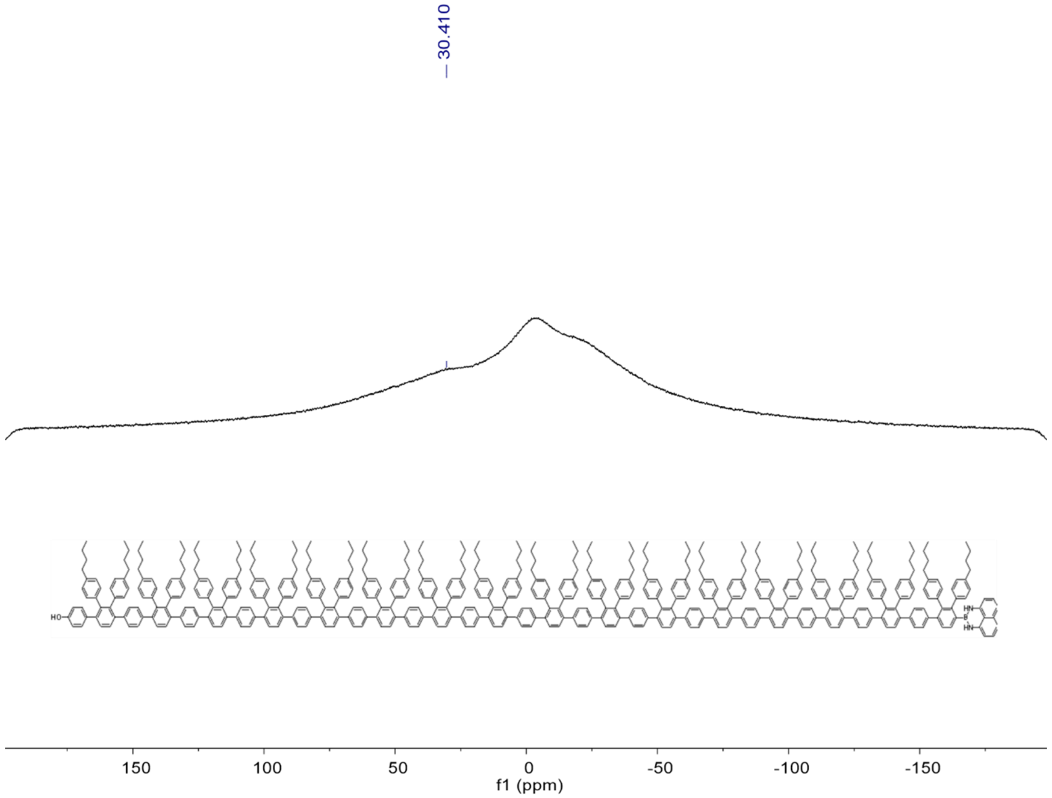

^{1}H NMR of **BOS2-9** ($CDCl_3$)

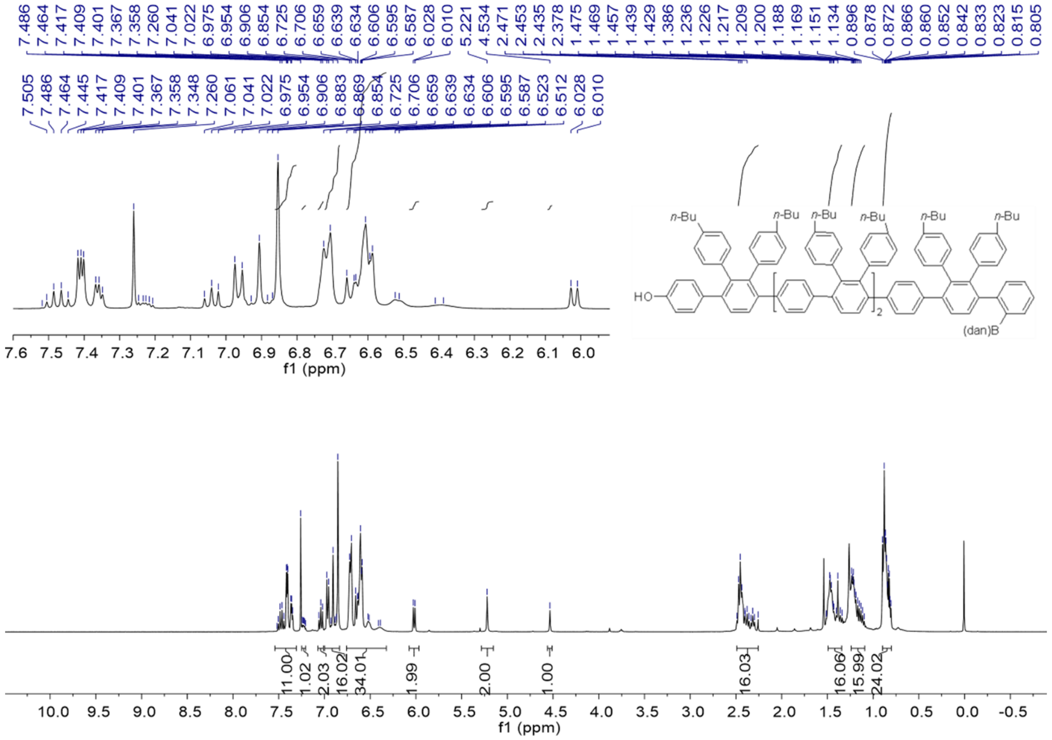

^{1}H NMR of **BOS2-17** ($CDCl_3$)

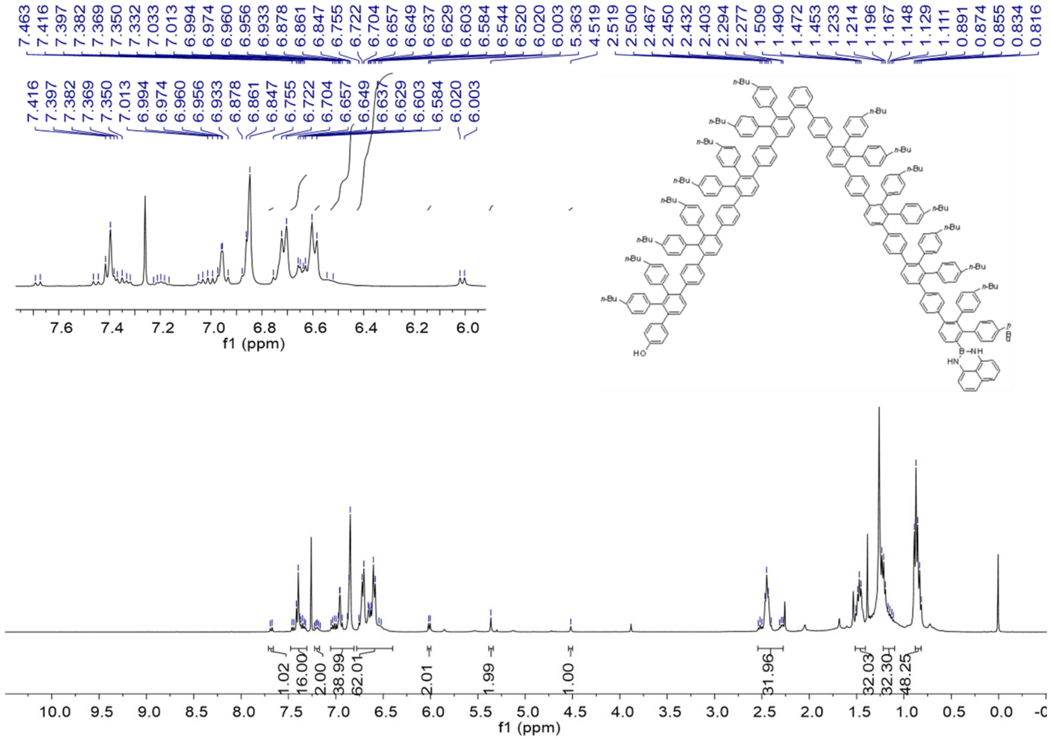

^{1}H NMR of **BOS2-34** ($CDCl_3$)

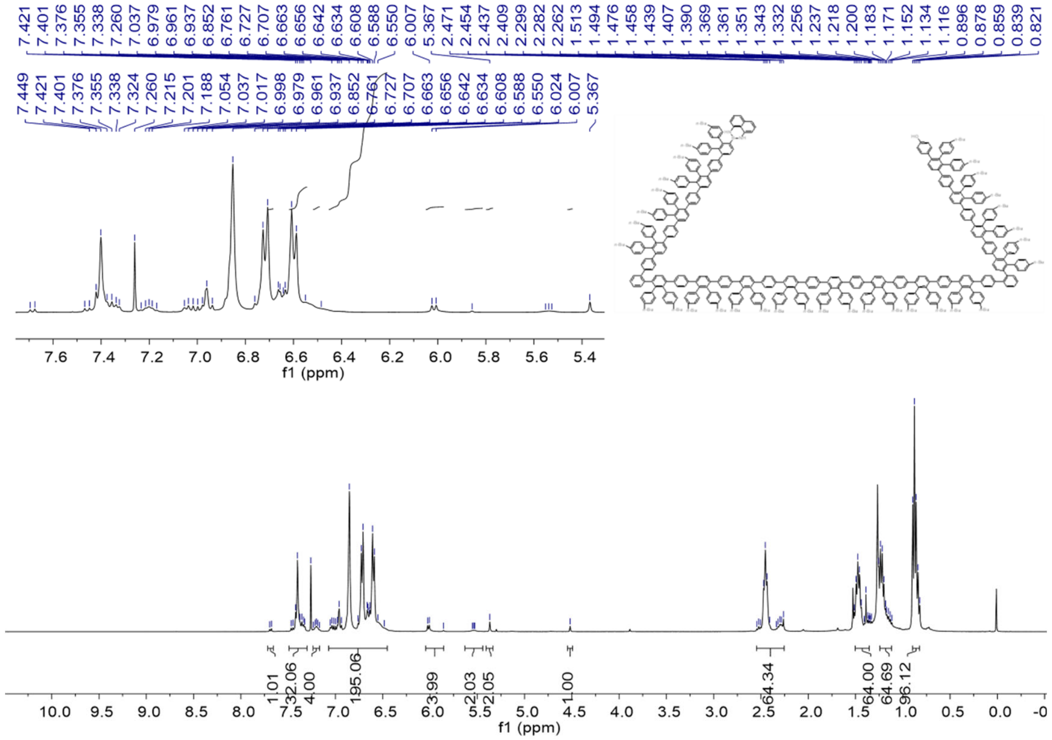

^{1}H NMR of **BOS2-68** ($CDCl_3$)

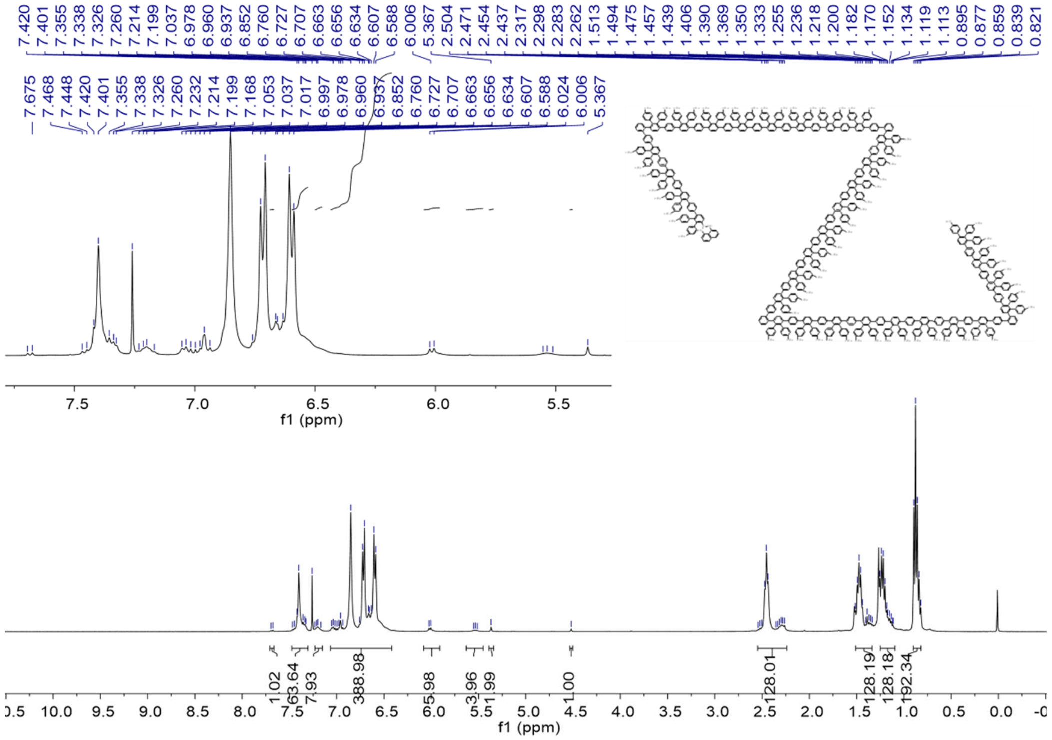

^{1}H NMR of **BOS2-136** ($CDCl_3$)

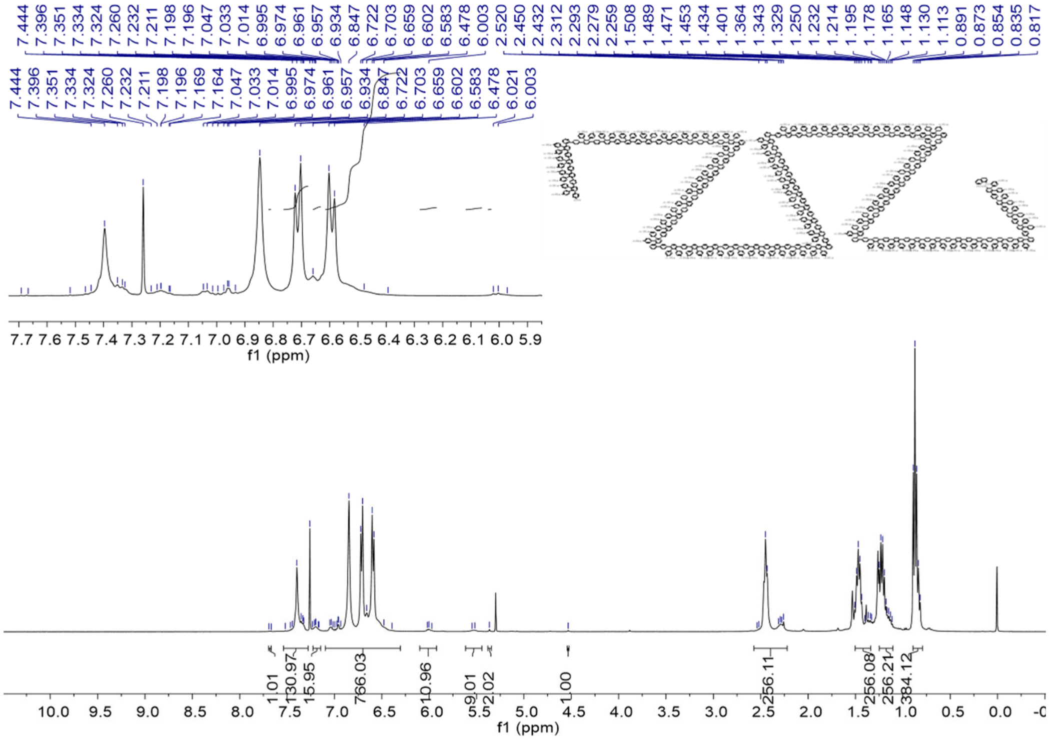

^{1}H NMR of **BOS3-9** ($CDCl_3$)

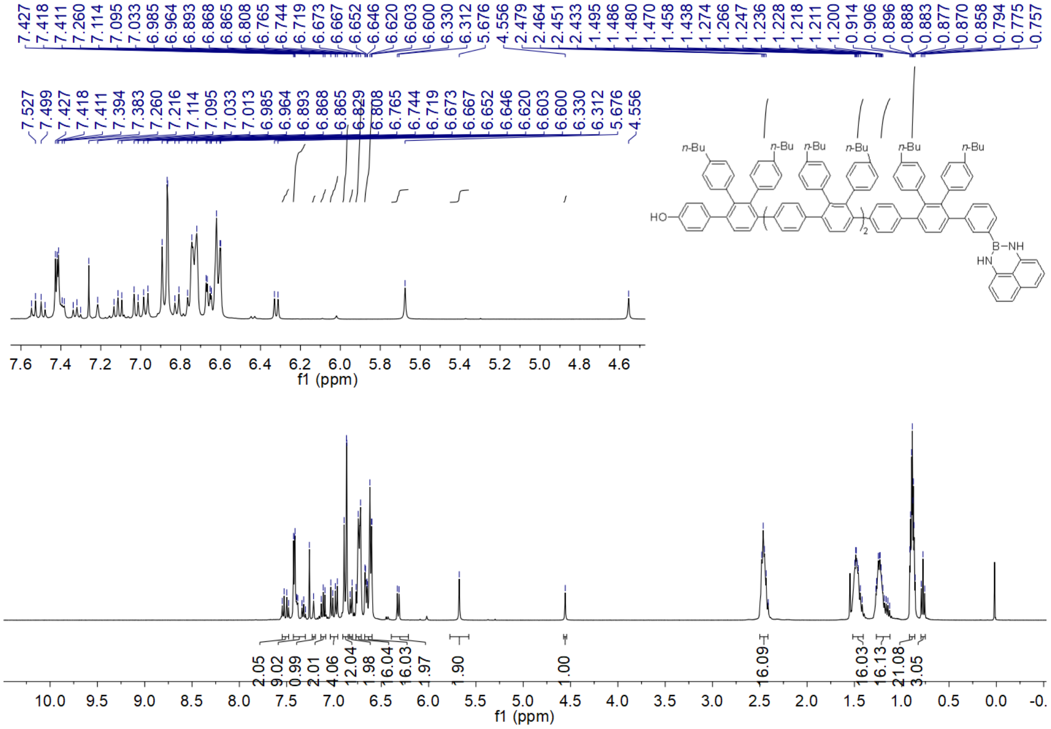

^{1}H NMR of **BOS3-17** ($CDCl_3$)

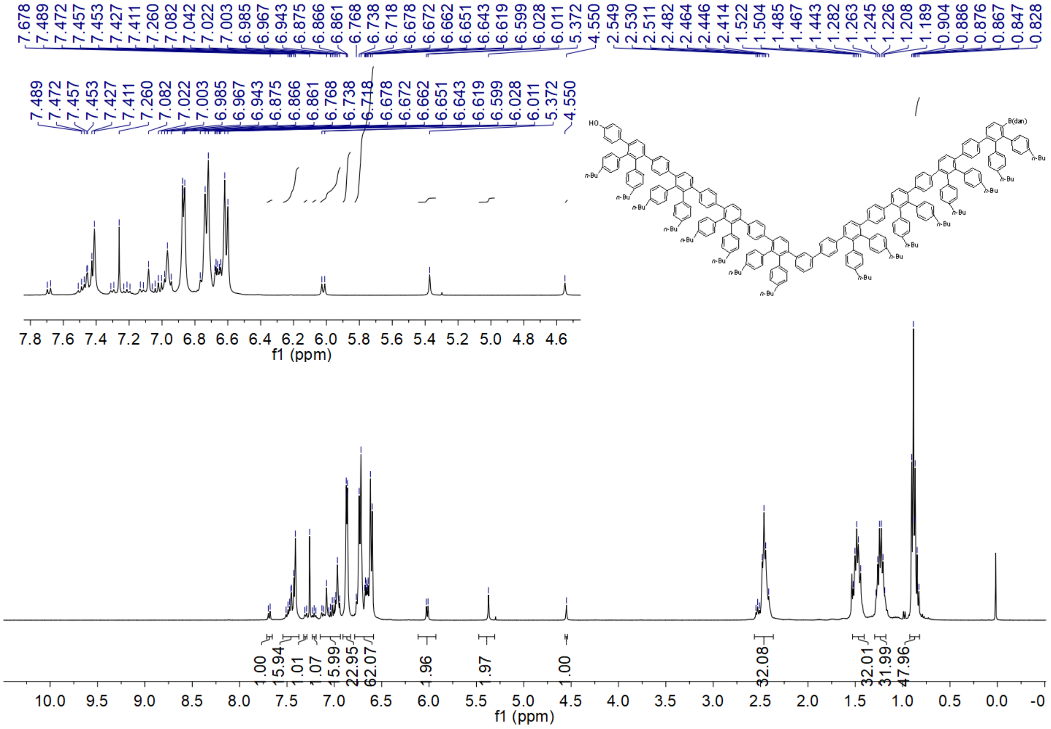

^{1}H NMR of **BOS3-34** ($CDCl_3$)

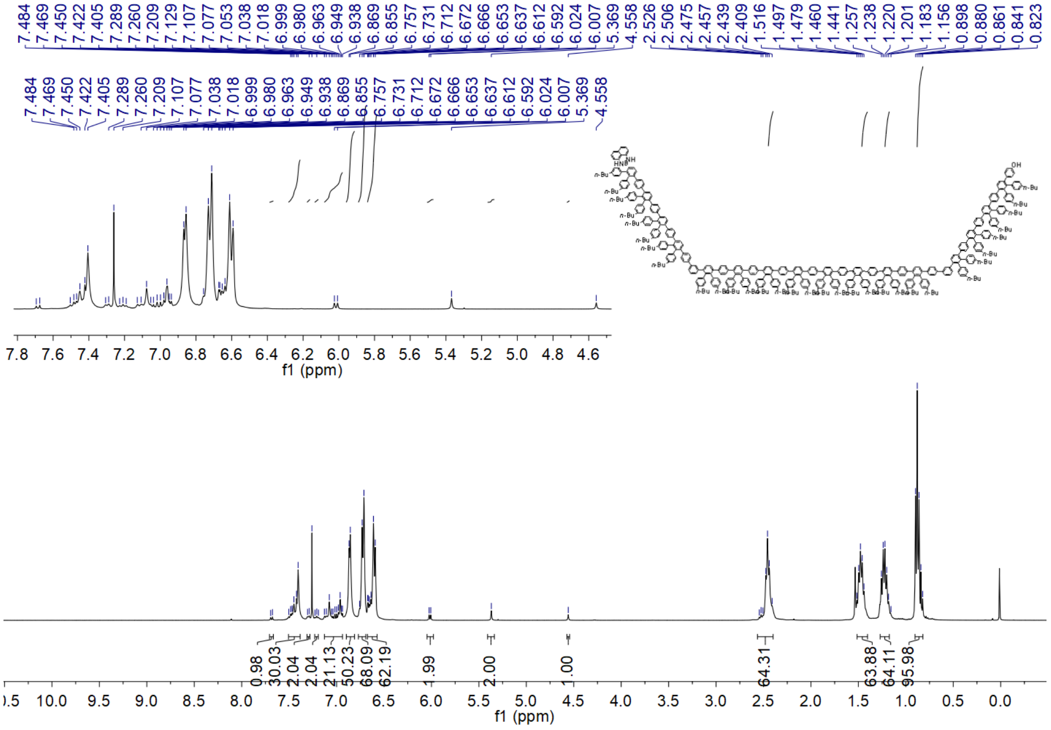

^{1}H NMR of **BOS4-34** ($CDCl_3$)

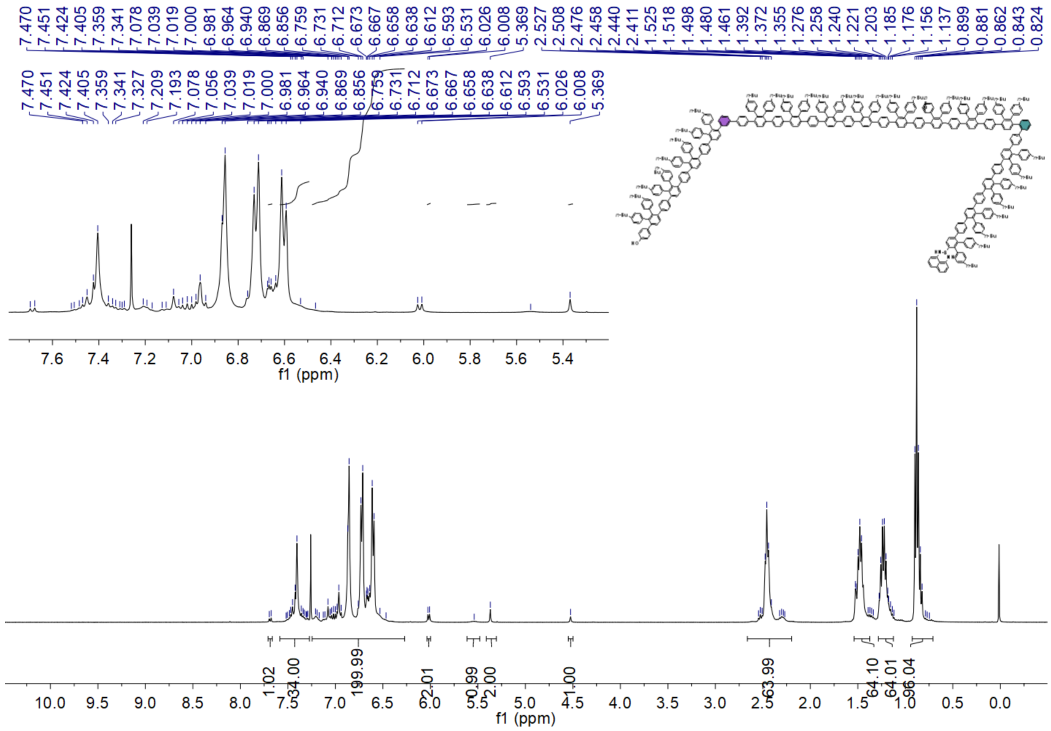

^{1}H NMR of **BOS4-68**($CDCl_3$)

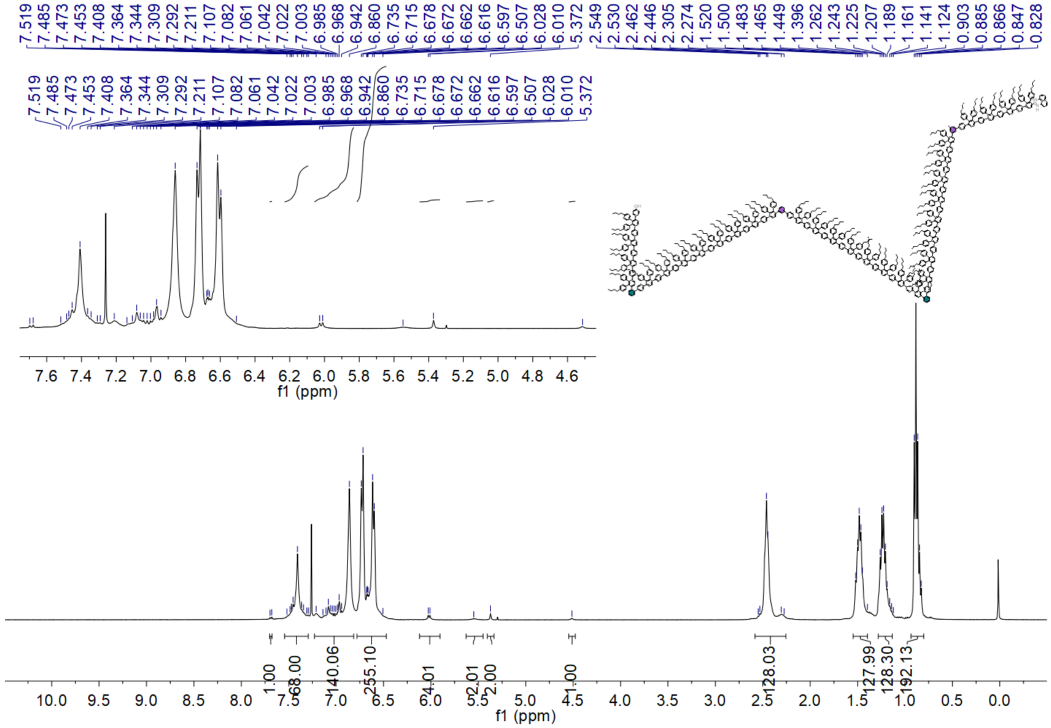

^{1}H NMR of **BOS4-136**($CDCl_3$)

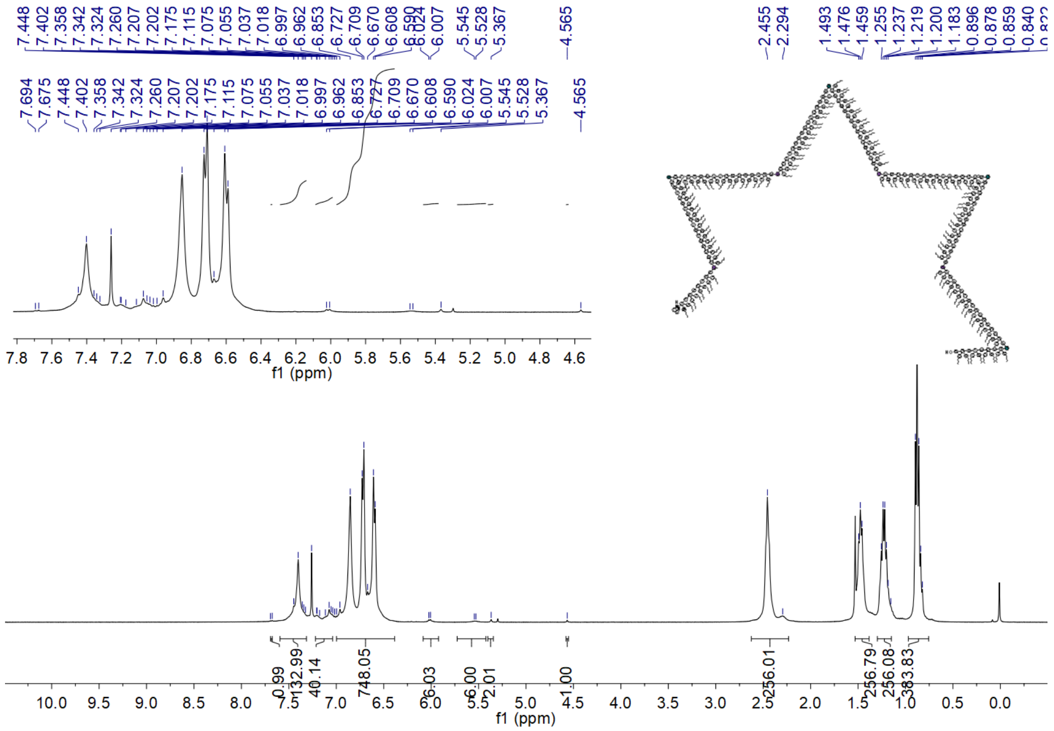

^{1}H NMR of **BBB$_{carbazole}$-S1** ($CDCl_3$)

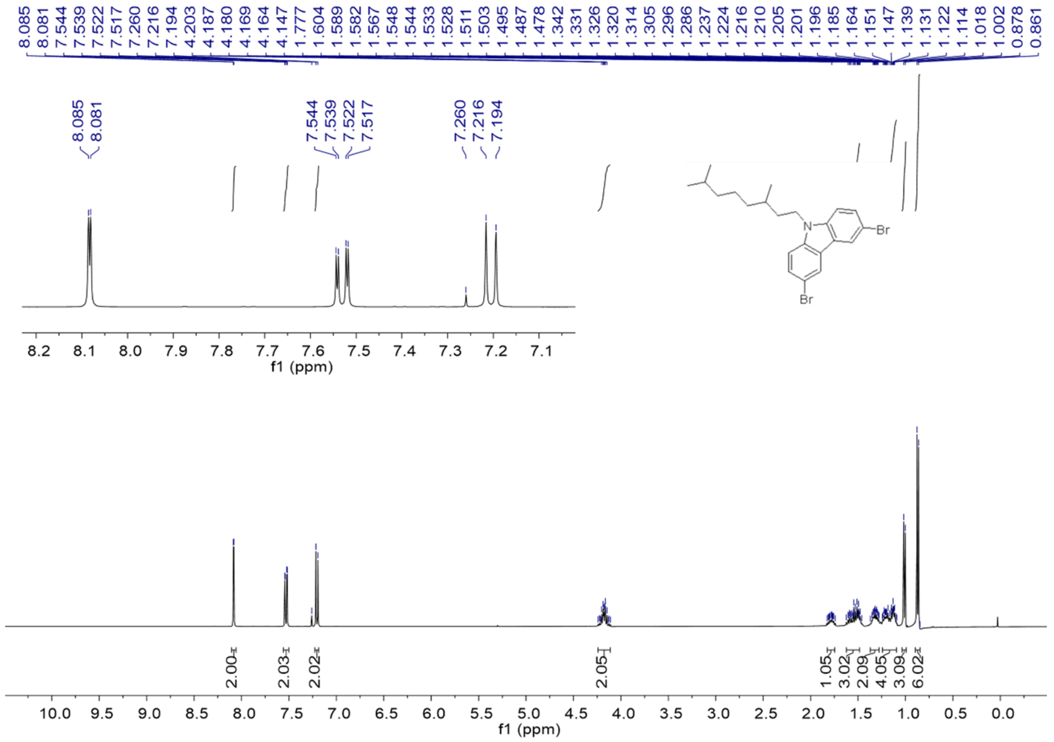

^{13}C NMR of **BBB$_{carbazole}$-S1** ($CDCl_3$)

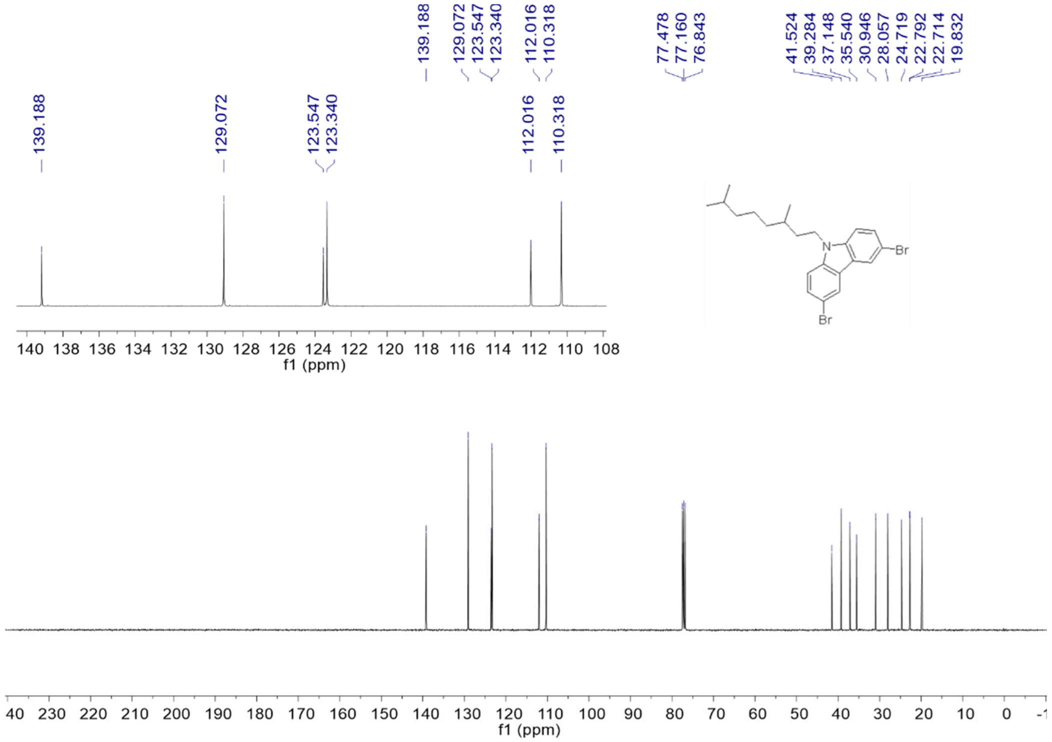

^{1}H NMR of **BBB$_{carbazole}$** ($CDCl_3$)

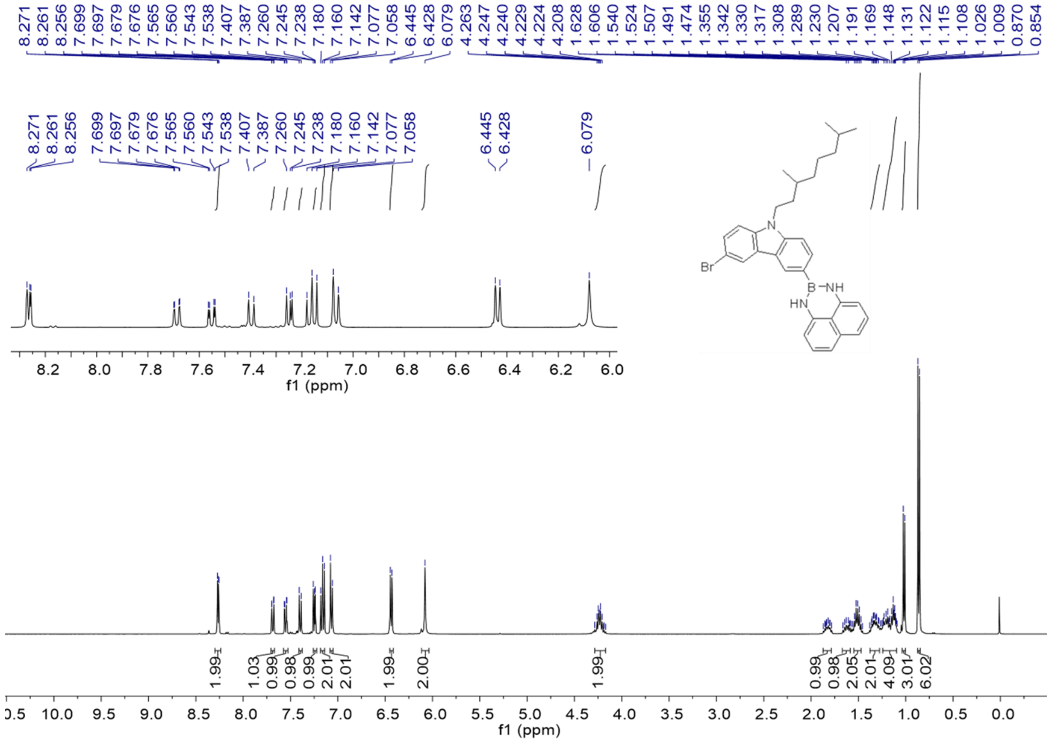

^{13}C NMR of **BBB$_{carbazole}$** ($CDCl_3$)

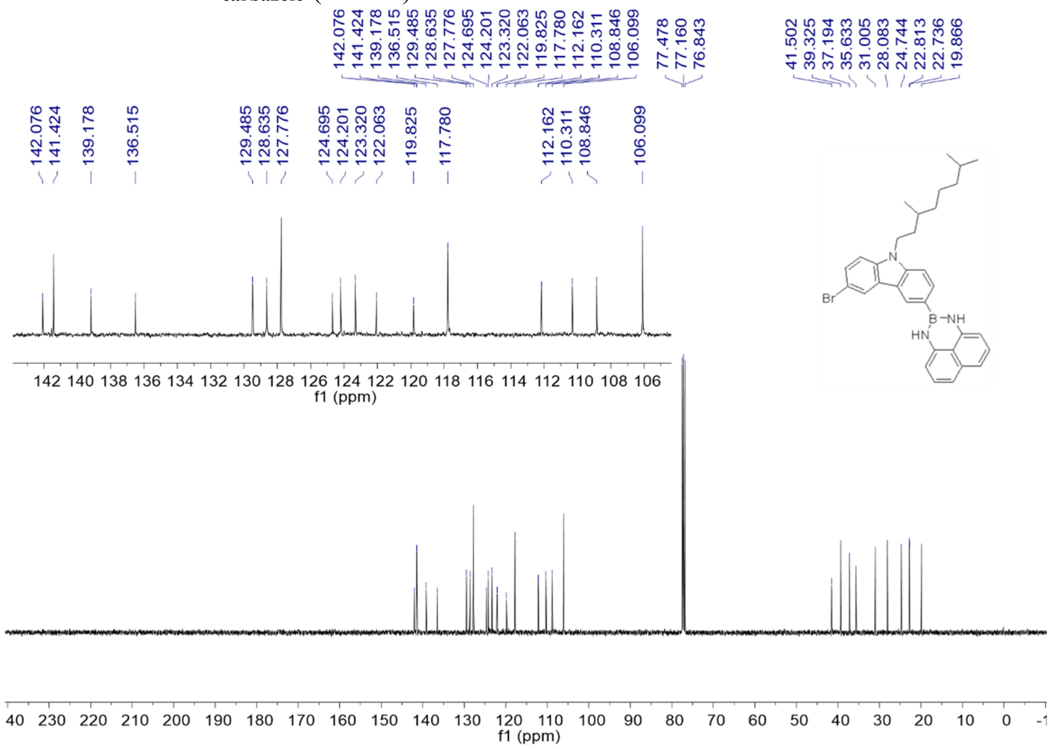

^{11}B NMR of **BBB$_{carbazole}$** ($CDCl_3$)

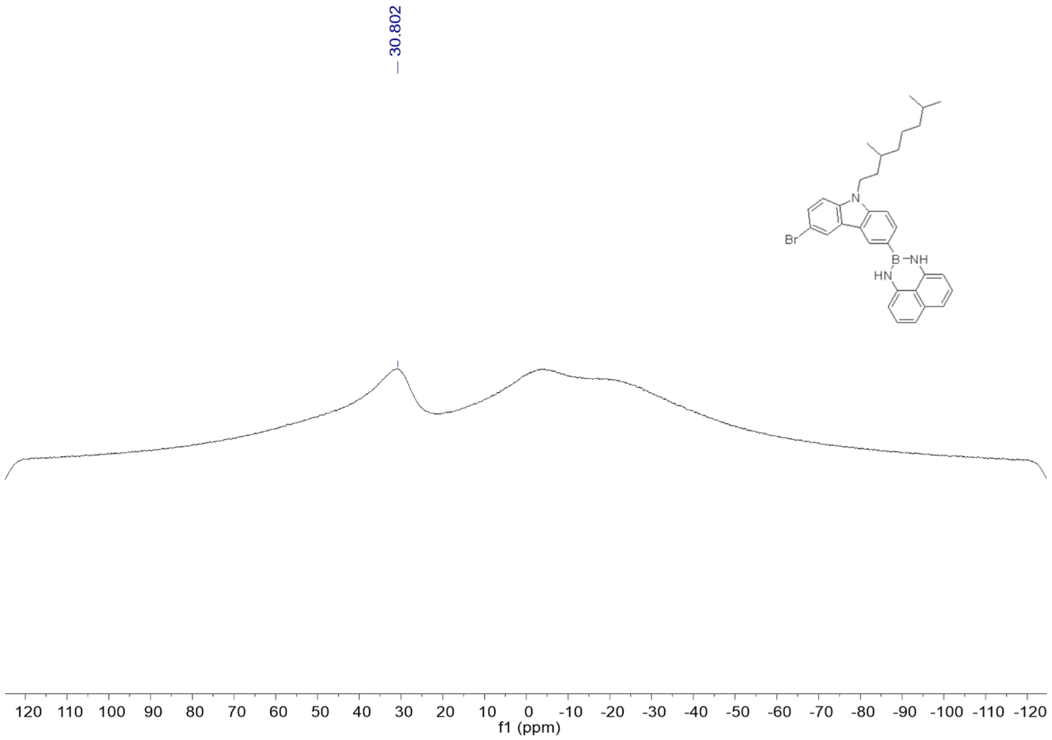

^{1}H NMR of **BBB$_{carbazole}$-2** ($CDCl_3$)

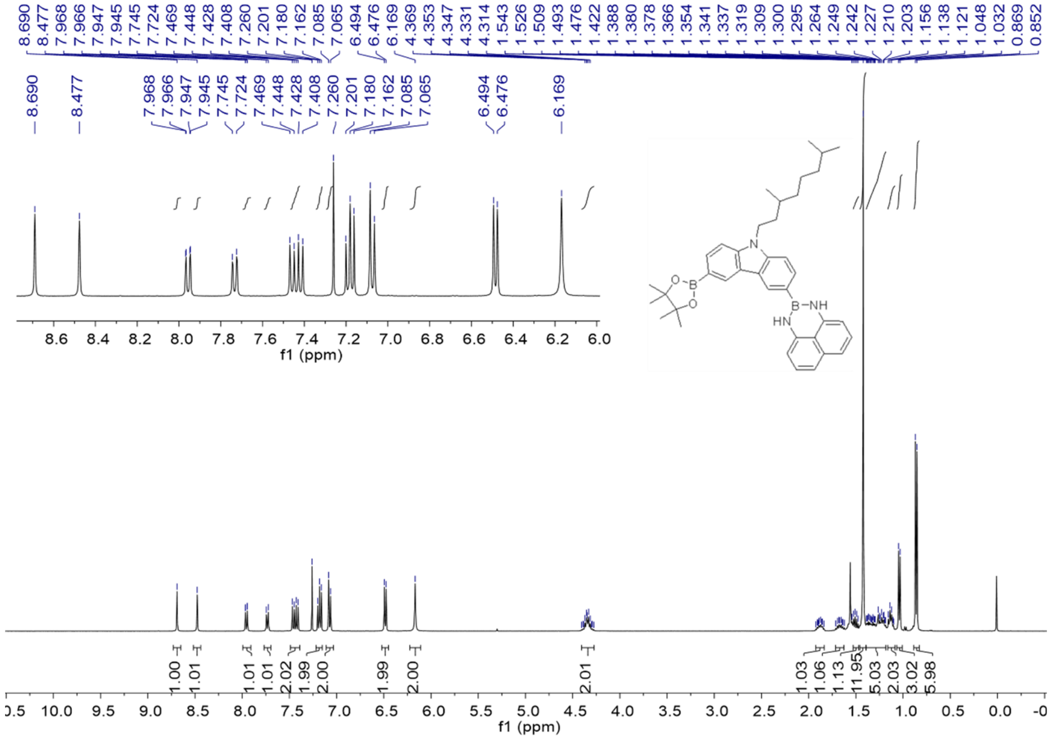

^{13}C NMR of **BBB$_{carbazole}$-2** ($CDCl_3$)

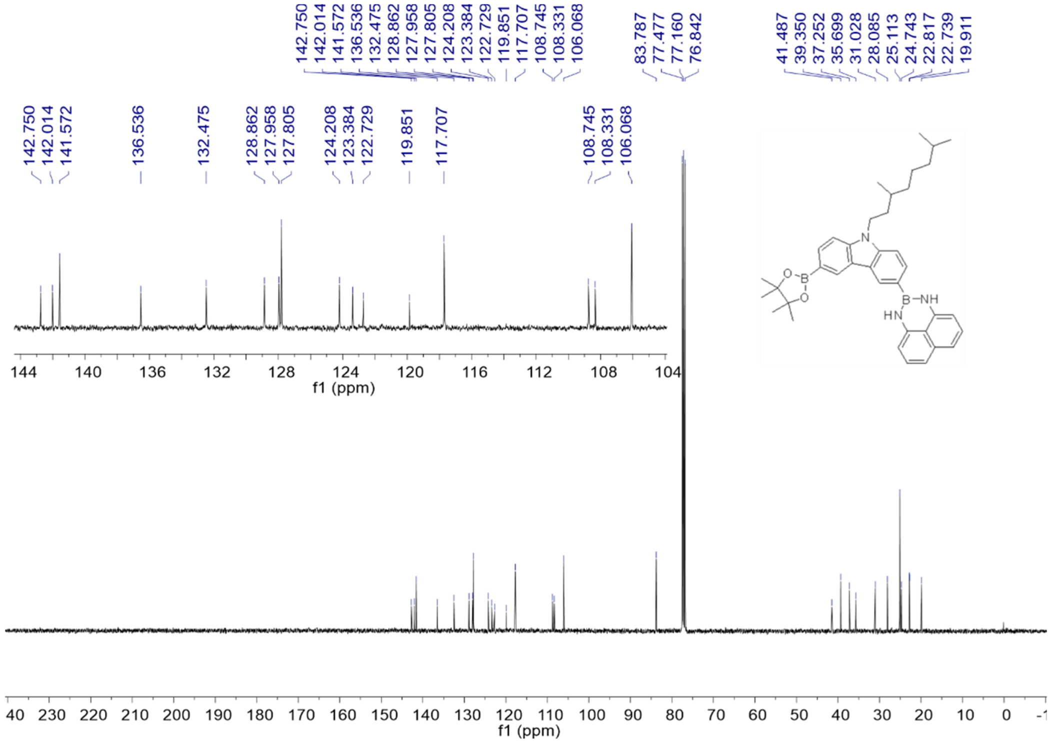

^{11}B NMR of **BBB$_{carbazole}$-2** ($CDCl_3$)

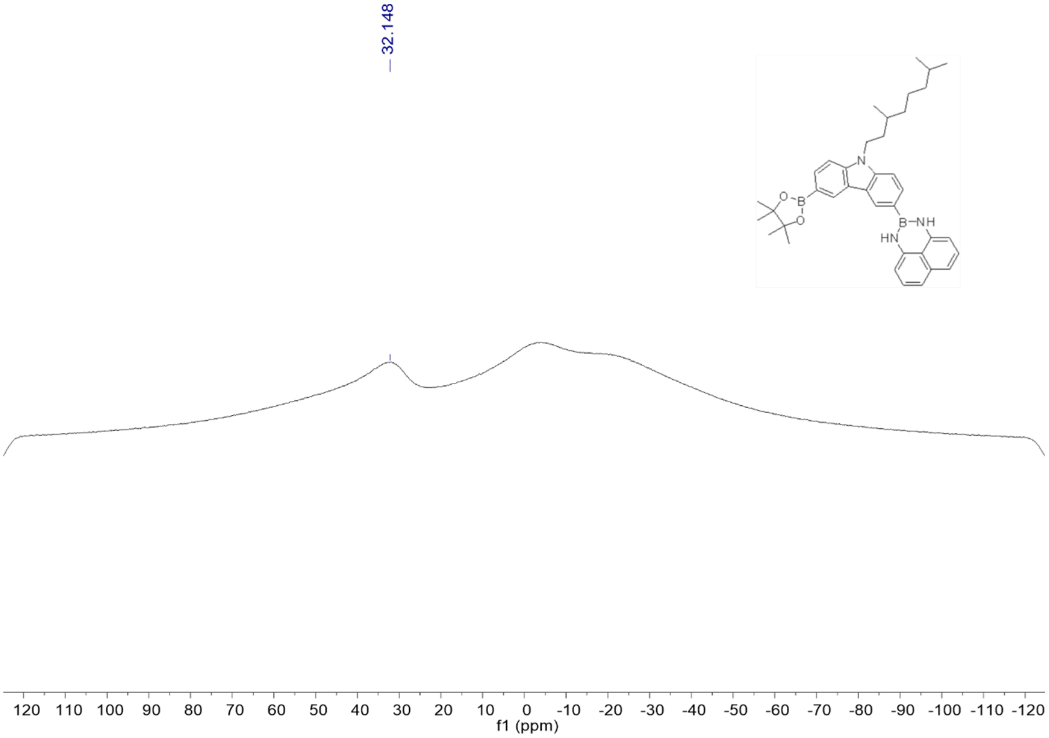

^{1}H NMR of **BBB$_{fluorene}$-S1** ($CDCl_3$)

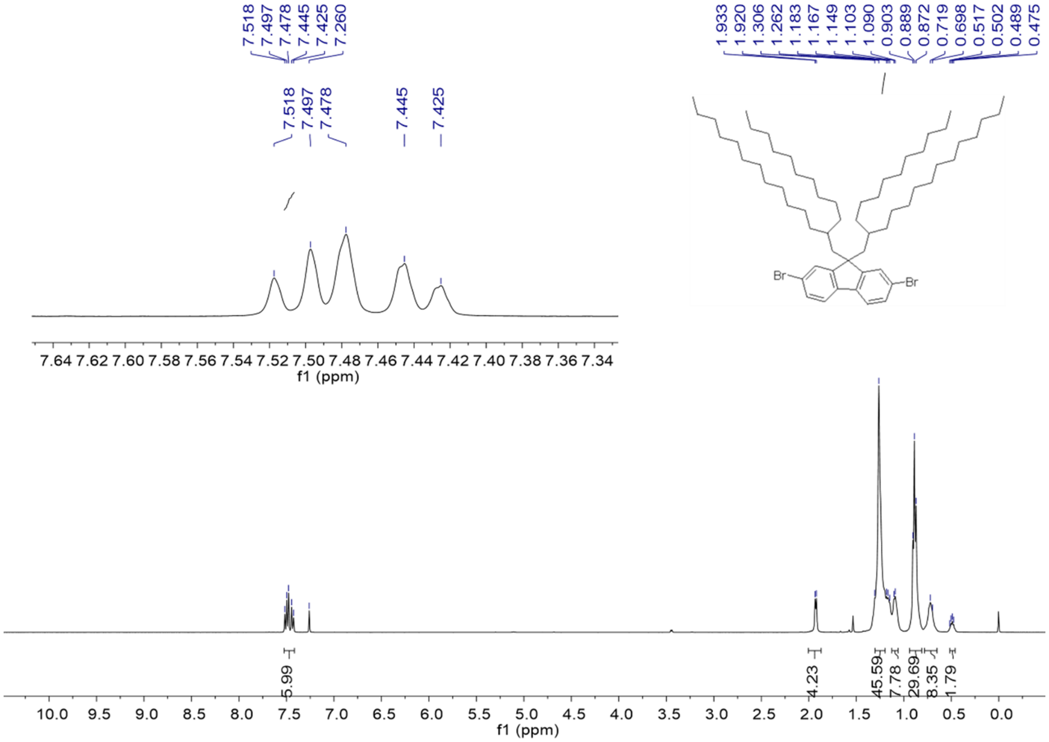

^{13}C NMR of **BBB$_{fluorene}$-S1** ($CDCl_3$)

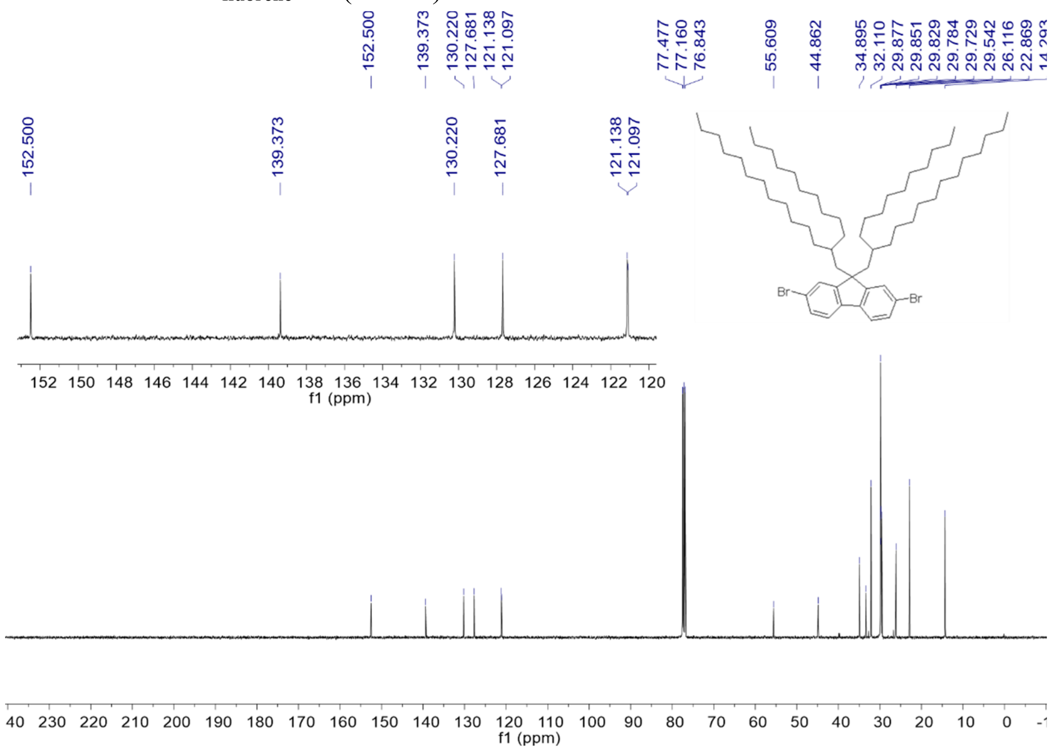

^{1}H NMR of **BBB$_{fluorene}$** ($CDCl_3$)

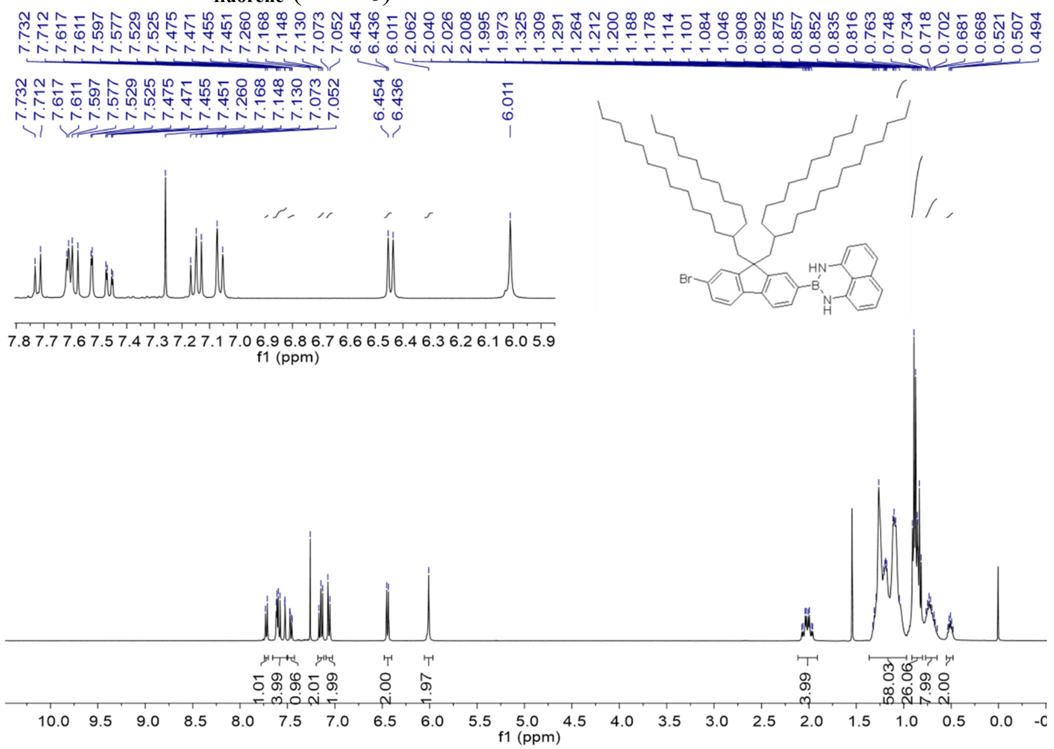

^{13}C NMR of **BBB$_{fluorene}$** ($CDCl_3$)

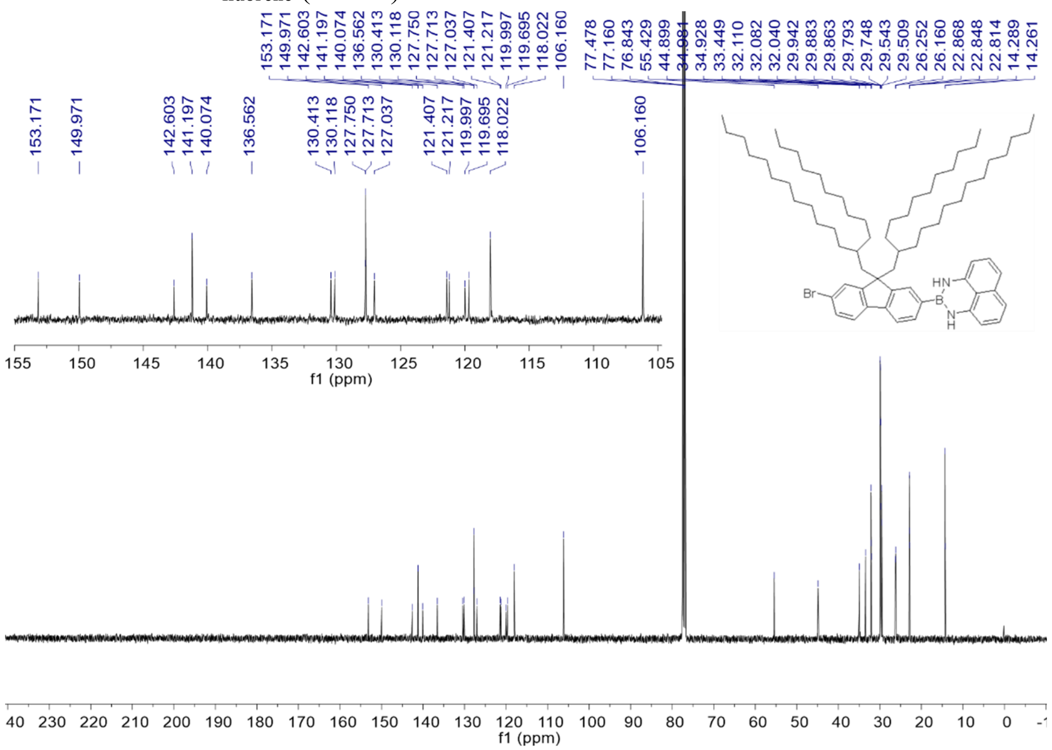

^{11}B NMR of **BBB$_{fluorene}$** ($CDCl_3$)

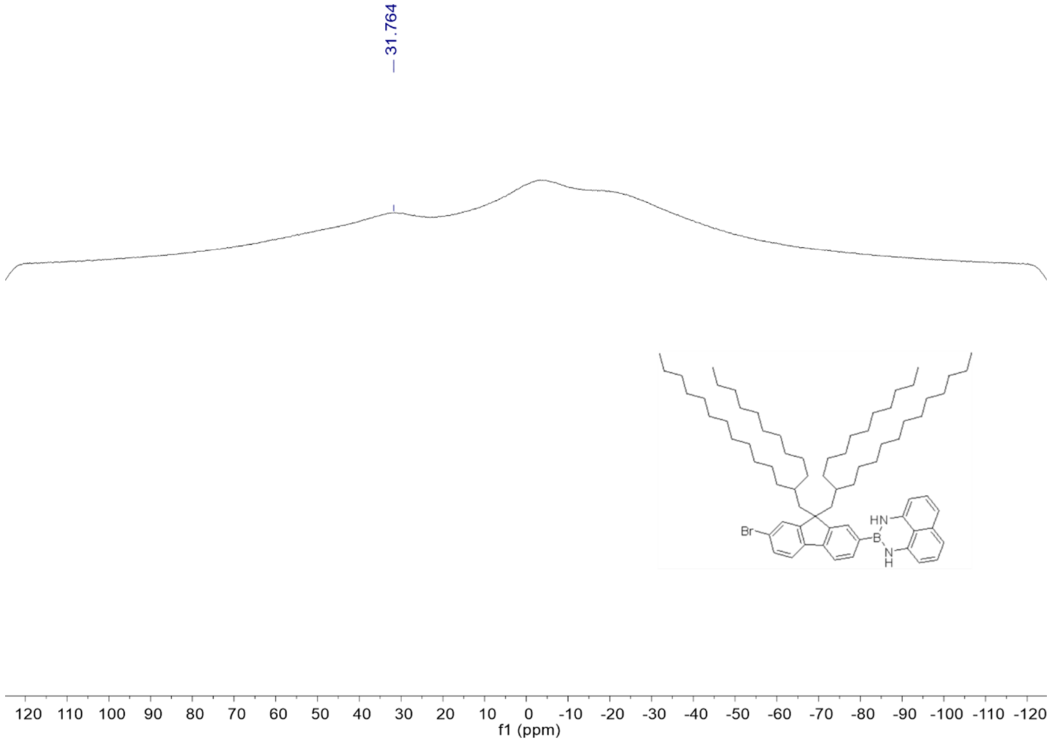

^{1}H NMR of **BBB$_{\mathbf{fluorene}}$-2** ($CDCl_3$)

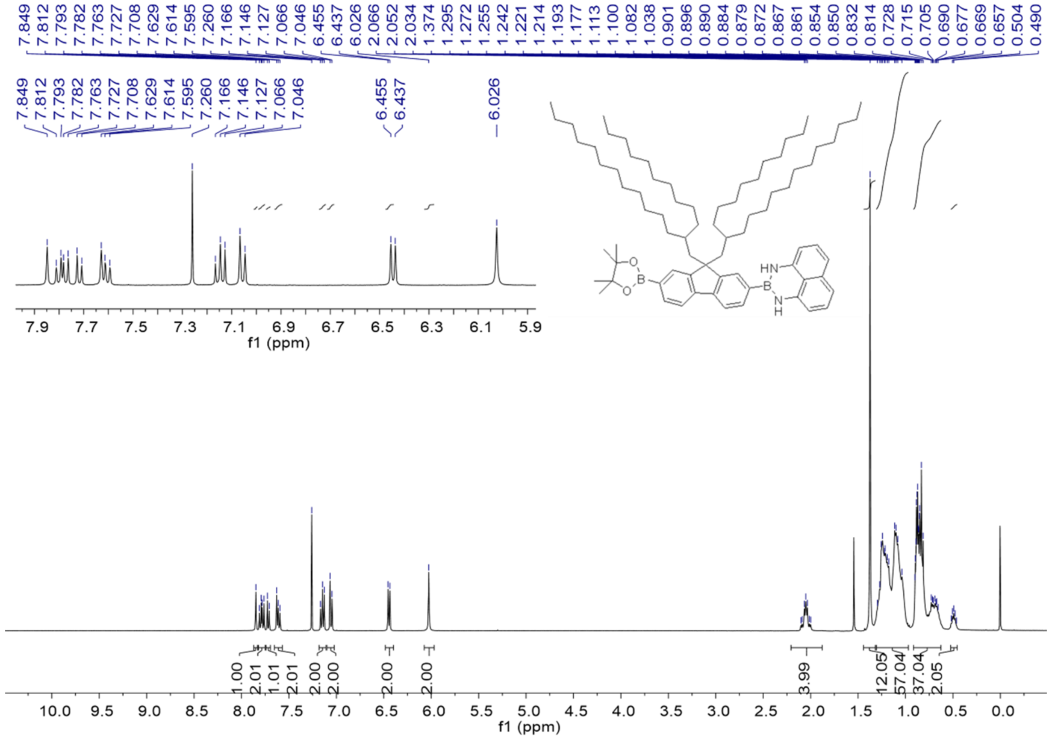

^{13}C NMR of **BBB$_{\mathbf{fluorene}}$-2** ($CDCl_3$)

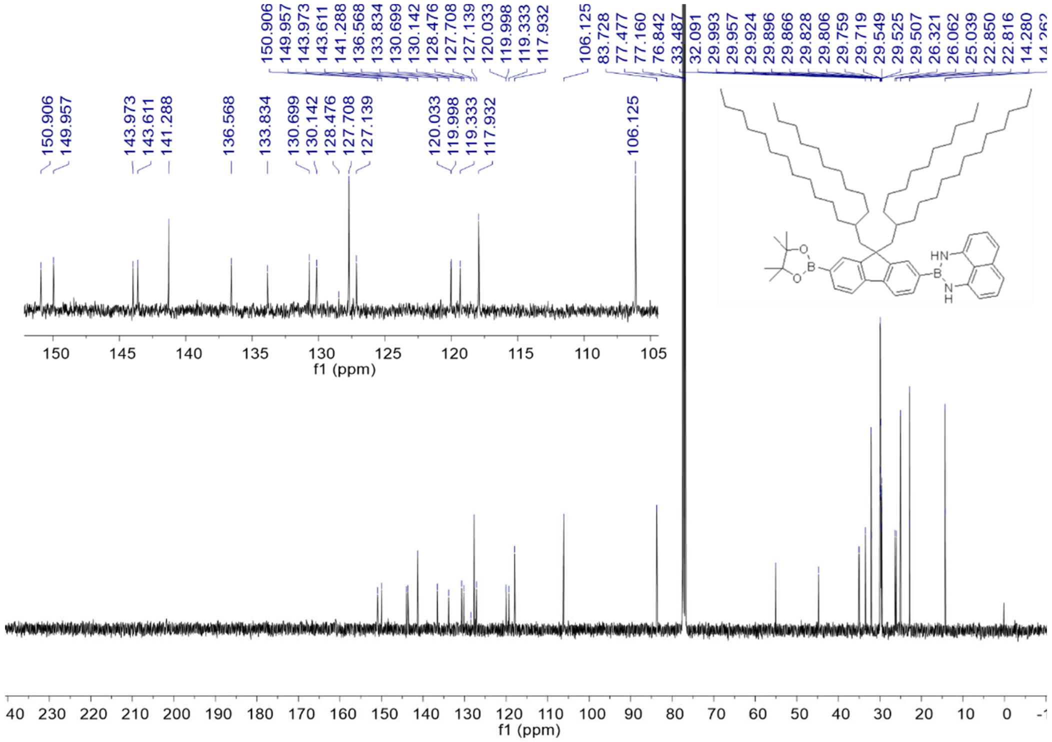

^{11}B NMR of **BBB$_{fluorene}$-2** ($CDCl_3$)

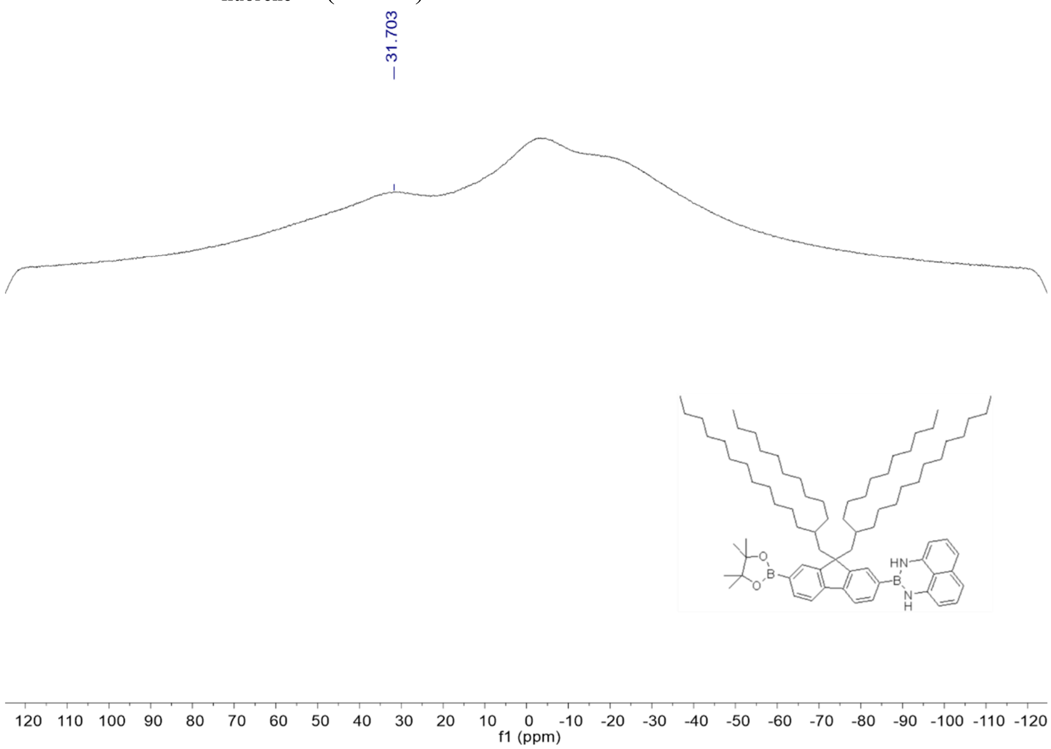

^{1}H NMR of **BOS5-1S2** ($CDCl_3$)

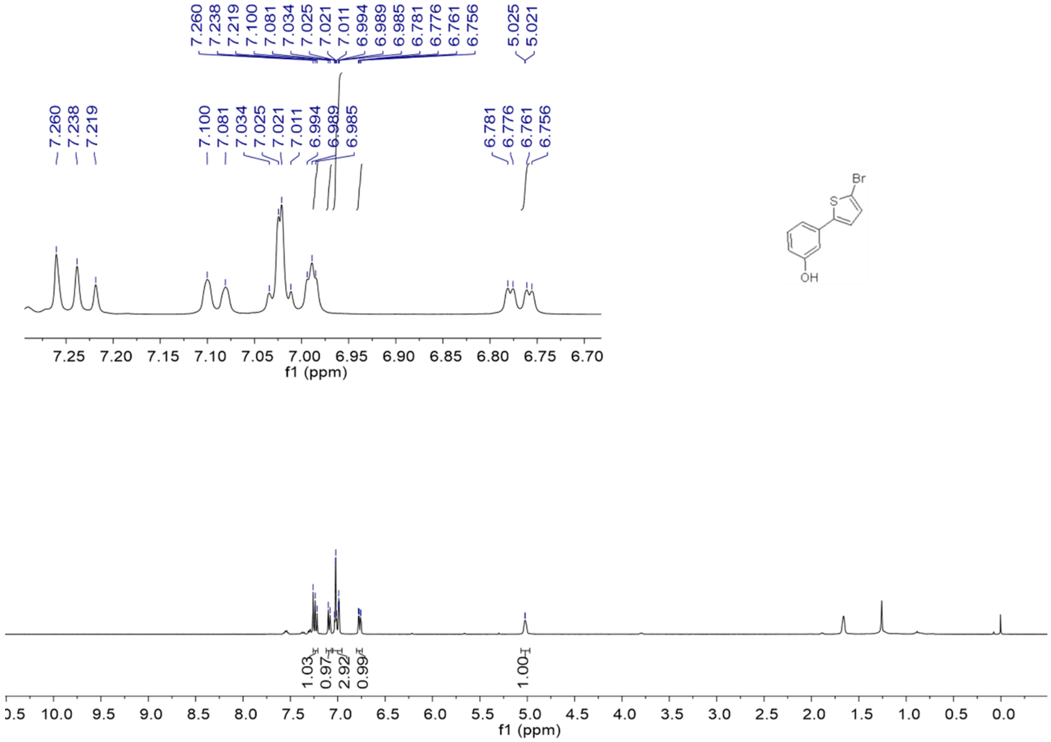

^{13}C NMR of **BOS5-1S2** ($CDCl_3$)

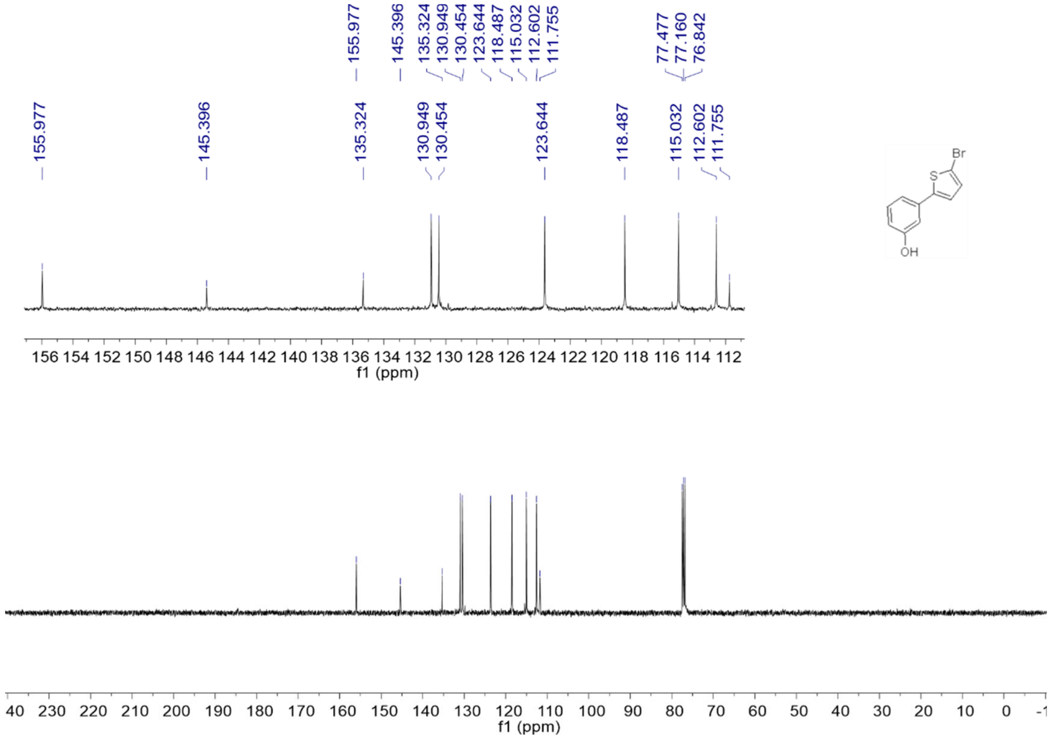

^{1}H NMR of **BOS5-1** ($CDCl_3$)

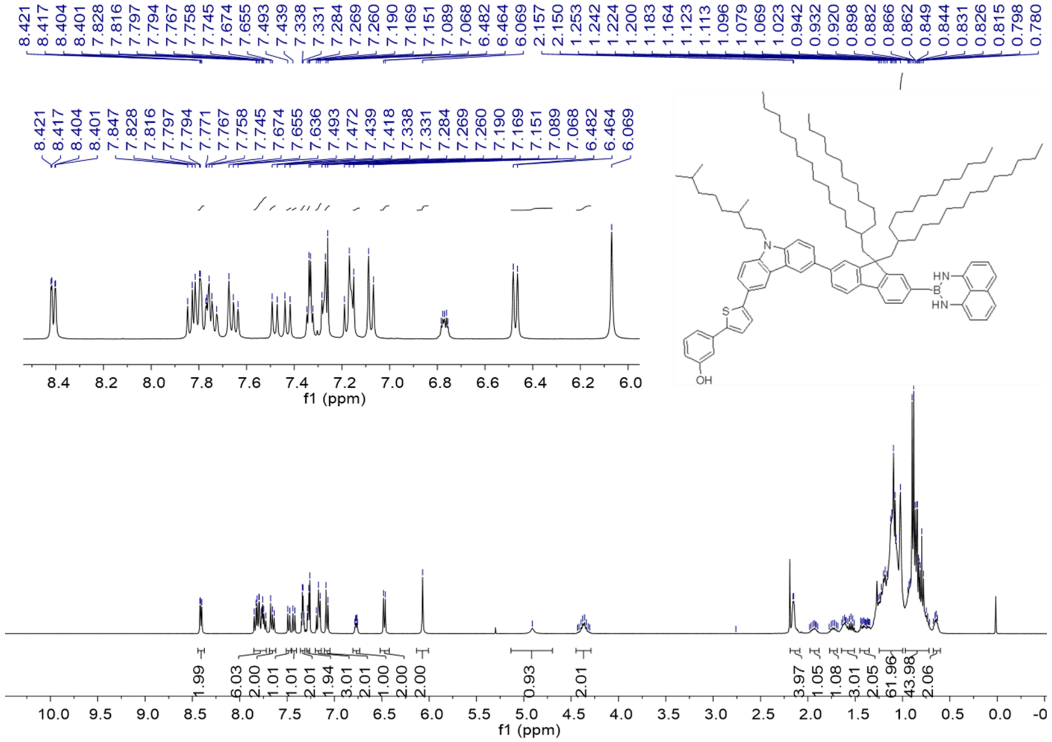

^{13}C NMR of **BOS5-1** ($CDCl_3$)

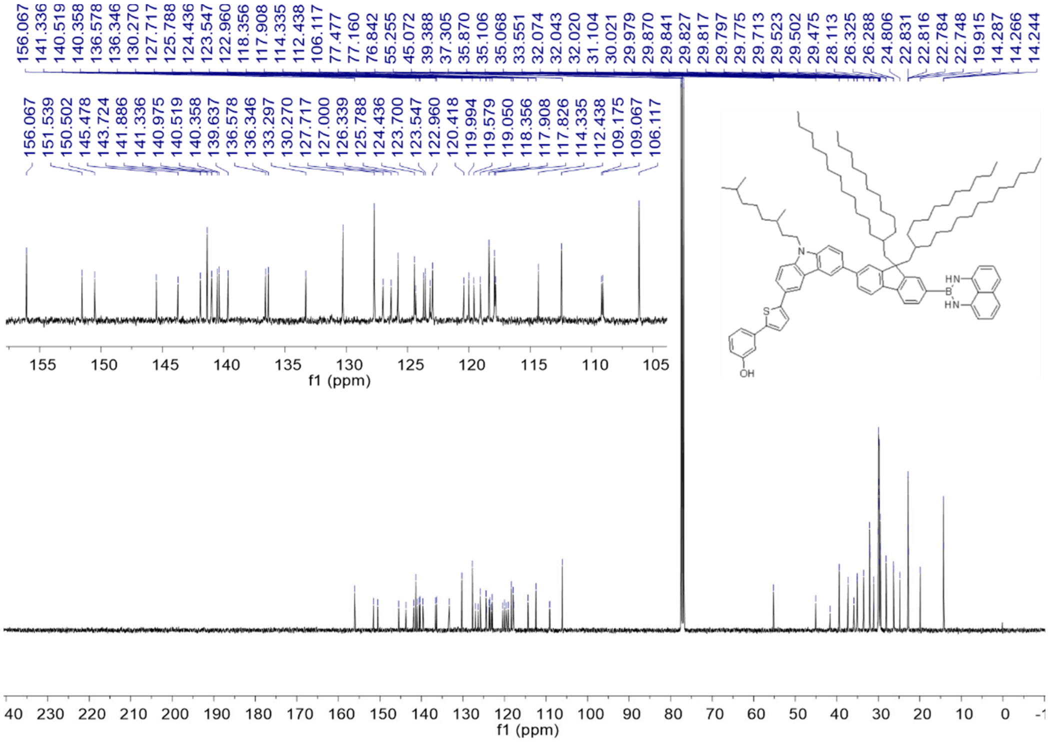

^{11}B NMR of **BOS5-1** ($CDCl_3$)

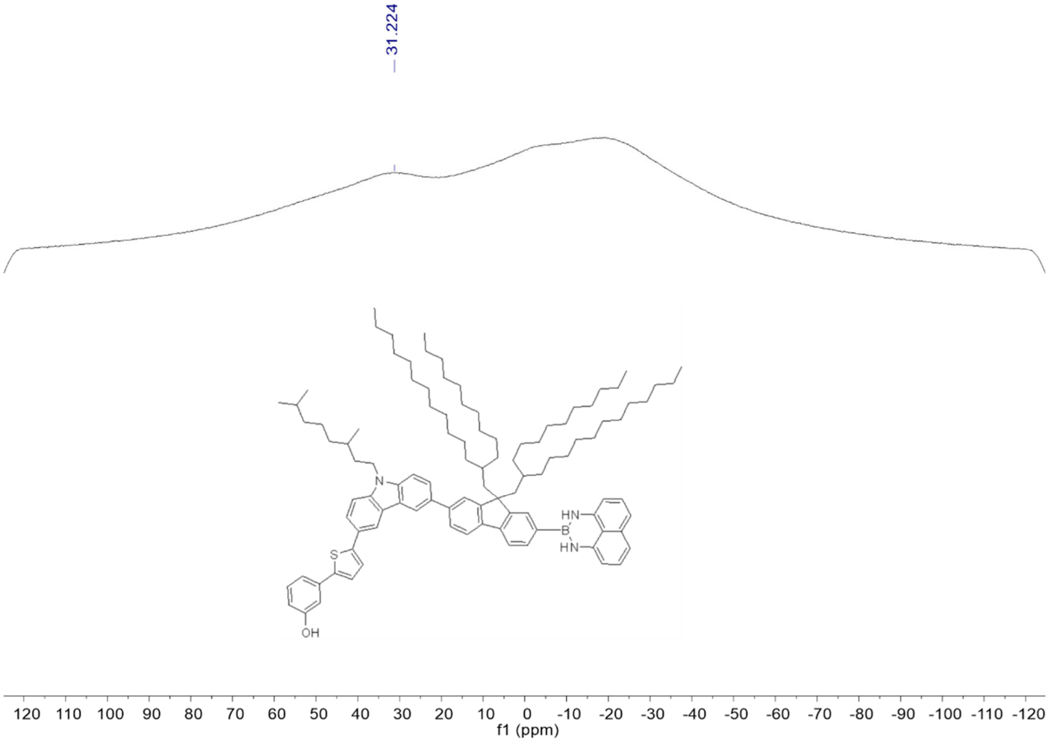

^{1}H NMR of **BOS5-1b** ($CDCl_3$)

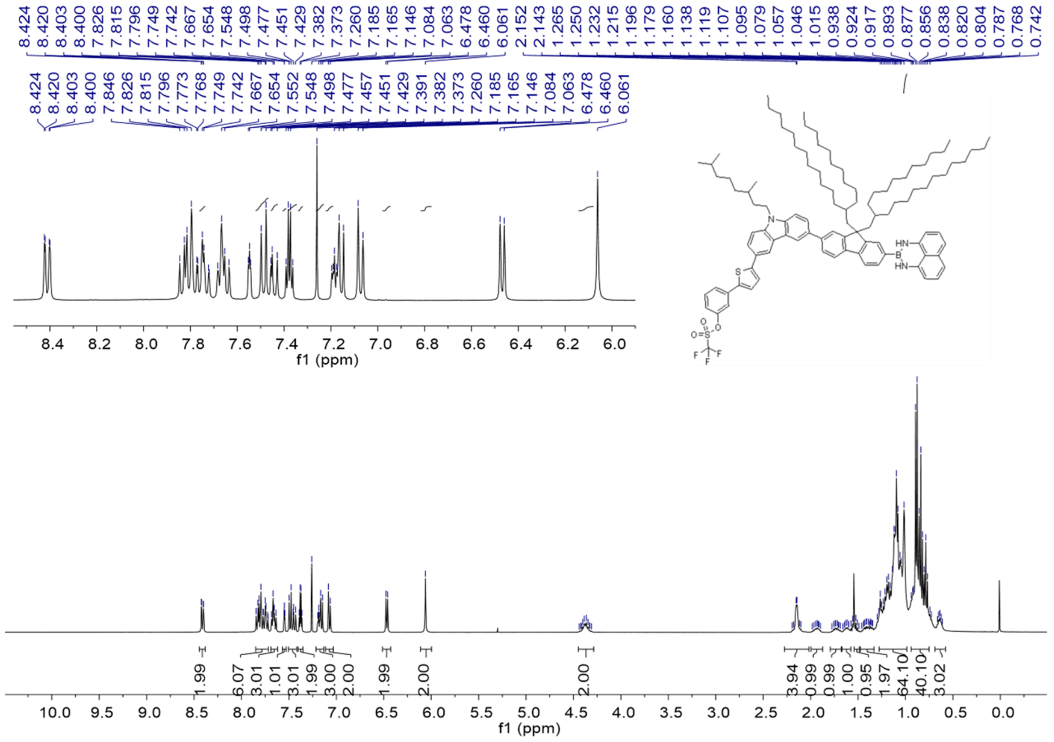

^{13}C NMR of **BOS5-1b** ($CDCl_3$)

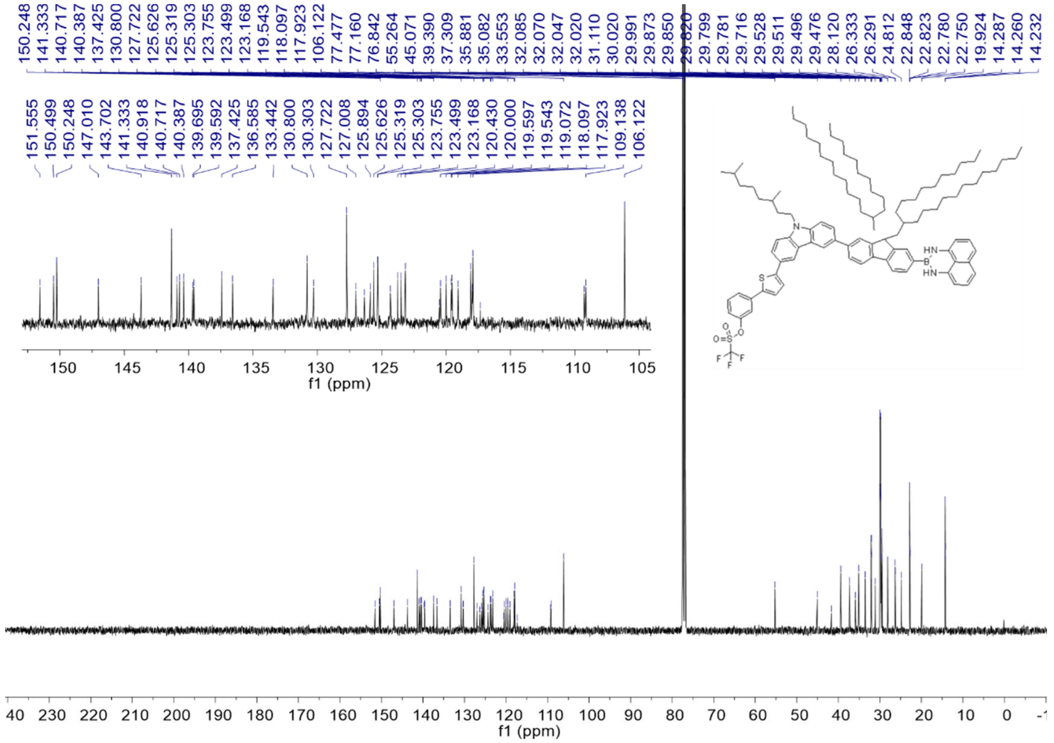

^{11}B NMR of **BOS5-1b** ($CDCl_3$)

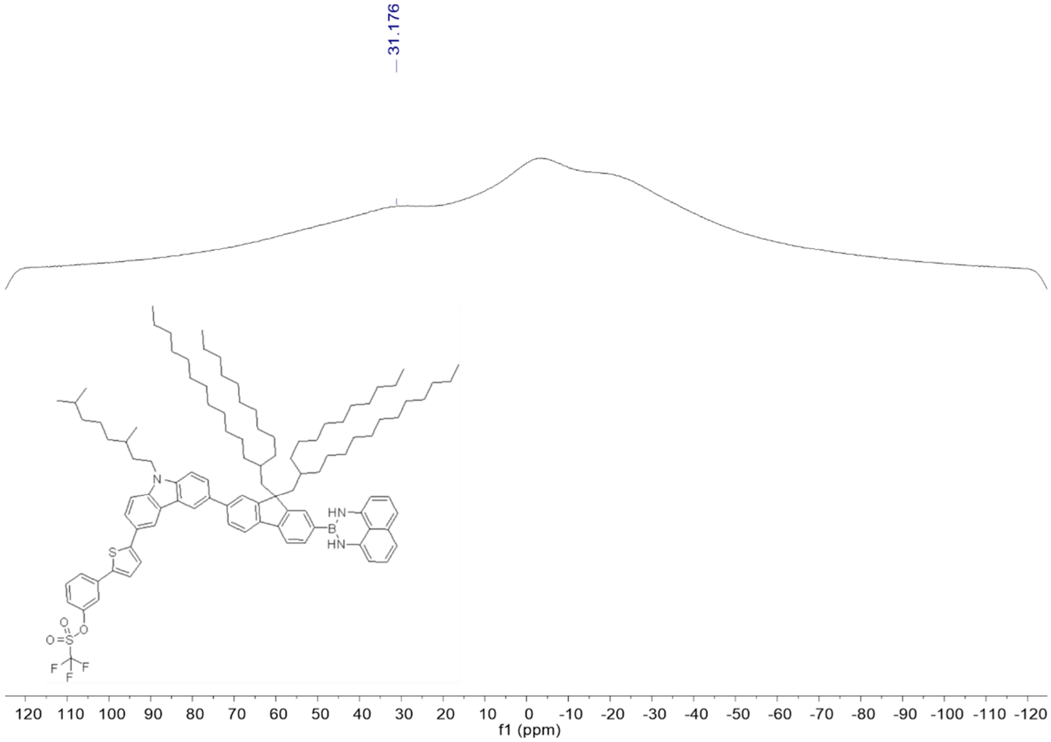

^{19}F NMR of **BOS5-1b** ($CDCl_3$)

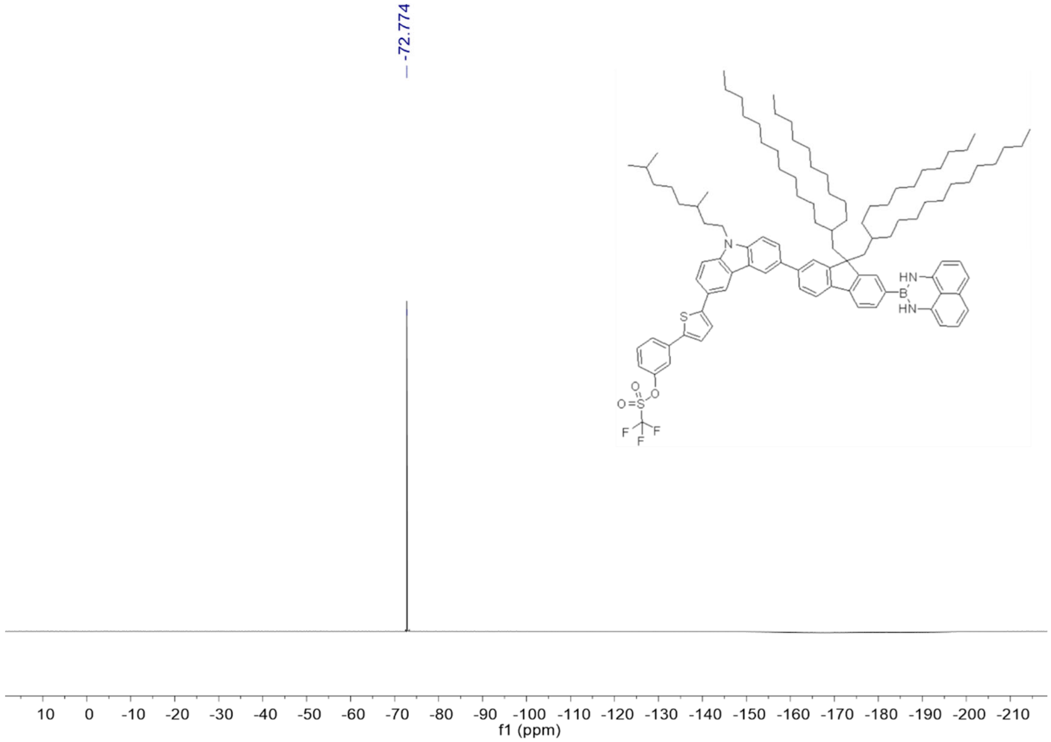

^{1}H NMR of **BOS5-2** ($CDCl_3$)

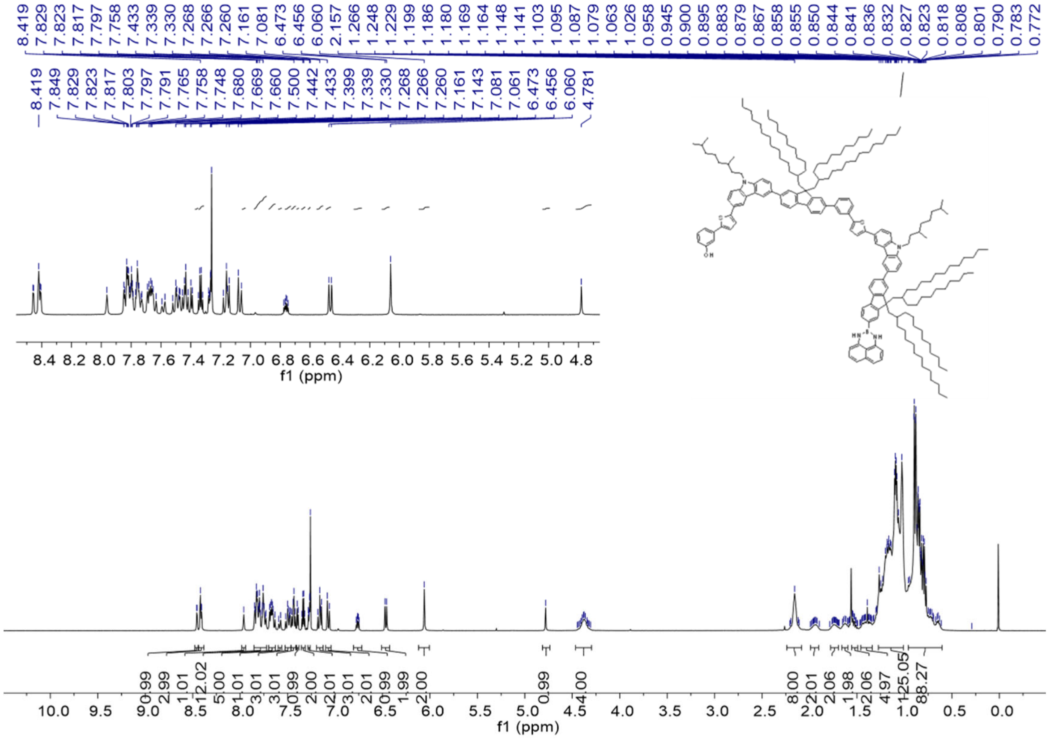

^{1}H NMR of **BOS5-4** ($CDCl_3$)

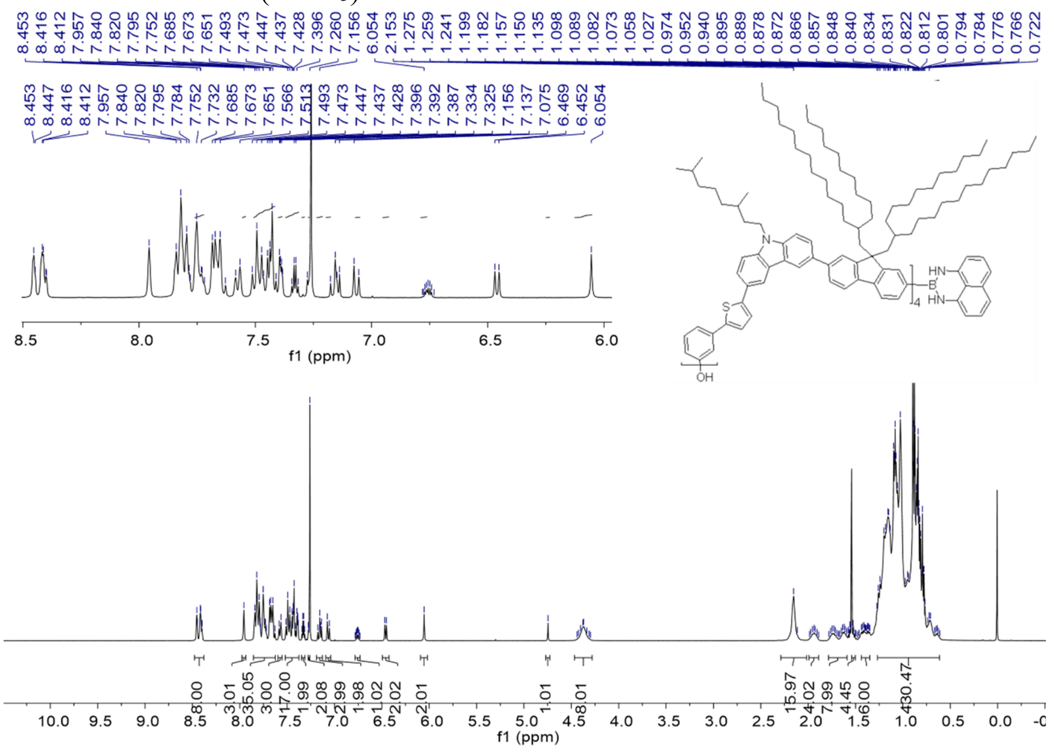

^{1}H NMR of **BOS5-8** ($CDCl_3$)

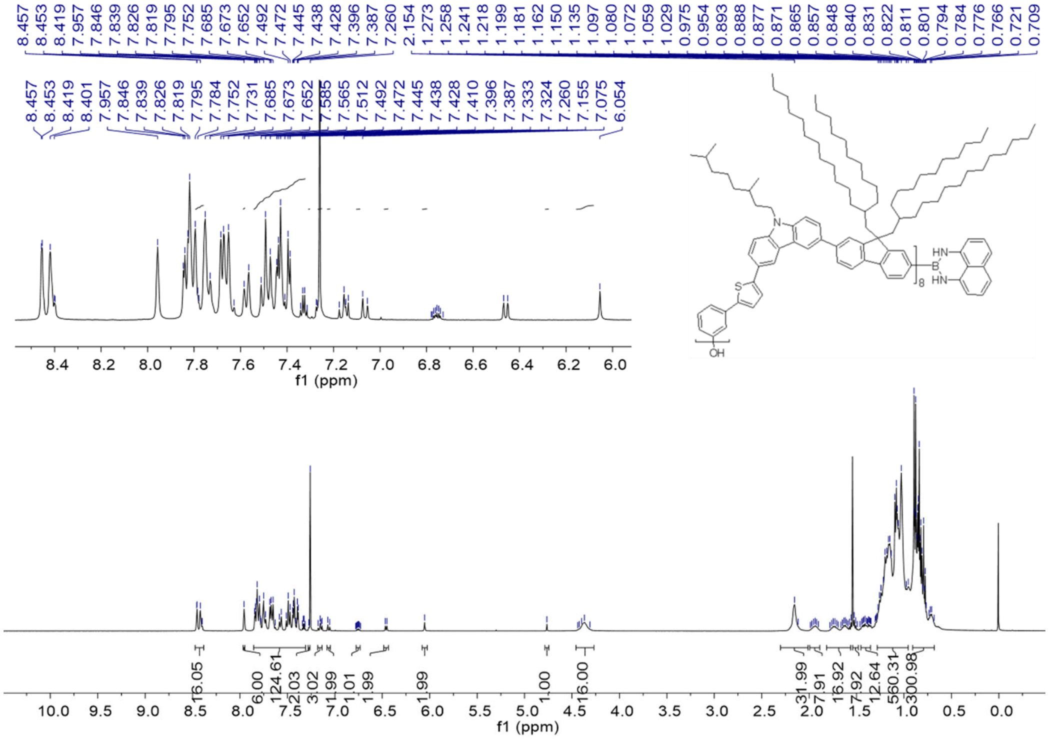

^{1}H NMR of **BOS5-16** ($CDCl_3$)

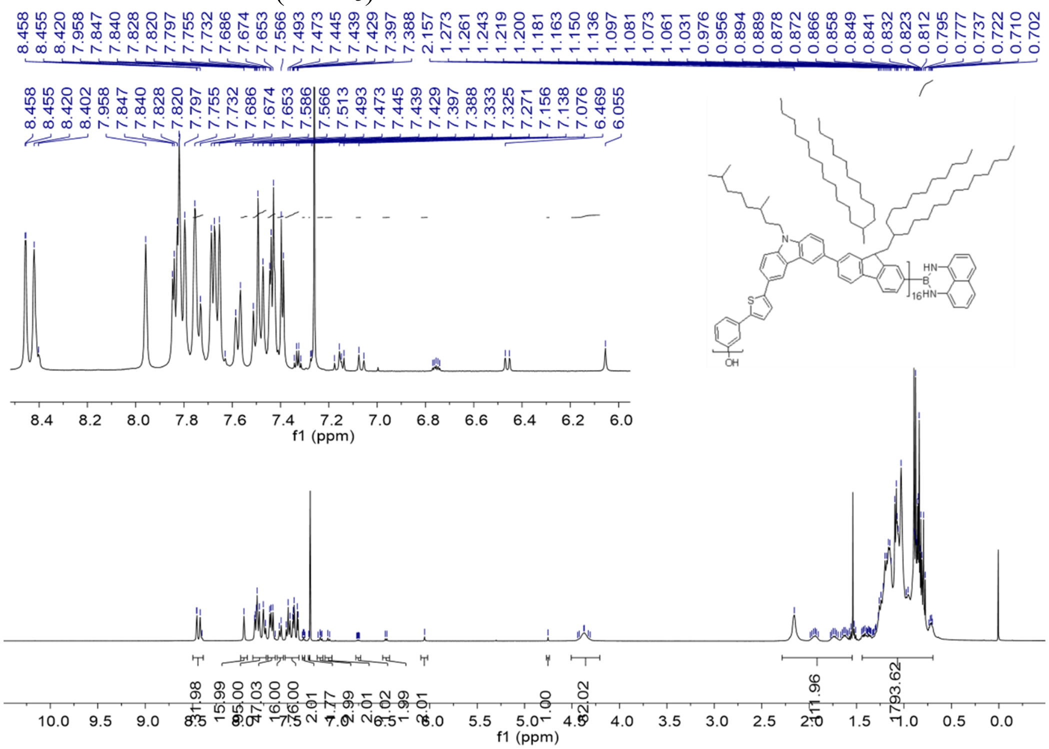

^{1}H NMR of **BOS5-32** ($CDCl_3$)

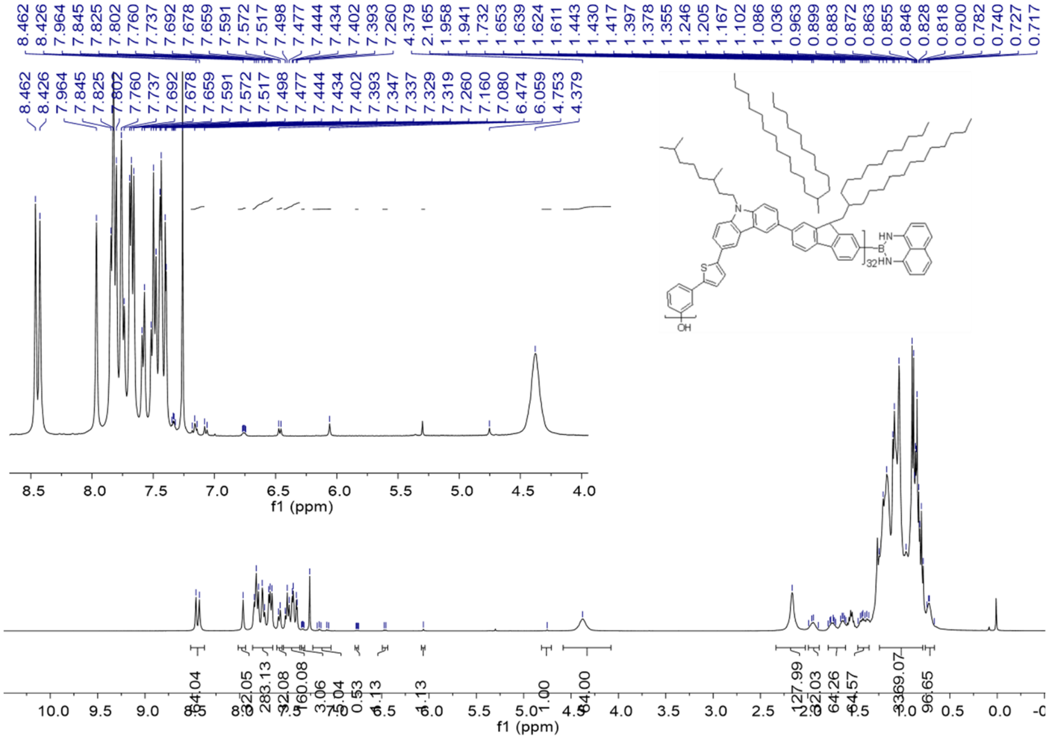

^{1}H NMR of **BOS6-1** ($CDCl_3$)

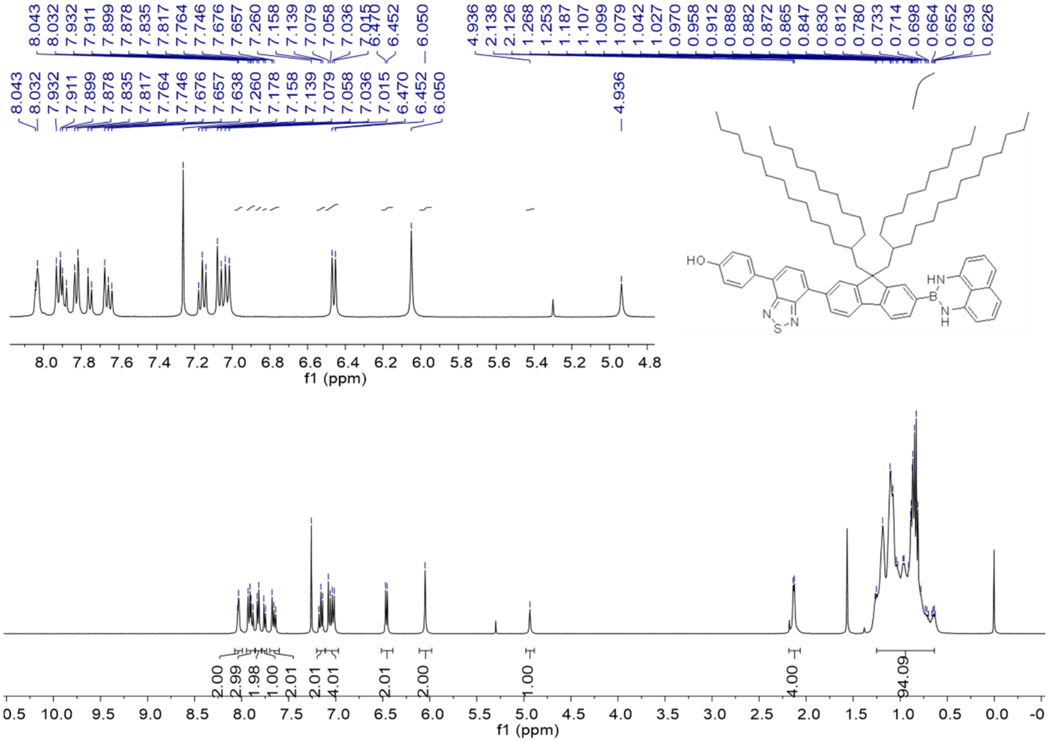

^{13}C NMR of **BOS6-1** ($CDCl_3$)

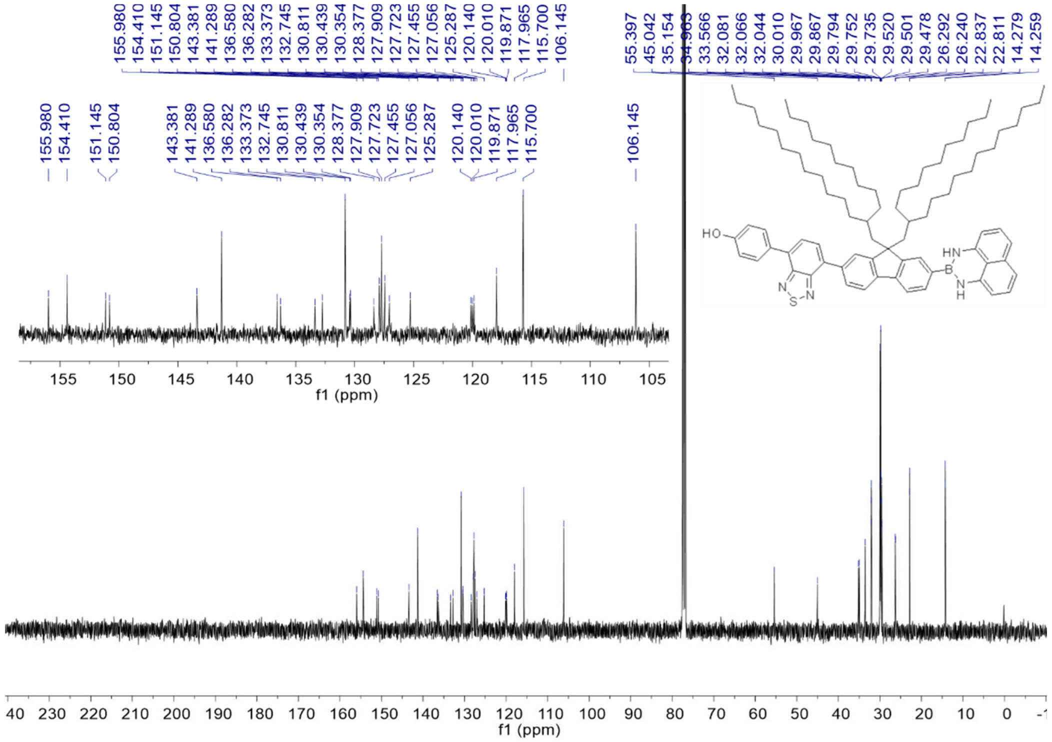

^{11}B NMR of **BOS6-1** ($CDCl_3$)

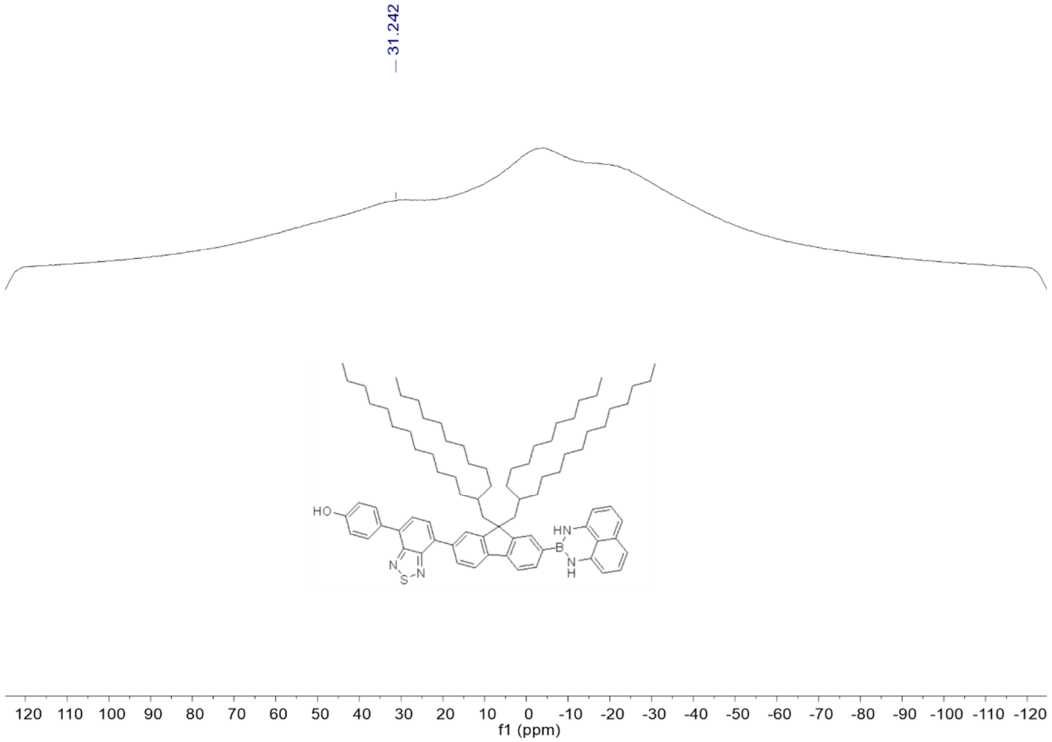

^{1}H NMR of **BOS6-1b** ($CDCl_3$)

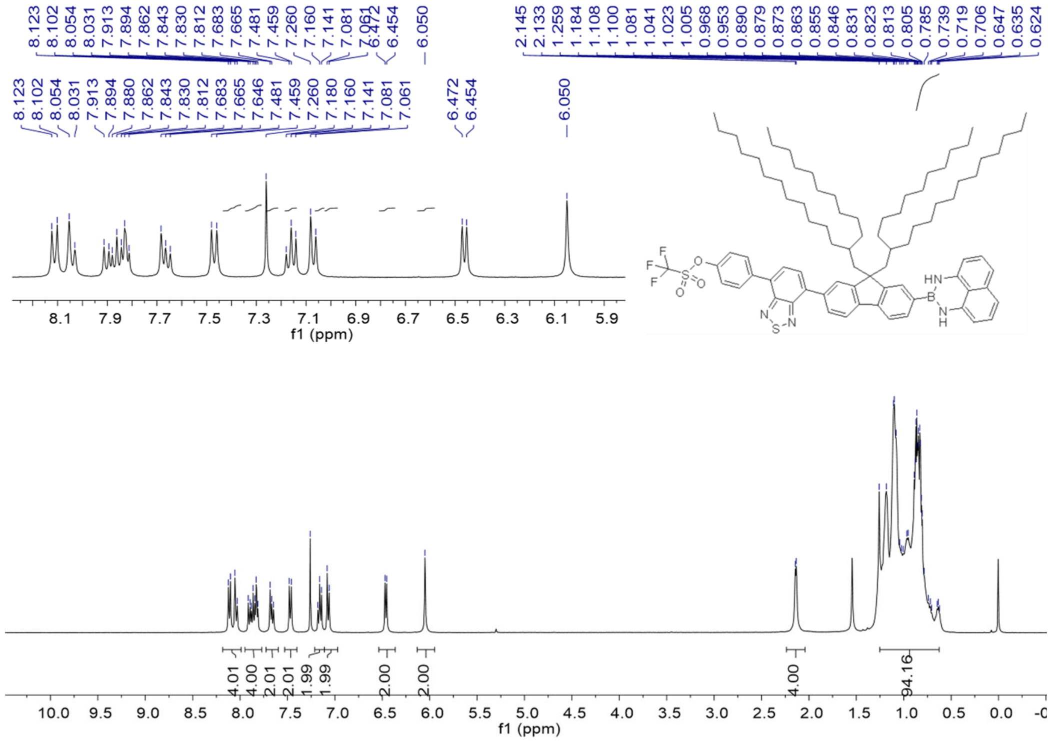

^{13}C NMR of **BOS6-1b** ($CDCl_3$)

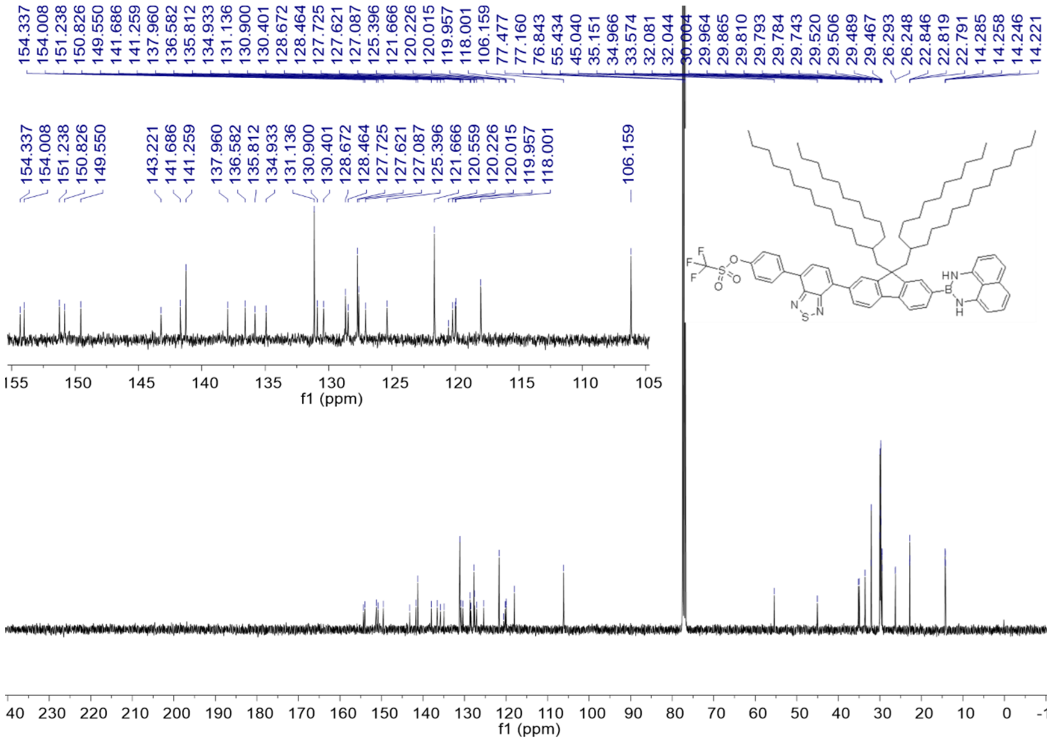

^{11}B NMR of **BOS6-1b** ($CDCl_3$)

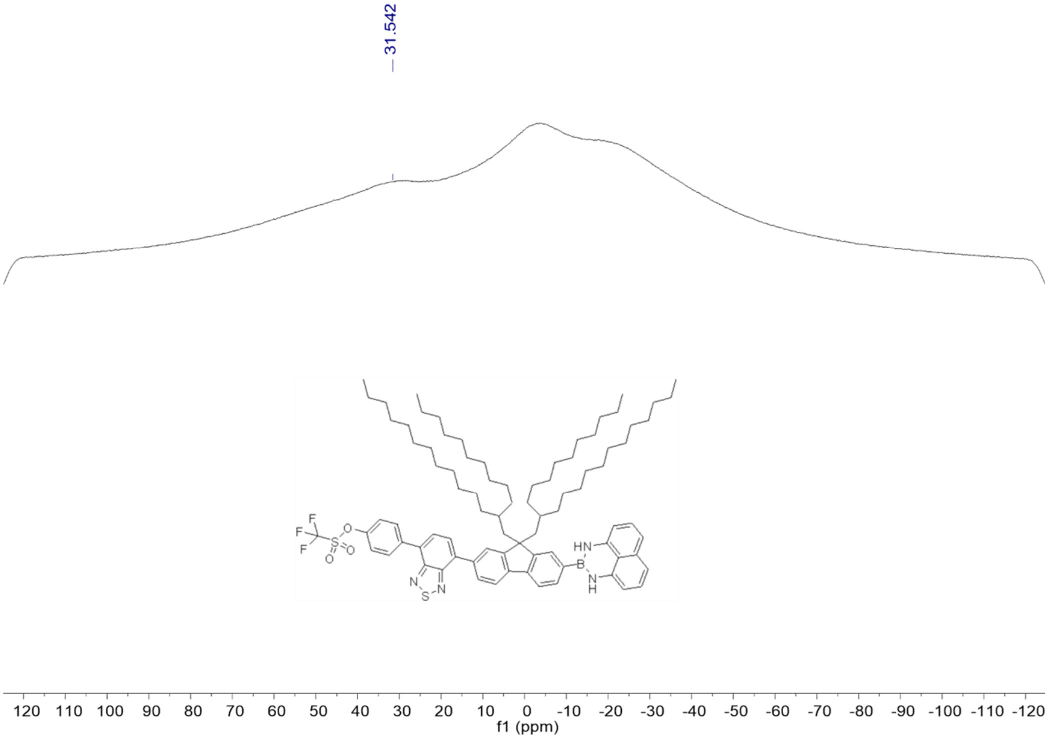

^{19}F NMR of **BOS6-1b** ($CDCl_3$)

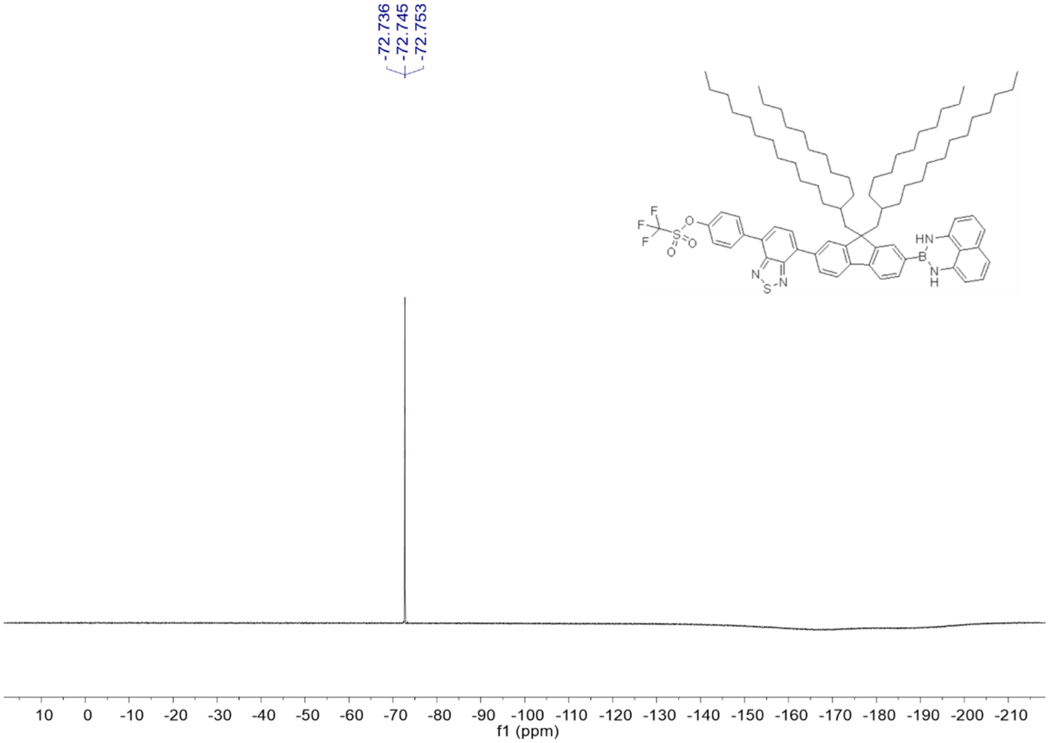

^{1}H NMR of **BOS6-2** ($CDCl_3$)

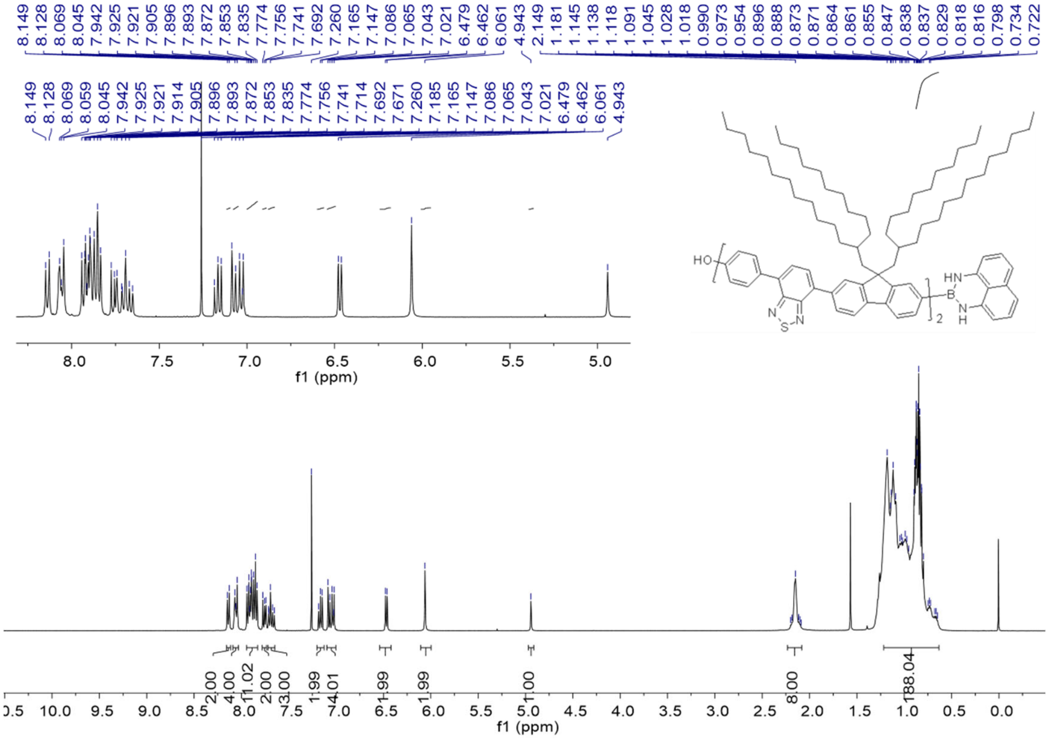

^{13}C NMR of **BOS6-2** ($CDCl_3$)

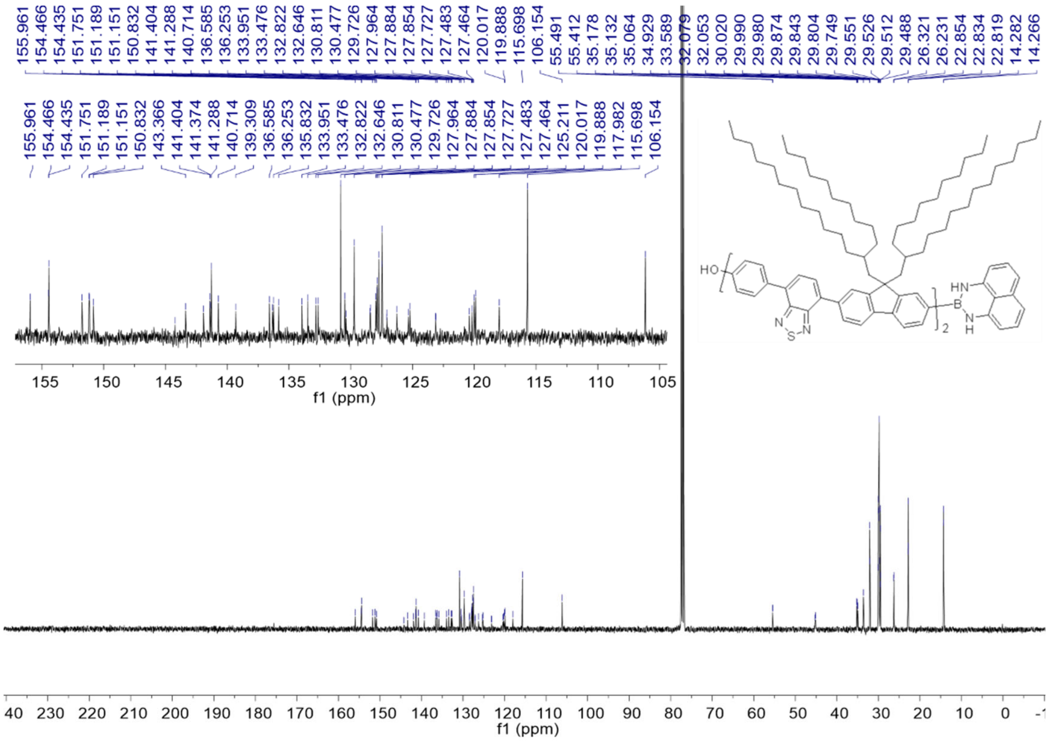

^{11}B NMR of **BOS6-2** ($CDCl_3$)

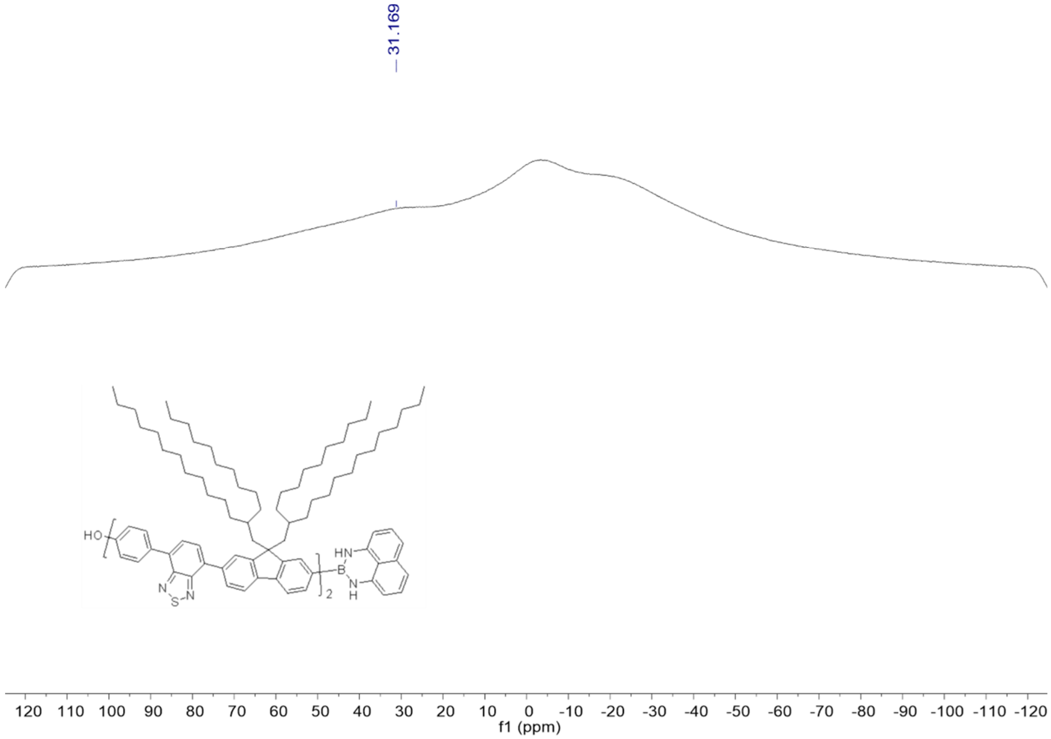

^{1}H NMR of **BOS6-4** ($CDCl_3$)

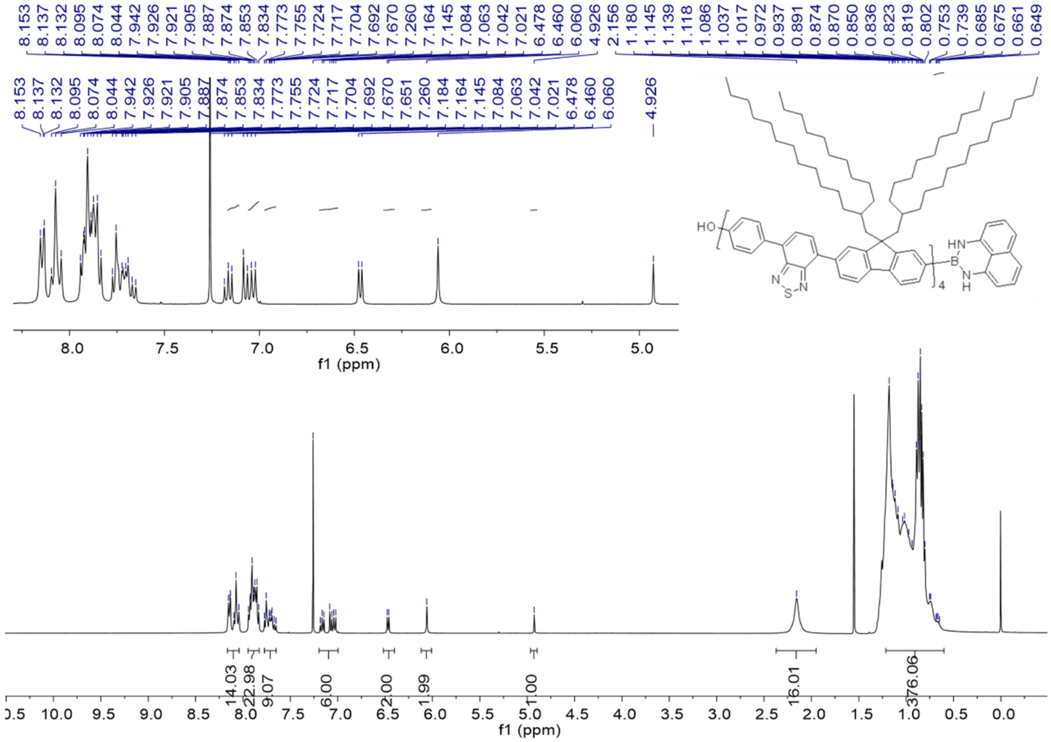

^{1}H NMR of **BOS6-8** ($CDCl_3$)

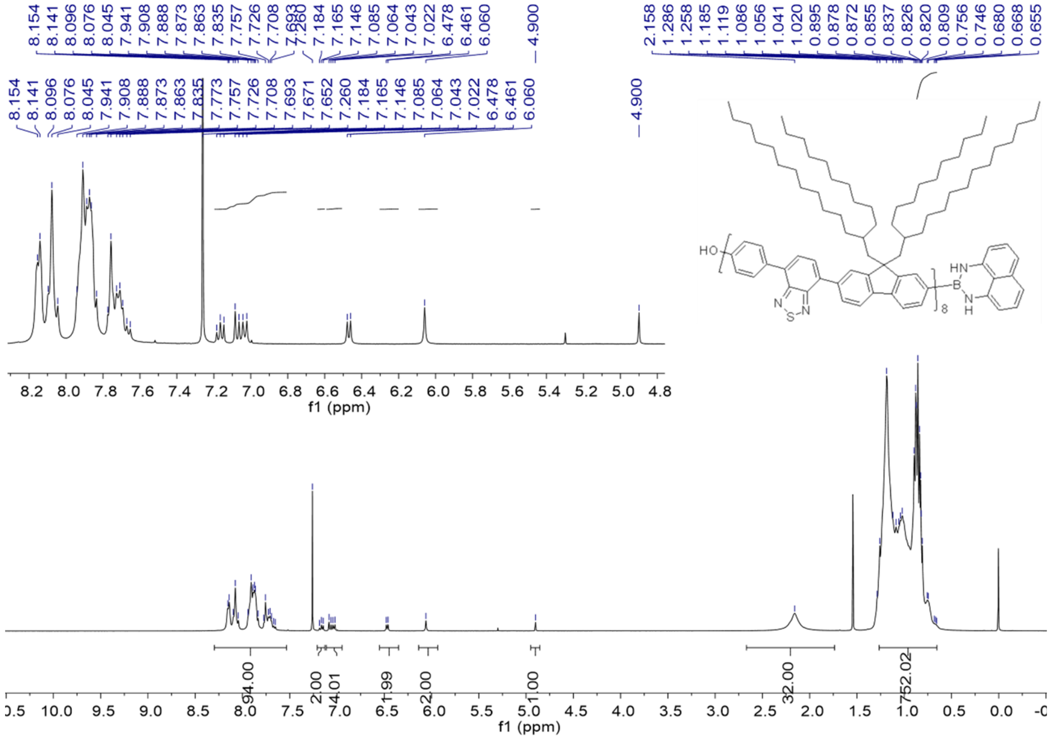

^{1}H NMR of **BOS6-16** ($CDCl_3$)

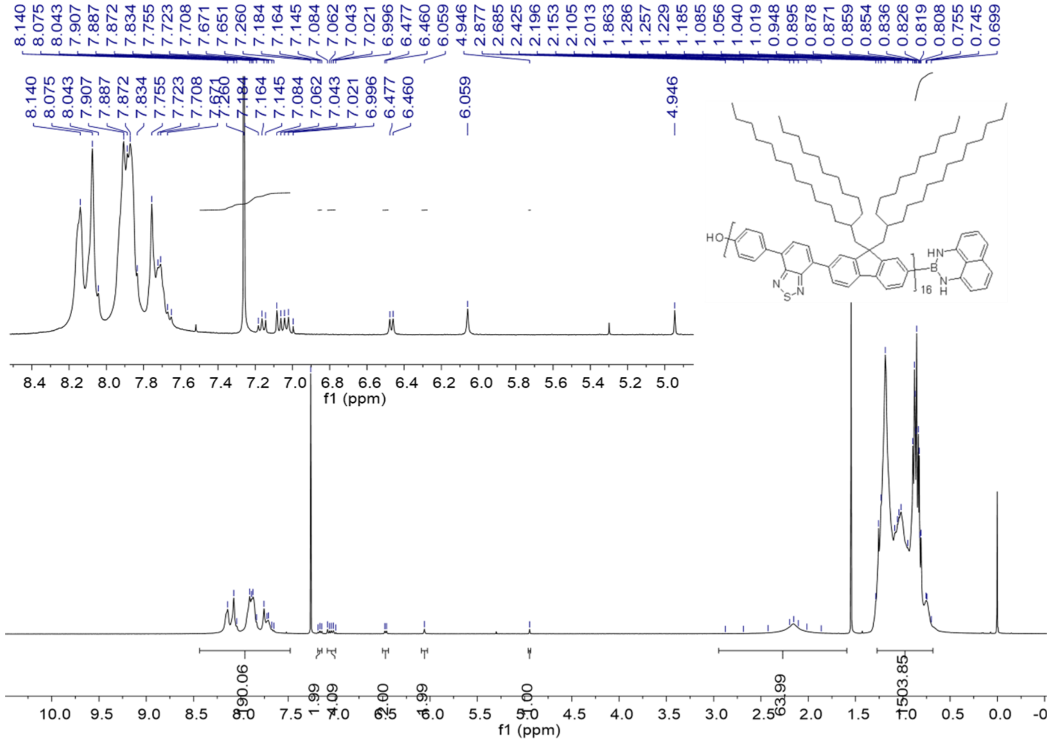

^{1}H NMR of **BOS6-32** ($CDCl_3$)

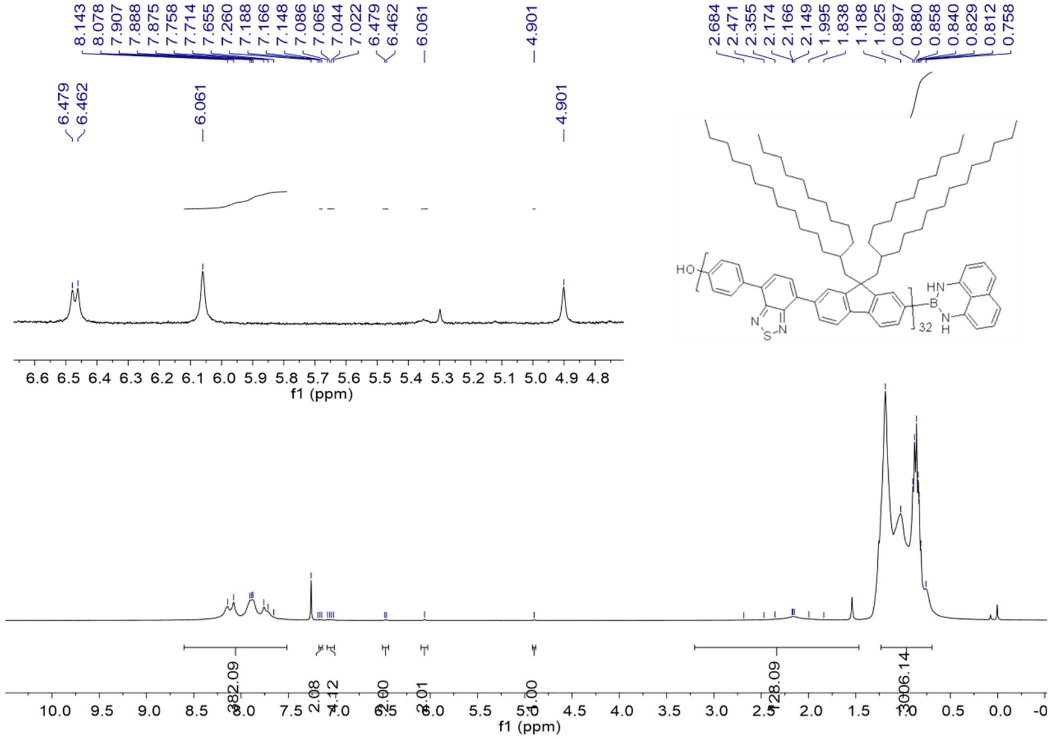

^{1}H NMR of **BOS(5,6)-16** ($CDCl_3$)

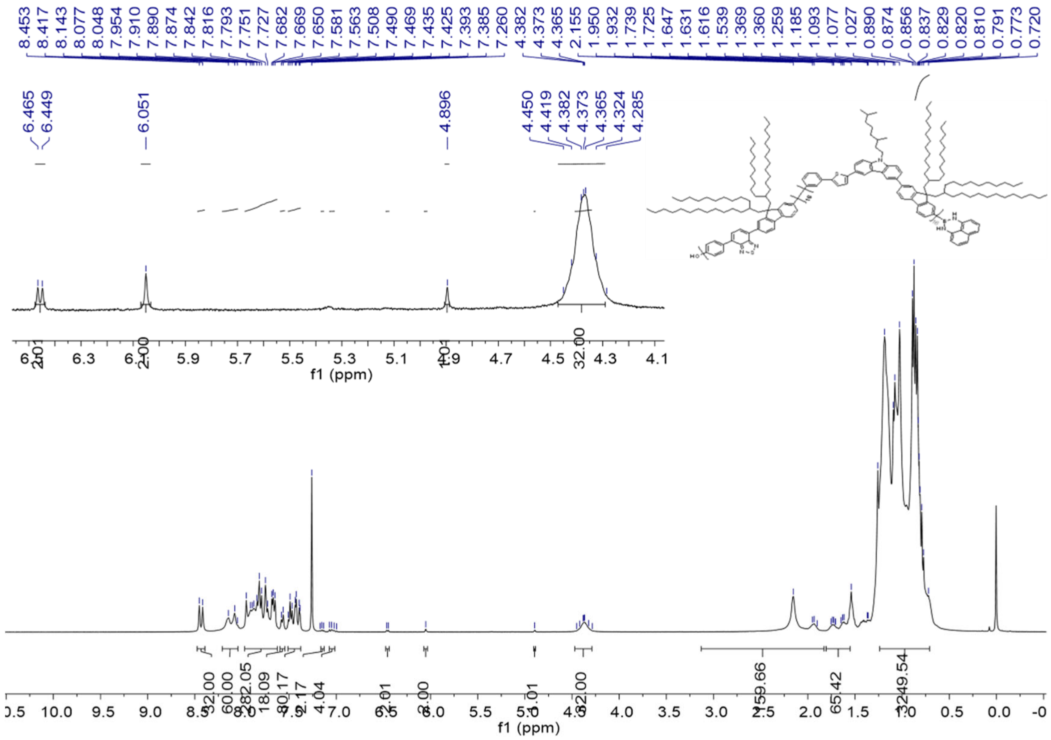

Supplement: Supplementary file 1 — ja3c08143_si_001.pdf [file ja3c08143_si_001.pdf]
